# 5’‐Methylthioadenosine Metabolic Reprogramming Drives H3K79 Monomethylation‐Mediated PAK2 Upregulation to Promote Cadmium‐Induced Breast Cancer Progression by Impairing Autophagic Flux

**DOI:** 10.1002/advs.202500941

**Published:** 2025-09-08

**Authors:** Jingdian Li, Ping Deng, Tengfei Fan, Yuchen Qu, Miduo Tan, Yidan Liang, Peng Gao, Yongchun Peng, Mingke Qin, Sheng Jie, Rongrong Hao, Liting Wang, Lei Zhang, Chunhai Chen, Mindi He, Qinlong Ma, Yan Luo, Li Tian, Jia Xie, Mengyan Chen, Rui Tian, Min Li, Zhengping Yu, Zhou Zhou, Huifeng Pi

**Affiliations:** ^1^ Department of Occupational Health (Key Laboratory of Electromagnetic Radiation Protection Ministry of Education) Army Medical University (Third Military Medical University) Chongqing 400038 China; ^2^ State Key Laboratory of Trauma and Chemical Poisoning Army Medical University Chongqing 400038 China; ^3^ Department of Oral and Maxillofacial Surgery The Second Xiangya Hospital of Central South University Changsha Hunan 410007 China; ^4^ Department of Breast Surgery Digestive Disease Medical Center Zhuzhou Hospital Affiliated to Xiangya School Medicine Central South University Zhuzhou Hunan 412000 China; ^5^ School of Medicine Guangxi University Guangxi Zhuang Autonomous Region Nanning 530004 China; ^6^ Biomedical Analysis Center Army Medical University Chongqing 400038 China; ^7^ Center for Neurointelligence School of Medicine Chongqing University Chongqing 400030 China

**Keywords:** 5′‐methylthioadenosine, autophagy, breast cancer, cadmium, H3K79me1

## Abstract

Cadmium (Cd) is a heavy metal that exhibits strong carcinogenic properties and promotes breast cancer (BC) progression. Autophagic flux dysfunction is involved in Cd‐induced BC progression, but the underlying molecular mechanisms remain unclear. Here, it is observed that impaired autophagic flux and metabolic reprogramming are notable features related to Cd‐induced proliferation, migration, and invasion in BC cell lines, including T‐47D and MCF‐7 cells. Through the integration of metabolomics, proteomics, and ingenuity pathway analysis, a metabolite–protein regulatory network is constructed, which revealed that 5′‐methylthioadenosine (MTA)‐mediated metabolic reprogramming plays a core regulatory role in the epigenetic‒autophagy axis involved in Cd‐induced autophagic flux impairment and BC progression. Mechanistically, Cd‐induced MTA depletion specifically increased DOT1L methyltransferase activity and H3K79me1 levels in the *PAK2* promoter region, inducing the expression of *PAK2*, which contributed to the autophagic flux blockade required for BC progression in Cd‐exposed BC cells and transgenic MMTV‐ErbB2 mice. Clinically, a significant negative correlation is also verified between MTA levels and TNM stage in BC patients; that is, advanced‐stage tumors exhibited notably lower MTA levels than early‐stage tumors. Thus, the study provides insights into metabolism‒epigenetic crosstalk in the context of Cd‐induced BC progression and highlights the importance of considering environmental factors in cancer healthcare.

## Graphical abstract

Cadmium, a carcinogenic heavy metal, drives breast cancer progression via metabolic reprogramming and autophagic flux disruption. Multi‐omics revealed cadmium‐induced 5'‐methylthioadenosine depletion activates DOT1L‐mediated H3K79me1 at *PAK2* promoter, upregulating *PAK2* to block autophagy and driving malignancy. Clinically, 5'‐methylthioadenosine levels show a negative correlation with TNM stages in breast cancer patients. This study elucidates metabolism‐epigenetic crosstalk in environmental carcinogen‐promoted breast cancer progression.

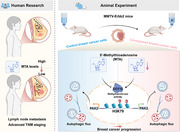

## 1 Introduction

Cadmium (Cd), a naturally occurring heavy metal, is widely used across various industrial and agricultural sectors, including electroplating, metallurgical processes, and fertilizer production.^[^ ^1^, ^2^ ^]^ It enters the human body through multiple pathways, such as bioaccumulation in the food chain, ingestion of contaminated drinking water, and tobacco smoking, leading to its accumulation within tissues.^[^ ^3^ ^]^ Owing to its well‐documented adverse effects on human health, Cd has been classified as a Group 1 carcinogen.^[^ ^4^, ^5^ ^]^ As the metal most extensively investigated in relation to breast cancer (BC), Cd exposure has been shown to increase the risk of BC, as evidenced by cohort and case‒control studies.^[^ ^6^, ^7^, ^8^, ^9^ ^]^ Another study established a clear association of elevated blood Cd levels with distant metastasis and advanced tumor stage in BC patients, and the blood Cd level was identified as an independent prognostic risk factor.^[^ ^10^ ^]^ In addition, our recent work confirmed that Cd exposure significantly promoted the proliferation, metastasis, and epithelial‒mesenchymal transition of BC cells in MMTV‐ErbB2 mice.^[^ ^11^, ^12^ ^]^ Overall, these results indicate that Cd exposure is one of the major causes of BC tumorigenesis and metastasis.

Autophagy is a cellular process in which energy and materials are recycled through the degradation of damaged organelles and the elimination of abnormally aggregated macromolecules.^[^ ^13^ ^]^ Abnormal autophagy is closely associated with the development and progression of BC, particularly in the early stages of the disease.^[^ ^14^, ^15^ ^]^ Defects in autophagy may lead to acceleration of BC progression related to increased DNA damage and genomic instability.^[^ ^16^, ^17^ ^]^ Notably, our previous study indicated that impaired autophagy significantly contributes to Cd‐induced BC cell proliferation, migration, and invasion.^[^ ^18^ ^]^ However, the intricate mechanisms underlying Cd‐triggered autophagy impairment in the context of BC progression have not been fully elucidated.

Metabolic reprogramming has emerged as a hallmark of malignancy.^[^ ^19^, ^20^ ^]^ Specific metabolic characteristics may render certain cancers particularly reliant on autophagy to acquire the substrates and energy necessary for their survival.^[^ ^21^ ^]^ On the other hand, metabolic processes can affect autophagic flux in the context of tumor progression. For example, an increase in asparagine levels promotes activation of the mTOR pathway, which consequently regulates autophagy to meet the energy and metabolic demands of proliferating liver cancer cells.^[^ ^22^ ^]^ Additionally, glutamine deprivation induces autophagy during the development of brain tumors through the phosphorylation of Beclin1 at serine 30.^[^ ^23^ ^]^ Increasing evidence indicates that the coordinated regulation of metabolic reprogramming supports the existence of a complex autophagy network involved in tumor adaptation and progression. Nevertheless, limited attention has been given to exploring the regulatory effects of core metabolites on autophagic flux in BC cells exposed to Cd at the molecular level.

5′‐Methylthioadenosine (MTA), a sulfur‐containing nucleoside, functions as a critical metabolic intermediate in both the methionine cycle and polyamine biosynthesis, and serves as a substrate source for the purine salvage pathway.^[^ ^24^, ^25^ ^]^ Substantial evidence links MTA and its metabolic derivatives to tumor progression dynamics.^[^ ^18^, ^26^, ^27^ ^]^ Notably, hepatocellular carcinoma development models exhibit significant depletion of endogenous MTA,^[^ ^28^ ^]^ with parallel studies demonstrating its tumor‐suppressive potential through marked inhibition of proliferation and tumor burden reduction across diverse in vivo and in vitro models spanning melanoma, colon cancer, and cervical carcinoma.^[^ ^29^, ^30^, ^31^ ^]^ Intriguingly, recent studies propose an epigenetic regulatory role for MTA through potential modulation of methyltransferase activity.^[^ ^32^, ^33^ ^]^ As the sole methyltransferase that catalyzes H3K79 modification, DOT1L exhibits oncogenic properties in BC. In hormone receptor‐positive BC models, pharmacological inhibition of DOT1L effectively suppresses spheroid formation and proliferative activity in MCF7 cells.^[^ ^34^, ^35^ ^]^ Notably, targeting DOT1L has emerged as a promising therapeutic strategy against endocrine therapy‐resistant BC subtypes, revealing its potential clinical value in overcoming treatment resistance.^[^ ^36^ ^]^ Triple‐negative breast cancer (TNBC) exhibits a stem cell‐specific dependence on the DOT1L‐H3K79me2 axis, as targeted inhibition of this axis not only suppressed EMT‐driven migration and invasion in MDA‐MB‐231 cells but also substantially impaired tumor‐initiating potential and metastatic competence.^[^ ^37^, ^38^ ^]^ Orthotopic xenograft models demonstrate preclinically that DOT1L plays a pivotal role in mammary tumorigenesis, with genetic ablation markedly inhibiting both primary tumor growth and distant metastasis.^[^ ^39^ ^]^ Clinical correlation analyses have further demonstrated that elevated DOT1L expression is correlated with aggressive tumor phenotypes and reduced survival rates in BC patients.^[^ ^39^ ^]^ Despite current evidence establishing DOT1L as a master epigenetic driver in breast carcinogenesis, a knowledge gap persists regarding potential cross‐regulation between DOT1L activity and the MTA metabolic pathway.

Here, we demonstrated that impaired autophagic flux and metabolic reprogramming are notable features of Cd‐induced BC malignancy. Through the integration of metabolomics, proteomics, and ingenuity pathway analysis (IPA), a metabolite–protein regulatory network was constructed, and revealed that MTA metabolic reprogramming plays a core regulatory role in the epigenetic‒autophagy axis in the context of Cd‐induced BC malignancy. Mechanistically, quantitative histone methylation proteomics and cleavage under targets and tagmentation (CUT&Tag) technology jointly revealed that Cd‐induced MTA depletion specifically decreased the activity of the DOT1L methyltransferase and increased H3K79me1 levels in the P21‐activated kinase 2 (*PAK2*) promoter region, facilitating the expression of *PAK2*, which contributes to the autophagic flux blockage that is required for BC progression. These findings emphasize the role of MTA as an epigenetic regulator that inhibits Cd‐induced BC progression, providing new insights into environmental oncology research.

## 2 Results

### 2.1 Cd Promotes the Proliferation, Migration, and Invasion of BC Cells by Disrupting Autophagic Flux

**Figure 1:**
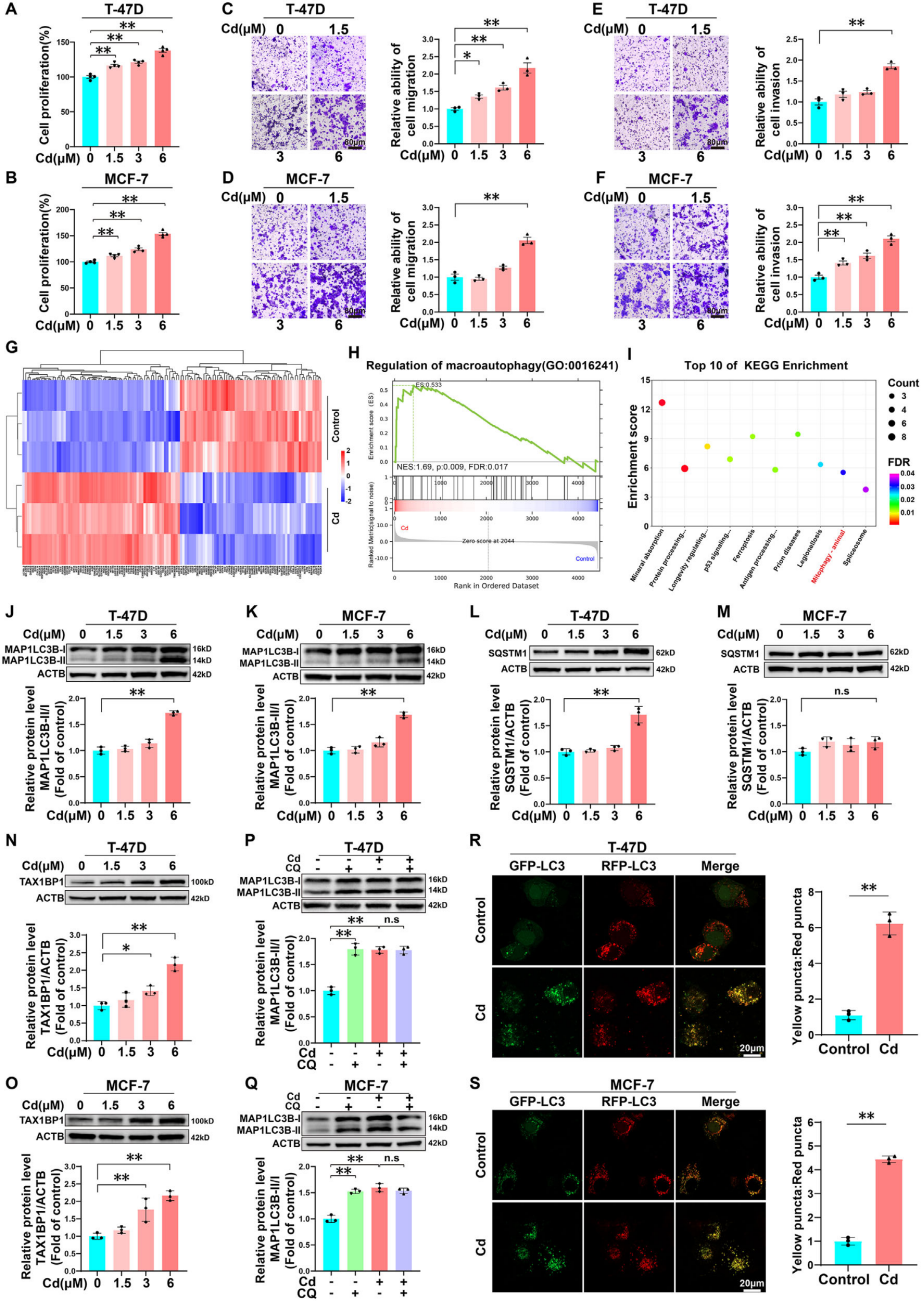
Cd exposure promotes the proliferation, migration, and invasion of T‐47D and MCF‐7 cells while disrupting autophagic flux. A, B) Proliferative activity of T‐47D and MCF‐7 cells following treatment with 0, 1.5, 3, or 6 µm Cd for 72 h. C, D) Migratory capacities of T‐47D and MCF‐7 cells following treatment with 0, 1.5, 3, or 6 µm Cd for 72 h. Scale bar: 80 µm. E, F) The invasive capacities of T‐47D and MCF‐7 cells following treatment with 0, 1.5, 3, and 6 µm Cd for 72 h. Scale bar: 80 µm. G) Heatmap of hierarchical clustering for differentially expressed proteins (DEPs) in T‐47D cells following treatment with or without 6 µm Cd for 72 h (|fold change| > 1.5 and FDR < 0.05). H) GSEA plot of DEPs involved in the regulation of macroautophagy (GO:00 16241; NES = 1.69, FDR = 0.017). I) Top 10 KEGG pathways enriched in the DEPs (mitophagy–animal: enrichment score = 5.54, FDR = 0.032). Immunoblotting and quantification of MAP1LC3B J, K), SQSTM1 L, M), and TAX1BP1 N, O) in T‐47D and MCF‐7 cells following treatment with 0, 1.5, 3, or 6 µm Cd for 72 h. P, Q) Immunoblotting and quantification of MAP1LC3B in T‐47D and MCF‐7 cells treated with or without 25 µm CQ in the absence or presence of 6 µM Cd for 72 h. ^*^ *p* < 0.05 and ^**^ *p* < 0.01 versus the control group; ns: not significant (*Tukey's HSD*). R, S) Representative images and quantification of GFP‐RFP‐LC3B puncta in T‐47D and MCF‐7 cells following treatment with or without 6 µM Cd for 72 h. Scale bar: 20 µm. ^**^ *p* < 0.01 versus the control group.

To investigate the effects of Cd on BC progression, T‐47D and MCF‐7 cells were exposed to 0, 1.5, 3, and 6 µm Cd for 72 h. CCK‐8 assays revealed dose‐dependent increases in proliferative activity in both cell lines (**Figure** **1**A,B). Transwell assays revealed that only 6 µm Cd significantly increased migration and invasion capacities, whereas lower concentrations (1.5 and 3 µm) did not induce these metastasis‐related changes (Figure 1C–F). These findings collectively indicate that 72 h of exposure to 6 µm Cd substantially promotes the proliferation, migration, and invasion of BC cells. To investigate the molecular mechanisms of Cd‐induced BC progression, we conducted an iTRAQ‐based quantitative proteomic analysis comparing T‐47D cells treated with 6 µm Cd with untreated controls. Principal component analysis (PCA) revealed clear separation between the treatment groups (Figure S1A, Supporting Information). Hierarchical clustering analysis revealed 131 differentially expressed proteins (DEPs), comprising 69 upregulated proteins and 62 downregulated proteins (Figure 1G). Gene set enrichment analysis (GSEA) of Gene Ontology (GO) terms revealed significant enrichment of “Regulation of Macroautophagy” (Figure 1H). Complementary KEGG pathway analysis revealed “mitophagy – animal” among the top 10 enriched pathways (enrichment score = 5.54, FDR = 0.032) (Figure 1I); collectively, these results suggest that autophagy‐related pathways may be key mediators of Cd‐induced BC progression. Heatmaps were generated to visualize relative changes in the expression of the autophagy‐associated proteins that were subjected to GSEA and KEGG analyses (Figure S1B, Supporting Information). Autophagy induction is characterized by the conversion of MAP1LC3B‐I to the MAP1LC3B‐II form through lipidation, and the MAP1LC3B‐II/I ratio is commonly used to assess autophagic flux.^[^ ^40^ ^]^ The expression of SQSTM1, an autophagy receptor protein, is inversely correlated with autophagic activity, as its degradation kinetics dynamically indicate autophagic progression.^[^ ^41^ ^]^ TAX1BP1, a multifunctional autophagy receptor that governs phagophore assembly and autophagosome maturation, serves as a key biomarker for assessing the efficacy of selective autophagy.^[^ ^42^, ^43^ ^]^ To delineate the specific effects of Cd exposure on autophagic flux, we systematically measured MAP1LC3B‐II/I levels and SQSTM1 degradation to differentiate between autophagy induction and flux inhibition while simultaneously monitoring TAX1BP1 expression dynamics to investigate potential defects in selective autophagy substrate clearance. Western blot analysis revealed that Cd induced a dose‐dependent accumulation of autophagosomes, as evidenced by alterations in the levels of MAP1LC3B‐II/I, SQSTM1, and TAX1BP1 in T‐47D and MCF‐7 cells, although SQSTM1 levels did not significantly change in Cd‐treated MCF‐7 cells (Figure 1J–O). Chloroquine (CQ), an effective inhibitor of autophagosome and lysosome fusion, blocks autophagy in later stages.^[^ ^44^ ^]^ Notably, when compared to Cd treatment alone, the addition of both CQ and 6 µm Cd did not affect MAP1LC3B‐II levels, suggesting that Cd impedes the clearance of autophagosomes in BC cells (Figure 1P,Q). Monitoring of autophagic flux using an RFP‐GFP‐LC3B kit revealed a significant increase in the number of yellow fluorescent puncta in T‐47D and MCF‐7 cells treated with 6 µm Cd compared with that in control cells, indicating that autophagic clearance is defective in Cd‐treated BC cells (Figure 1R,S). To further assess the contribution of autophagy to Cd‐induced BC progression, Torin1, a highly effective and selective mTOR inhibitor,^[^ ^45^ ^]^ was used to increase autophagic flux. Western blot analyses revealed that Torin1 treatment resulted in increased levels of MAP1LC3B‐II/I and decreased levels of SQSTM1, indicating that Torin1 increased autophagic activity in T‐47D and MCF‐7 cells. Furthermore, Torin1 abrogated the autophagic flux blockade induced by Cd, as demonstrated by the conversion of MAP1LC3B‐I to its membrane‐bound form MAP1LC3B‐II and the clearance of SQSTM1 (Figure S2A–F, Supporting Information). Subsequently, CCK‐8 and Transwell assays revealed that Torin1 reversed the increased proliferation, migration, and invasion of BC cell lines induced by Cd (Figure S2G–L, Supporting Information). Collectively, these findings suggest that impaired autophagy resulting from Cd exposure contributes to the increased proliferation, migration, and invasion of BC cells.

### 2.2 MTA Deficiency Contributes to Cd‐Induced Autophagic Flux Blockade and the Increased Proliferation, Migration, and Invasion of BC Cells

**Figure 2:**
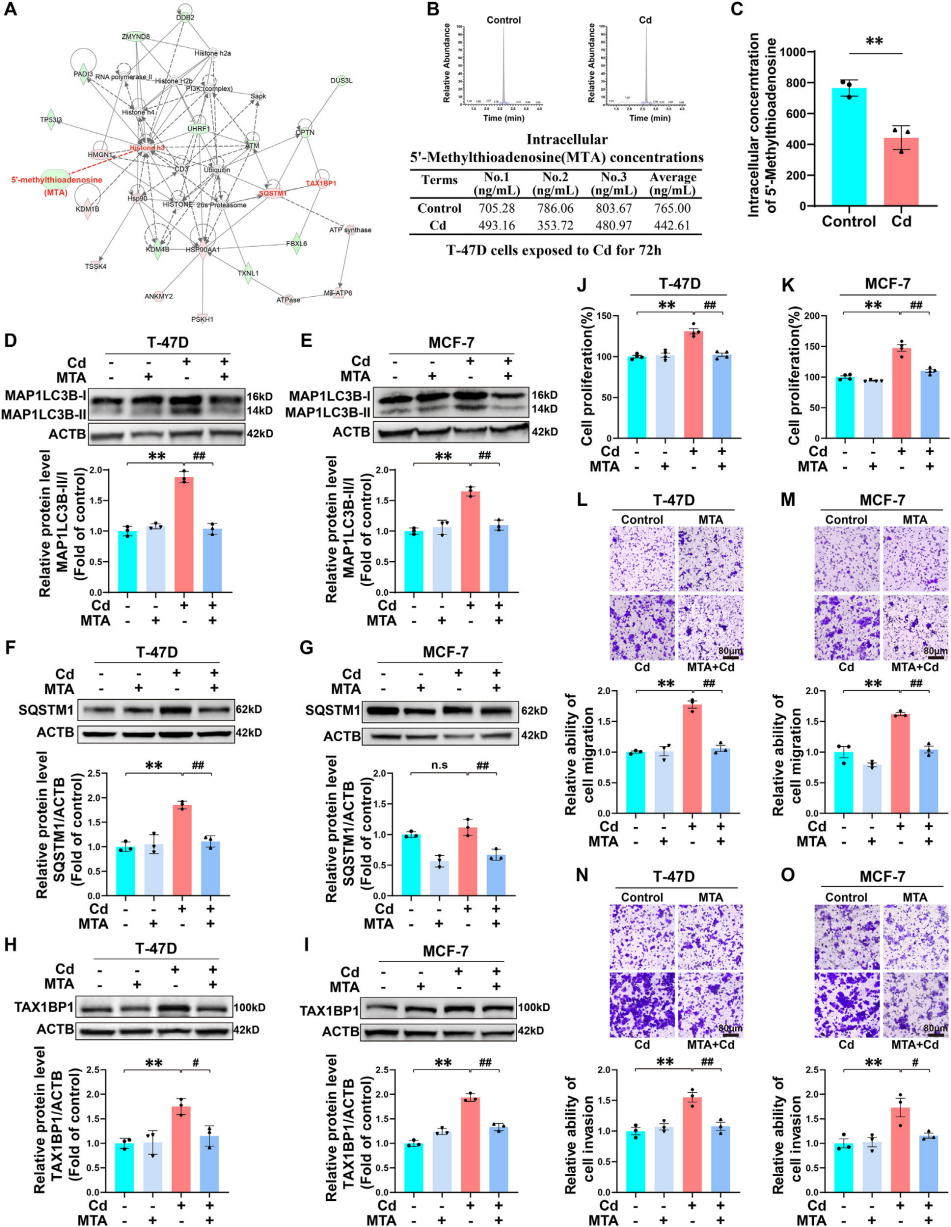
MTA deficiency contributes to Cd‐induced autophagic flux blockade and the proliferation, migration, and invasion of BC cells. In A‐C, all T‐47D cells were treated with or without 6 µm Cd for 72 h, and in D‐O, all T‐47D and MCF‐7 cells were treated with or without 100 µm MTA in the absence or presence of 6 µm Cd for 72 h. A) Predicted crucial network identified by IPA. The network is based on the DEPs and DAMs between the Cd group and the control group. The upregulated molecules are indicated by red shapes, whereas the green shapes correspond to downregulated molecules. B, C) LC‒MS chromatogram and quantification analysis of the intracellular MTA concentration in T47D cells. ^**^ *p* < 0.01 versus the control group. Immunoblotting and quantification of MAP1LC3B D, E), SQSTM1 F, G), and TAX1BP1 H, I) in T‐47D and MCF‐7 cells. J, K) Proliferative activity of T‐47D and MCF‐7 cells. L, M) Migration capacities of T‐47D and MCF‐7 cells. Scale bar: 80 µm. N, O) The invasion capacities of T‐47D and MCF‐7 cells. Scale bar: 80 µm. ^**^ *p* < 0.01 versus the control group; **^#^** *p* < 0.05 and **^##^** *p* < 0.01 versus the Cd group; ns: not significant (*Tukey's HSD*).

Metabolomics serves as a critical layer of biological information that is closest to the phenotype.^[^ ^46^ ^]^ To investigate the molecular mechanisms underlying the Cd‐induced blockade of autophagic flux in BC cells, we conducted metabolomic analyses via gas chromatography‒mass spectrometry (GC‒MS) and liquid chromatography‒mass spectrometry (LC‒MS). The data revealed that Cd significantly altered the metabolic landscape of T‐47D cells, indicating the regulation of metabolic reprogramming. A total of 86 differentially abundant metabolites (DAMs) were detected via GC‒MS, whereas 97 DAMs were detected via LC‒MS (Figures S3 and S4, Supporting Information). To identify the metabolites that play key regulatory roles in Cd‐induced BC progression, we employed an integrated multiomics strategy combining metabolomic and proteomic profiling and constructed a metabolite‒protein interaction network using IPA. The core metabolite MTA has been highlighted for its important relationship with histone H3, as well as its significant interactions with SQSTM1 and TAX1BP1 (**Figure** **2**A). The quantitative results obtained via liquid chromatography‒tandem mass spectrometry (LC‒MS/MS) revealed a significant decrease in the intracellular MTA abundance in the Cd‐exposed T‐47D and MCF‐7 BC cells, further validating the MTA variation trends observed in the IPA network (Figure 2B,C; Figure S5A,B, Supporting Information). MTA is a small molecule recognized for its anticancer properties.^[^ ^29^ ^]^ To evaluate the correlation between MTA levels and clinical characteristics in BC patients, we analyzed tumor tissue samples from 80 patients with luminal‐type BC. The samples were stratified into high‐ and low‐level groups on the basis of the median MTA level. Comparative analyses revealed no significant intergroup differences in age distribution, histological grade, or tumor size. Notably, the low‐MTA group presented a significantly greater incidence of lymph node metastasis and advanced TNM staging than did the high‐MTA group (Table S1, Supporting Information). These data suggest that decreased MTA is strongly associated with increased aggressiveness, metastasis‐related behaviors, and unfavorable clinical staging, supporting its utility as a prognostic biomarker for BC progression. To investigate the role of MTA metabolic reprogramming in BC cells with Cd‐induced impairment of autophagy, we supplemented both the Cd‐treated and untreated T47D and MCF7 cell lines with MTA and monitored changes in the expression of autophagy markers. Notably, MTA supplementation significantly abrogated the Cd‐induced increases in the levels of MAP1LC3B‐II/I, SQSTM1, and TAX1BP1 (Figure 2D‒I). Furthermore, MTA administration markedly inhibited the proliferation, migration, and invasion of Cd‐treated BC cells (Figure 2J‒O). These findings underscore the critical role of MTA deficiency in Cd‐induced autophagic flux blockade, as well as its impact on the proliferation, invasion, and migration of BC cells.

### 2.3 MTA Antagonizes Cd‐Driven H3K79me1 by Decreasing DOT1L Activity in BC Cells

**Figure 3:**
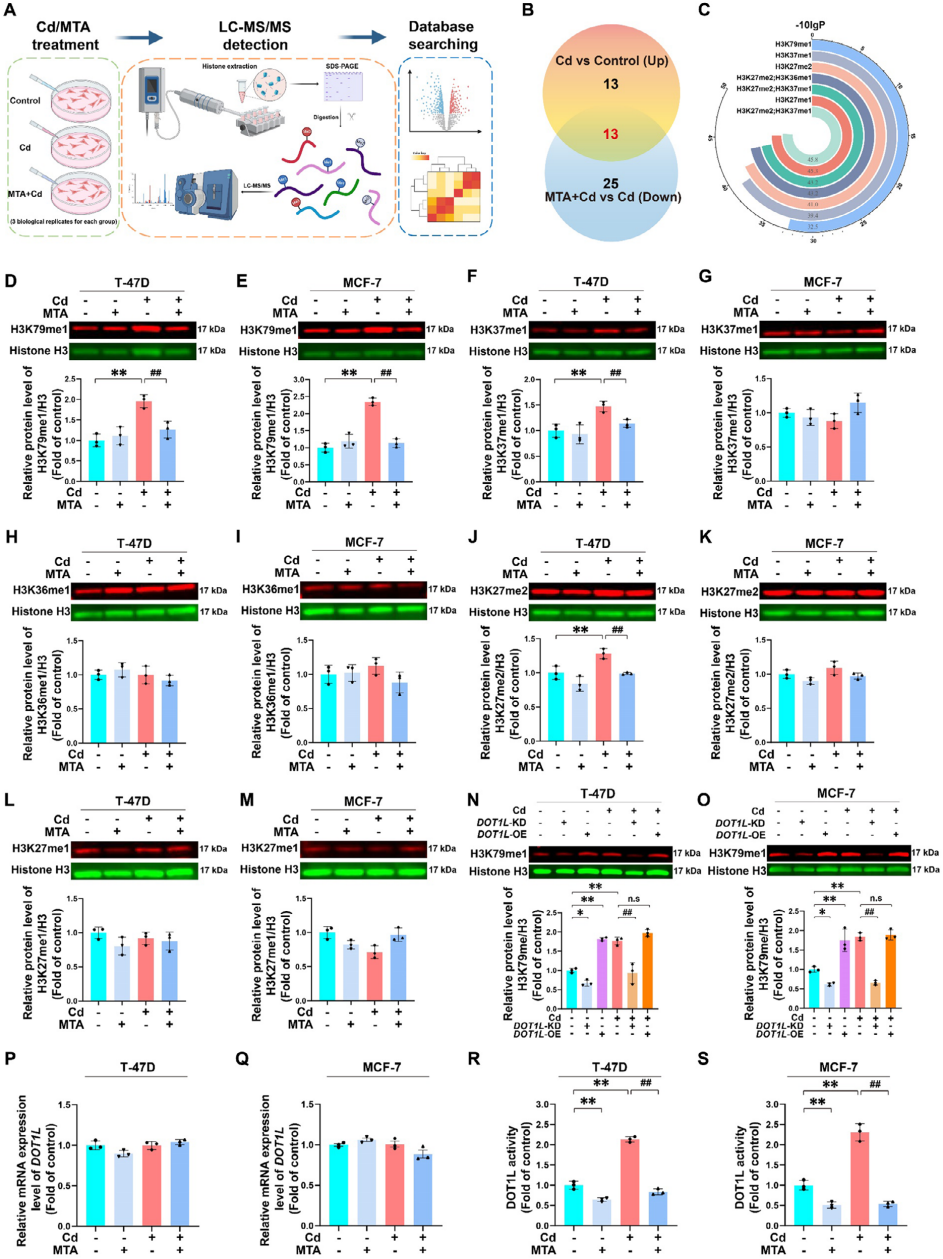
Cd‐induced MTA depletion increased H3K79me1 levels by reactivating DOT1L in BC cells. For A‐M and P‐S, all T‐47D and MCF‐7 cells were treated with or without 100 µm MTA in the absence or presence of 6 µm Cd for 72 h. For N and O, T‐47D and MCF‐7 cells were transfected with the vector, DOT1L‐knockdown or DOT1L‐overexpression plasmid, and treated with or without 6 µm Cd for 72 h. A) Brief schematic of the cell processing and quantitative histone methylation proteomics conducted in T‐47D cells. B) The results of an overlap analysis between histone peptides with increased methylation levels in response to Cd treatment and those with decreased methylation levels induced by the combined treatment of MTA and Cd. C) The identified candidate methylation sites within histone H3. Immunoblotting and quantification of H3K79me1 D, E), H3K37me1 F, G), H3K36me1 H, I), H3K27me2 J, K), and H3K27me1 L, M) in T‐47D and MCF‐7 cells. N, O) Immunoblotting and quantification of H3K79me1 in T‐47D and MCF‐7 cells. P, Q) Relative mRNA levels of *DOT1L* in T‐47D and MCF‐7 cells. R, S) Enzymatic activity of DOT1L in T‐47D and MCF‐7 cells. ^*^ *p* < 0.05 and ^**^ *p* < 0.01 versus the control group; **^##^** *p* < 0.01 versus the Cd group; ns: not significant (*Tukey's HSD*).

To elucidate the interplay between MTA and histone H3, which was included in the IPA network, we extracted histones from T‐47D cells to assess the methylation status of histone tail residues (**Figure** **3**A). LC‒MS/MS detection revealed distinct patterns of histone methylation across the different treatments, identifying histone peptides with differential methylation at lysine and arginine residues (Figure S6A,B, Supporting Information). Overlap analysis identified 13 modified histone peptides with differential methylation that serve as potential targets for Cd and MTA (Figure 3B; Table S2, Supporting Information). Notably, five methylation sites (H3K27me1, H3K27me2, H3K36me1, H3K37me1, and H3K79me1) across seven H3 peptides were characterized (Figure 3C). Dual‐channel near‐infrared fluorescence imaging indicated that Cd treatment caused significant increases in the methylation levels of three sites (H3K27me2, H3K37me1, and H3K79me1), and these effects were inhibited by MTA treatment of T47D cells (Figure 3D,F,H,J, and L). In comparison, in MCF7 cells, only the methylation level of H3K79me1 significantly increased following Cd exposure, and this increase was abolished by MTA treatment (Figure 3E,G,I,K, and M). DOT1L is the only methyltransferase known to catalyze H3K79 methylation.^[^ ^47^ ^]^ To investigate the potential involvement of DOT1L in Cd‐mediated H3K79me1 modification, we generated *DOT1L* knockdown and overexpression cell models. Western blot analysis demonstrated that *DOT1L* overexpression significantly increased H3K79me1 levels, whereas *DOT1L* knockdown markedly reduced Cd‐induced H3K79me1 elevation (Figure 3N,O). Cotreatment with Cd and DOT1L overexpression failed to elicit additive effects on H3K79me1 modification levels, suggesting pathway saturation. Remarkably, DOT1L overexpression in the T‐47D and MCF‐7 cell lines significantly increased the proliferative capacity, migratory potential, and invasive capacity, which are phenotypic alterations that facilitate malignant progression, as observed in Cd‐exposed BC models (Figure S7, Supporting Information). These complementary findings collectively indicate that Cd modulates H3K79me1 in a DOT1L‐dependent manner. Notably, neither Cd exposure nor MTA treatment induced significant alterations in DOT1L mRNA expression levels or protein levels (Figure 3P,Q; Figure S8, Supporting Information). Subsequently, we further assessed its enzymatic activity. Chemiluminescent methyltransferase activity assays revealed that MTA suppressed DOT1L catalytic activity, leading to reduced H3K79me1 modification (Figure 3R,S). Crucially, pharmacological inhibition of DOT1L enzymatic activity markedly reversed Cd‐induced malignant progression in BC cells (Figure S9, Supporting Information), establishing the functional necessity of DOT1L activity in this pathogenic mechanism. Together, these findings demonstrate that MTA mitigates Cd‐driven H3K79me1 elevation and subsequent BC progression through enzymatic inhibition rather than through transcriptional or translational regulation of DOT1L.

### 2.4 *PAK2* is a Downstream Target of Cd‐Induced H3K79me1 in BC Cells

**Figure 4:**
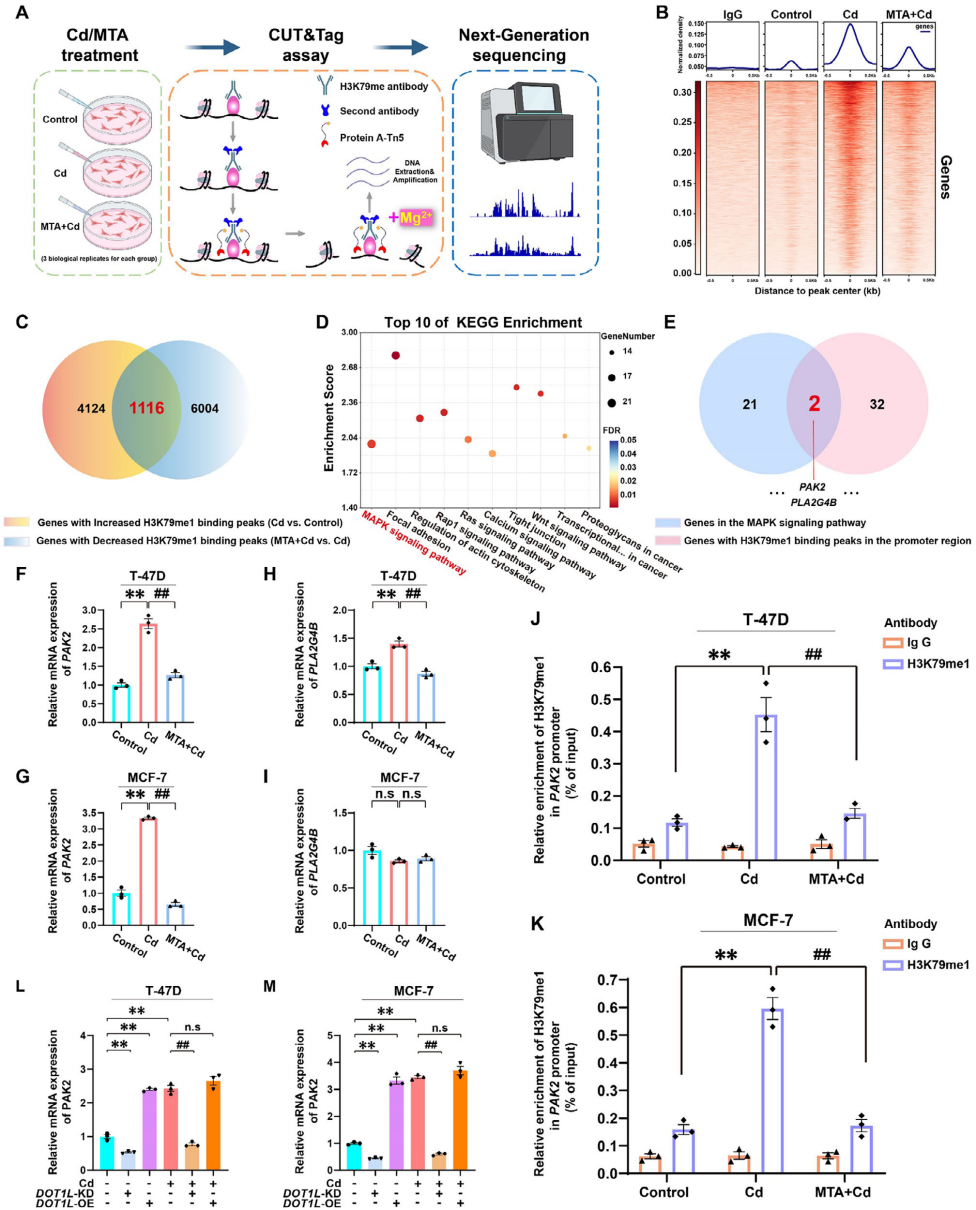
*PAK2* is a downstream target of Cd‐induced H3K79me1 in BC cells. In A‒K, all T‐47D and MCF‐7 cells were treated with or without 100 µm MTA in the absence or presence of 6 µm Cd for 72 h. In L and M, T‐47D and MCF‐7 cells were transfected with the vector, DOT1L‐knockdown or DOT1L‐overexpression plasmid, and treated with or without 6 µm Cd for 72 h. A) Overview of cell processing and CUT&Tag‐seq conducted in T‐47D cells. B) Signal strength and heatmap showing the binding density of H3K79me1 in T‐47D cells. C) The results of an overlap analysis between genes whose H3K79me1 binding peaks increased in response to Cd treatment and those whose H3K79me1 binding peaks decreased in response to the combined treatment with MTA and Cd. D) The top 10 KEGG pathways enriched in the overlapping genes (MAPK signaling pathway: enrichment score = 1.98, FDR = 0.007). E) Overlap analysis identified candidate genes that are enriched in the MAPK signaling pathway and contain H3K79me1 modifications in their promoter regions. F, G) Quantification of *PAK2* expression in T‐47D and MCF‐7 cells. H, I) Quantification of *PLA2G4B* expression in T‐47D and MCF‐7 cells. J, K) ChIP‒qPCR analysis of H3K79me1 enrichment on the promoter of *PAK2* in T‐47D and MCF‐7 cells. L, M) Relative PAK2 mRNA expression in T‐47D and MCF‐7 cells. ^**^ *p* < 0.01 versus the control group; **^##^** *p* < 0.01 versus the Cd group; n.s: not significant (*Tukey's HSD*).

Methylation of histone H3K79 is recognized to be involved in the activation of gene transcription.^[^ ^48^ ^]^ To further clarify the regulatory function of H3K79me1 in Cd‐induced autophagic flux blockade, we performed CUT&Tag analyses in T‐47D cells (**Figure** **4**A). Compared with the control treatment, the Cd treatment significantly increased the enrichment of H3K79me1 binding peaks, whereas MTA treatment effectively restored the levels of these peaks (Figure 4B). Specifically, in comparison with control treatment, Cd treatment increased H3K79me1 binding in 4124 gene regions, whereas combined MTA and Cd treatment reduced H3K79me1 binding in 6004 gene regions. Notably, H3K79me1 binding peaks in 1116 of these genes were regulated by both Cd and MTA (Figure 4C). Additionally, we conducted KEGG pathway analysis on the 1116 genes with specific H3K79me1 binding sites and found that the “MAPK signaling pathway” ranked first (enrichment score = 1.98, FDR = 0.007) (Figure 4D). The overlap analysis revealed two candidate genes, *PAK2* and *PLA2G4B*, which exhibit H3K79me1 binding peaks in their promoter regions (Figure 4E). To identify the precise downstream targets of H3K79me1 regulation, we performed quantitative real‐time PCR (RT‒qPCR) analysis in BC cell lines. Intriguingly, while both *PAK2* expression and *PLA2G4B* expression were affected by Cd and MTA treatment in T‐47D cells, only *PAK2* underwent significant transcriptional regulation under these treatments in MCF‐7 cells (Figure 4F‒I). This differential response across cell lines suggests that *PAK2* may represent a conserved downstream target of H3K79me1 in BC models. Subsequent visualization of the genomic data revealed H3K79me1 occupancy at the *PAK2* promoter, with maximal signal intensity observed in Cd‐treated cells (Figure S10, Supporting Information). To mechanistically validate the Cd‐induced increase in H3K79me1 binding at *PAK2* regulatory regions, we performed quantitative chromatin immunoprecipitation (ChIP)‐qPCR analysis. The results confirmed significant enrichment of H3K79me1 at the *PAK2* promoter following Cd exposure compared with the control in both the T‐47D and MCF‐7 cell lines, an effect reversed by MTA cotreatment (Figure 4J,K). Functional validation through DOT1L manipulation demonstrated that both knockdown and overexpression of this methyltransferase correspondingly altered *PAK2* transcript levels (Figure 4L,M), establishing a direct regulatory relationship between H3K79me1 modification and *PAK2* expression. These converging findings revealed *PAK2* as the downstream target of Cd‐induced H3K79me1 in BC cells.

### 2.5 *PAK2* Overexpression Abolished MTA‐Mediated Inhibition of Cd‐Induced Autophagic Flux Impairment and BC Cell Progression

**Figure 5:**
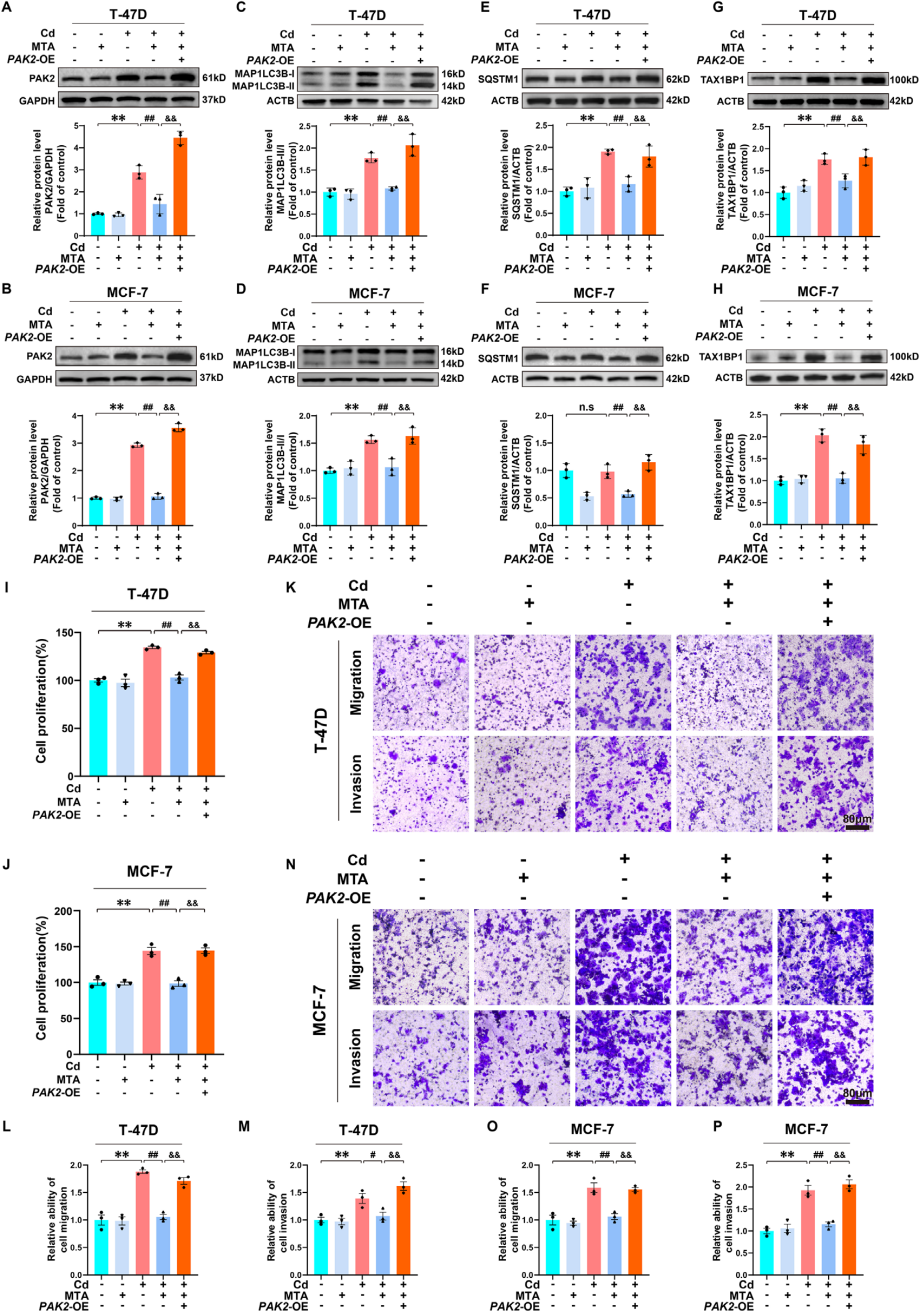
*PAK2* overexpression abolished MTA‐mediated inhibition of Cd‐induced autophagic flux impairment and BC cell progression. In A‐P, all T‐47D and MCF‐7 cells were transfected with the vector or the PAK2‐overexpression plasmid and treated with or without 100 µm MTA in the absence or presence of 6 µm Cd for 72 h. Immunoblotting and quantification of PAK2 A, B), MAP1LC3B C, D), SQSTM1 E, F), and TAX1BP1 G, H) in T‐47D and MCF‐7 cells. I, J) Proliferative activity of T‐47D and MCF‐7 cells. K, L, and M) Migratory and invasive capacities of T‐47D cells. Scale bar: 80 µm. N, O, and P) The migratory and invasive capacities of MCF‐7 cells. Scale bar: 80 µm. ^**^ *p* < 0.01 versus the control group; **^#^** *p* < 0.05 and **^##^** *p* < 0.01 versus the Cd group; ^&&^ *p* < 0.01 versus the MTA+Cd group; n.s: not significant (*Tukey's HSD*).

PAK2 is a serine/threonine kinase that serves as an effector in the downstream signaling of the MAPK pathway.^[^ ^18^ ^]^ We further explored the role of PAK2 in the Cd‐induced blockade of autophagic flux in BC cells. Our data demonstrated that Cd exposure significantly increased PAK2 protein expression levels, whereas MTA treatment effectively suppressed this Cd‐induced upregulation (**Figure** **5**A,B). Notably, overexpression of PAK2 significantly reversed the MTA‐mediated stabilization of autophagic flux, as evidenced by quantitative analysis of established autophagy markers (MAP1LC3B‐II/I, SQSTM1, and TAX1BP1) (Figure 5C‒H). These findings suggest that PAK2 is a pivotal regulator of autophagy dynamics under Cd and MTA cotreatment conditions. Furthermore, functional assays revealed that PAK2 overexpression substantially counteracted the inhibitory effects of MTA on the proliferation, migration, and invasion of Cd‐exposed BC cells (Figure 5I‒P). Complementary pharmacological inhibition experiments demonstrated that targeted suppression of PAK2 activity abolished Cd‐driven malignant progression in BC models (Figure S11, Supporting Information), confirming the essential role of PAK2 in the cancer‐promoting effects of Cd. Collectively, these results establish PAK2 as a downstream mediator of Cd‐induced H3K79me1 modification that orchestrates autophagy blockade and facilitates malignant progression by increasing the proliferative, migratory, and invasive capacities of BC cells.

### 2.6 MTA Supplementation Suppressed the Cd‐Induced Progression and Metastasis of Mammary Tumors by Maintaining H3K79me1 Demethylation‐Mediated Autophagy in MMTV‐ErbB2 Mice

**Figure 6:**
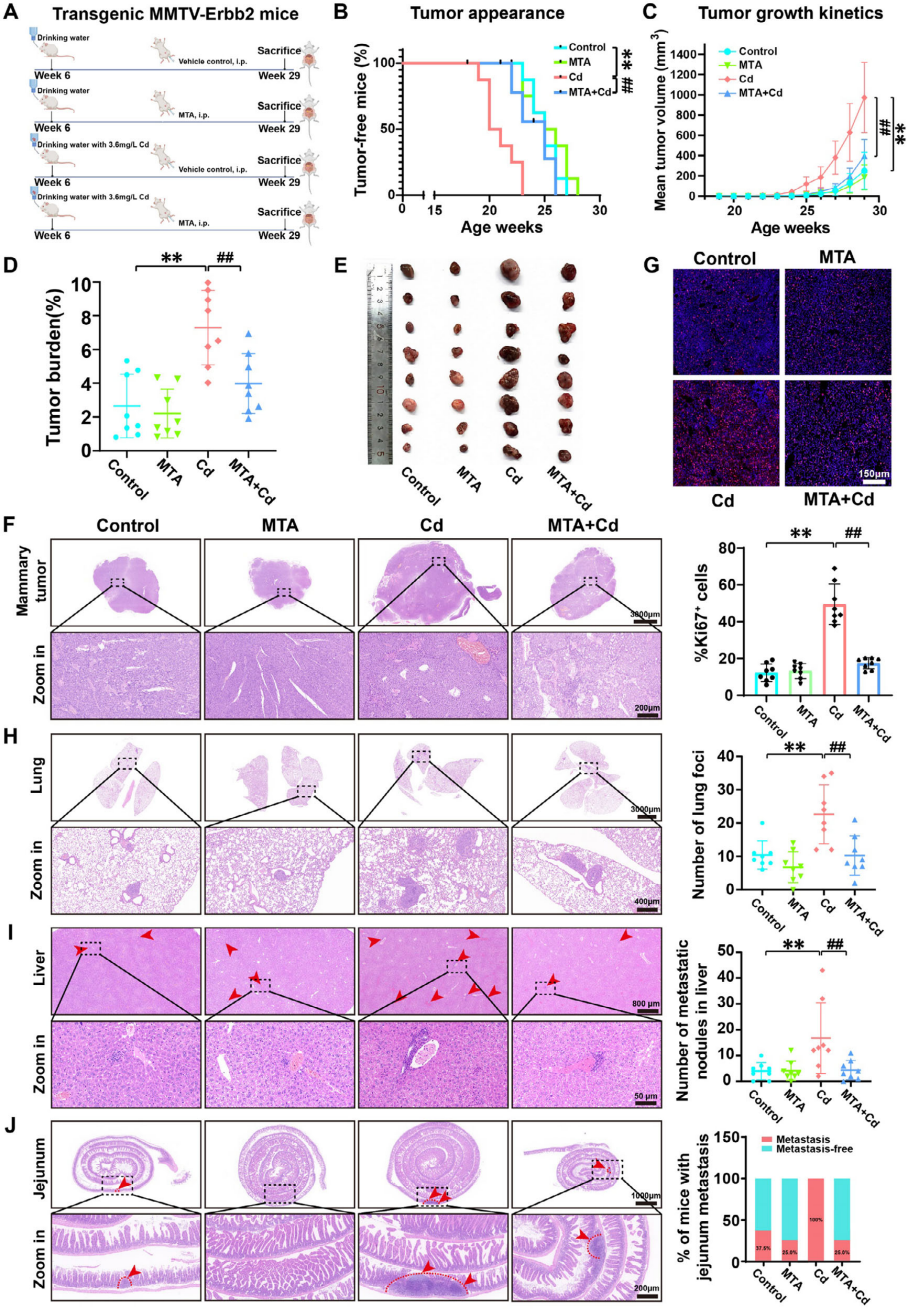
MTA supplementation alleviated the Cd‐induced progression and metastasis of mammary tumors in MMTV‐ErbB2 mice. A) Overview of the experimental design for the MMTV‐ErbB2 mice. Vehicle control, i.p.: Intraperitoneal injection of saline containing 1% dimethyl sulfoxide (DMSO) biweekly; MTA, i.p.: Intraperitoneal injections of 96 µmol kg^−1^ body weight of MTA dissolved in saline containing 1% DMSO biweekly. All MMTV‐ErbB2 mice were exposed to drinking water containing 3.6 mg L^−1^ Cd for a period of 23 weeks. Each group consisted of 8 mice. B) Kaplan‒Meier tumor‐free curves. C) Tumor growth curves showing the average mammary tumor volumes. D) Tumor burden of MMTV‐ErbB2 mice in each group. E) Gross appearances of representative mammary tumors. F) Representative histological images of primary mammary tumor tissue. Scale bars: 3000 µm; zoomed‐in image: 200 µm. G) Representative images of immunofluorescence staining for Ki67 (red) with DAPI (blue), along with quantification of the percentages of Ki67^+^ cells in the mammary tumor tissue. Scale bar: 150 µm. H) Representative histological images of macroscopic metastatic nodules in the lungs (left) and the number of metastatic foci (right). Scale bars: 3000 µm; zoomed‐in image: 400 µm. I) Representative histological images of macroscopic metastatic nodules in the liver (left) and the number of metastatic nodules (right). Scale bars: 800 µm; zoomed‐in image: 50 µm. J) Representative histological images of macroscopic metastatic nodules in the jejunum (left) and the percentage of MMTV‐ErbB2 mice with jejunum metastasis (right). Scale bars: 1000 µm; zoomed‐in image: 200 µm. ^**^ *p* < 0.01 versus the control group; **^##^** *p* < 0.01 versus the Cd group (*Tukey's HSD*).

**Figure 7:**
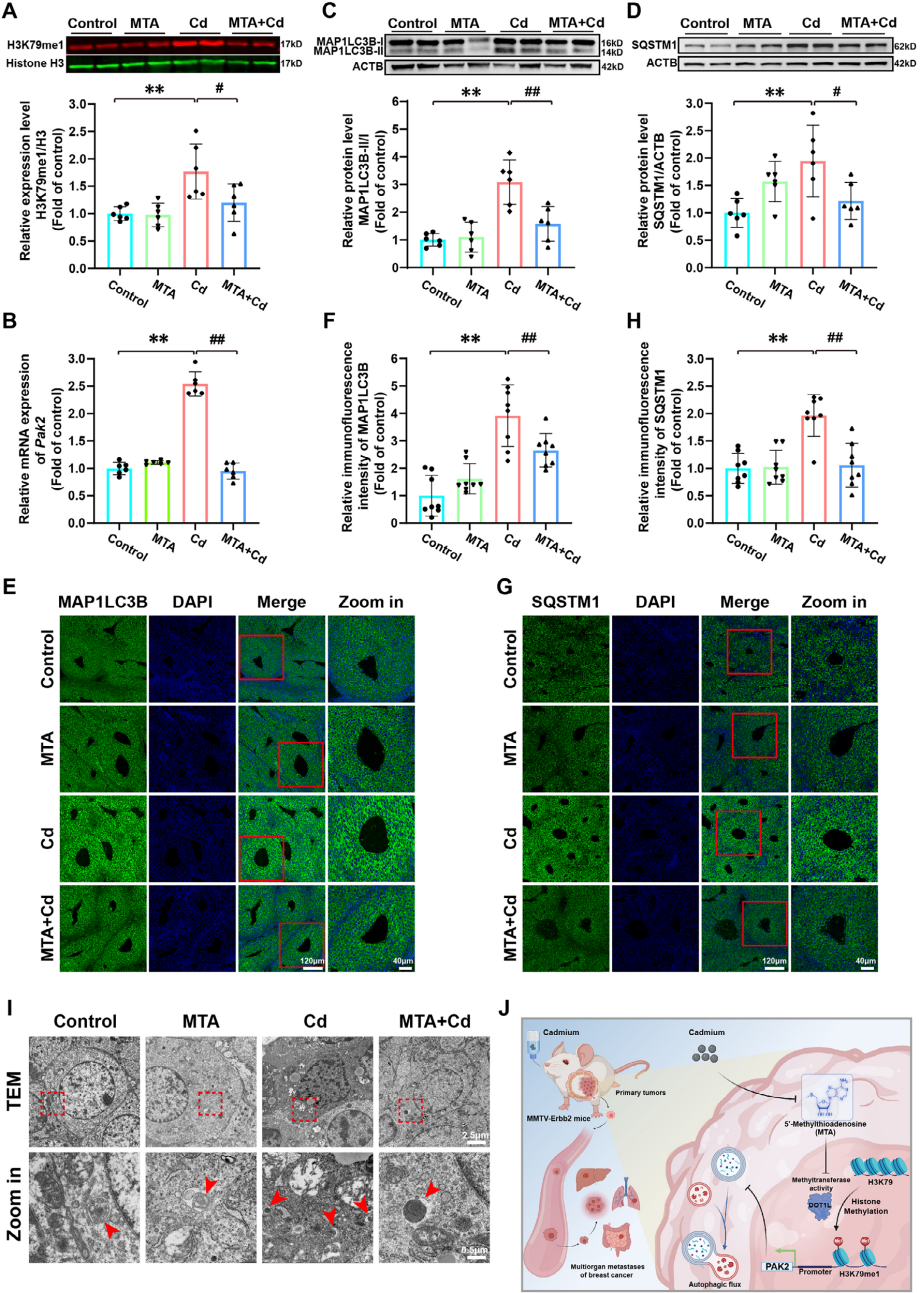
MTA supplementation mitigated Cd‐induced autophagic flux blockade by driving H3K79me1 demethylation and inhibiting Pak2 expression in MMTV‐ErbB2 mice. A) Immunoblotting and quantification of H3K79me1 in mammary tumors. B) Quantification of *Pak2* expression in mammary tumors. C) Immunoblotting and quantification of MAP1LC3B in mammary tumors. D) Immunoblotting and quantification of SQSTM1 in mammary tumors. E) Representative images of MAP1LC3B (green) and DAPI (blue) immunofluorescence staining in mammary tumors. Scale bars: 120 µm; zoomed‐in image: 40 µm. F) Immunofluorescence intensity quantification of MAP1LC3B in mammary tumors. G) Representative images of immunofluorescence staining for SQSTM1 (green) and DAPI (blue) in mammary tumors. Scale bars: 120 µm; zoomed‐in image: 40 µm. H) Immunofluorescence intensity quantification of SQSTM1 in mammary tumors. I) Representative transmission electron microscope images of BC cells in mammary tumors. Scale bars: 2.5 µm; zoomed‐in image: 0.5 µm. J) Schematic diagram showing that MTA metabolic reprogramming drives H3K79me1‐mediated *PAK2* upregulation to promote Cd‐induced BC progression by impairing autophagic flux. ^**^ *p* < 0.01 versus the control group; **^#^** *p* < 0.05 and **^##^** *p* < 0.01 versus the Cd group (*Tukey's HSD*).

MMTV‐ErbB2 mice serve as a clinically relevant model with features closely resembling human pathology.^[^ ^49^ ^]^ These mice spontaneously develop mammary tumors after a prolonged latency period.^[^ ^50^ ^]^ To determine whether the recovery of MTA metabolism antagonized the effect of Cd exposure in vivo, we subjected MMTV‐ErbB2 mice to diverse treatment regimens (**Figure** **6**A). From 18 to 28 weeks of age, we detected palpable mammary tumors and found that the intraperitoneal administration of MTA significantly delayed the early onset of mammary tumors promoted by Cd exposure (median onset: 25 weeks for the control group, 25.5 weeks for the MTA group, 20.5 weeks for the Cd group, and 24 weeks for the MTA+Cd group) and also suppressed the accelerated growth of tumor volume (Figure 6B,C). We subsequently dissected the mice and further evaluated the progression of the mammary tumors. The results indicated that MTA supplementation significantly reduced the tumor size and tumor burden as well as the histopathological grade of the tumors in Cd‐exposed mice (Figure 6D‒F). Immunofluorescence quantification revealed significantly greater Ki67‐positive cell ratios in Cd‐exposed samples than in control samples, and MTA treatment effectively inhibited proliferation activation (Figure 6G). Moreover, MTA injection significantly mitigated Cd‐induced BC multiorgan metastasis to the lungs, liver, and jejunum (Figure 6H‒J). We then collected mammary tumor tissues to investigate the in vivo changes in H3K79me1 levels. Compared with control treatment, Cd exposure led to significant increases in H3K79me1 levels and *Pak2* transcript levels in mammary tumor tissues. As expected, the addition of MTA significantly reduced both the H3K79me1 and *Pak2* levels (**Figure** **7**A,B). Consistent with the results of our previous in vitro experiments, exposure to Cd led to significant accumulation of MAP1LC3B and SQSTM1 in mouse BC cells, which was notably alleviated by supplementation with MTA (Figure 7C‒H). Moreover, transmission electron microscopy revealed that Cd exposure led to the subcellular accumulation of autophagosomes, an effect that was mitigated by MTA treatment (Figure 7I). Therefore, our findings shed light on the roles of MTA metabolic reprogramming in Cd‐mediated autophagic flux impairment and BC progression in vivo.

## 3 Discussion

Cd, a well‐documented environmental carcinogen, predominantly enters human systems through contaminated water and food chains and greatly increases cancer risk.^[^ ^51^ ^]^ Clinical investigations have revealed significant increases in peak blood Cd concentrations in BC patients (3.15 µg L^−1^, 28.02 nm) compared with healthy controls (2.57 µg L^−1^, 22.86 nm).^[^ ^52^ ^]^ Notably, a dose‒dependent relationship emerged in BC progression, with nonmetastatic patients showing maximum blood Cd levels of 26.96 nm compared with 35.23 nm in those with distant metastases.^[^ ^10^ ^]^ These clinical observations establish Cd exposure as a plausible etiological factor in BC pathogenesis. Importantly, chronic exposure leads to preferential Cd deposition in target organs, as evidenced by aortic tissue concentrations reaching 7 µm in young smokers, suggesting that localized tissue levels may substantially surpass those detected in blood samples.^[^ ^53^ ^]^ In this study, we investigated the procancer effects of 0, 1.5, 3, and 6 µm Cd, a concentration range that may closely reflect bioavailable Cd levels in tissue microenvironments, in accordance with established in vitro models of mammary carcinogenesis.^[^ ^54^, ^55^, ^56^ ^]^ To simulate the in vivo effects of chronic Cd exposure at environmentally relevant doses, we administered drinking water containing 3.6 mg L^−1^ (14.4–25.2 µg day^−1^) Cd to MMTV‐ErbB2 mice for 23 weeks, in accordance with our previous studies.^[^ ^11^, ^12^ ^]^ This concentration reflects the Cd levels found in contaminated surface water and groundwater (0.025– 6.26 mg L^−1^) and aligns with the estimated dietary Cd intake of females at a high risk of BC (16.1 µg d^−1^), as reported in an Italian cohort study.^[^ ^18^, ^57^, ^58^, ^59^ ^]^ Furthermore, evidence indicates that mice given drinking water containing 3 mg L^−1^ Cd for 20 weeks presented mean blood Cd levels comparable to those observed in American female smokers (0.69–1.17 µg L^−1^).^[^ ^60^ ^]^ Thus, the dose of Cd in our study is representative of population‐level Cd exposure, providing valuable insights into the correlation between environmental Cd exposure and BC occurrence while elucidating the underlying mechanisms involved.

Autophagy, a vital cellular degradation pathway that maintains metabolic balance and homeostasis, is implicated in the pathogenesis of various types of cancer.^[^ ^61^, ^62^ ^]^ Recent studies suggest that autophagy significantly influences the toxicological effects of heavy metals, including Cd, copper, and arsenic, which are prevalent among environmental pollutants.^[^ ^63^, ^64^ ^]^ Cd is particularly recognized for its ability to induce autophagy dysregulation through various pathways, thereby enhancing its procancer effects. Research has shown that chronic exposure to Cd facilitates the formation of the NOX1 complex, leading to autophagic dysregulation, which contributes to the malignant transformation of prostate epithelial cells.^[^ ^65^ ^]^ Moreover, Cd promotes cancer progression by activating K63‐linked ubiquitination and NF‐κB signaling while simultaneously disrupting selective autophagy.^[^ ^66^ ^]^ Our previous investigations indicated that Cd mediates the proliferation, migration, and invasion of BC cells by inhibiting ATG5‐dependent autophagosome formation and modulating ATG5 expression to maintain autophagic flux, thus mitigating Cd‐induced malignancy.^[^ ^18^ ^]^ On the basis of these findings, we examined the characteristics of autophagy in cells treated within a narrower concentration range of Cd in this study; we confirmed the conclusion that autophagy dysregulation is a common feature of BC progression and that the induction of autophagy may offer protective effects against heavy metal toxicity. This research provides more detailed evidence of the relationship between environmental factors and autophagy dysregulation in tumor progression.

Cancer cells exhibit distinct metabolic characteristics during the process of malignant progression.^[^ ^67^, ^68^ ^]^ Emerging evidence indicates that metabolic changes support the rapid growth and dissemination of BC cells. For example, fatty acid oxidation modulates acetyl‐CoA‐related acetylation of H3K9 and H3K27 in triple‐negative breast cancer (TNBC), driving TNBC metastasis.^[^ ^69^ ^]^ Moreover, dysregulation of propionate metabolism results in the accumulation of methylmalonic acid, which generates a signature in BC cells that increases their aggressiveness and metastatic potential.^[^ ^70^ ^]^ In this study, we found that MTA, an intermediate in the methionine cycle,^[^ ^71^ ^]^ exerts anti‐BC effects via autophagy modulation. Autophagy is precisely regulated in response to metabolic changes, ultimately influencing tumor growth, metastasis, and treatment responses. Evidence suggests that prolonged glutamine starvation reactivates mTOR, which suppresses autophagy to sustain the viability of oral cancer cells.^[^ ^72^ ^]^ Consistent with this report, we demonstrated that Cd‐driven MTA metabolic reprogramming facilitates the malignant progression of BC cells by inducing autophagic flux blockade both in vivo and in vitro. As an environmental estrogen mimetic, Cd has been established to exert carcinogenic effects through estrogenic activity across multiple cancer types.^[^ ^8^ ^]^ Emerging evidence has demonstrated that estrogen signaling critically regulates colorectal cancer progression through ERα‐mediated modulation of the purine synthesis pathway.^[^ ^73^ ^]^ Given the central role of MTA in purine metabolism, we propose that ERα‐dependent estrogen signaling mediates Cd‐induced perturbations in MTA metabolic homeostasis. Notably, consistent with prior studies,^[^ ^74^, ^75^ ^]^ we further observed that Cd exposure (1.5, 3, and 6 µm, 72 h) failed to induce a malignant phenotype in the TNBC cell lines MDA‐MB‐231 and MDA‐MB‐468; these effects contrast those detected in T‐47D and MCF‐7 cells (Figure S12, Supporting Information). This subtype‐specific differential response provides evidence suggesting that ERα signaling may serve as a principal mediator of Cd‐induced MTA metabolic reprogramming while underscoring the need for targeted mechanistic investigations to delineate the regulatory role of estrogen signaling in coordinating Cd‐induced metabolic dysregulation.

Distinct methylation patterns regulate the activation (e.g., H3K4, K36, and K79) or silencing (e.g., H3K9, K27, and H4K20) of specific genes, critically regulating cell fate.^[^ ^76^, ^77^ ^]^ As a methyltransferase inhibitor, MTA modulates transcriptional programs through site‐specific histone methylation control.^[^ ^18^ ^]^ In LPS‐induced inflammatory macrophages, MTA suppresses H3K4me2 and H3K4me3 to inhibit the expression and release of IL‐6 and TNF‐α.^[^ ^78^ ^]^ Additionally, MTA inhibits H3K4me3 and H3K36me3 at gene promoters, resulting in a decrease in thymic stromal lymphopoietin‐induced PINK1 and LC3 accumulation in human monocytes.^[^ ^79^ ^]^ In the present study, we identified H3K79me1 as a regulatory site for MTA using innovative histone methylation proteomics. Increasing evidence suggests that aberrant H3K79 methylation contributes to enhanced progression potential in various types of cancer, including breast, colon, and ovarian cancer.^[^ ^80^, ^81^, ^82^ ^]^ In this study, we observed that Cd increased H3K79me1 levels and promoted BC progression by disrupting autophagic flux. Moreover, the methyltransferase DOT1L is essential for H3K79 methylation.^[^ ^83^ ^]^ It has been shown to mediate H3K79me3 dysregulation on the MTDH Wt/Δ7 promoter, thereby increasing transcription in TNBC; depletion of DOT1L abrogates this effect.^[^ ^84^ ^]^ Our findings consistently indicate that MTA‐driven demethylation of H3K79me1 relies on the inhibition of DOT1L activity, thereby mitigating Cd‐driven BC progression. Recent reports suggest that specific inhibitors that target H3K79 methylation and DOT1L are currently under development for the treatment of various types of cancer.^[^ ^85^ ^]^ Given the formidable challenge of drug resistance in BC, researchers have continuously explored novel therapeutic targets and strategies. Emerging DOT1L inhibitors show therapeutic potential, particularly in overcoming endocrine resistance through synergy with tamoxifen/fulvestrant and reversing multidrug resistance via cancer stem cell modulation.^[^ ^36^, ^86^ ^]^ Metabolic reprogramming, recognized as a pivotal hallmark of tumor progression, has also gained increasing attention for its role in therapy resistance.^[^ ^87^ ^]^ Our study provides compelling in vitro and in vivo evidence that MTA effectively counteracts Cd‐induced BC progression through methyltransferase activity inhibition and that its level is significantly inversely correlated with the extent of lymph node metastasis and the TNM stage in BC patients. These data substantiate that MTA‐mediated metabolic reprogramming is a central pathogenic mechanism in oncogenesis rather than a mere epiphenomenon. If we expand our findings, targeting MTA metabolic reprogramming and DOT1L inhibition could be a promising therapeutic strategy for combating BC metastasis.

PAK2 is the sole universally expressed member of the serine/threonine kinase P21‐activated kinase family,^[^ ^18^ ^]^ and its elevated expression is associated with advanced clinical stages of ovarian and pancreatic cancers.^[^ ^88^, ^89^ ^]^ In addition, PAK2 has been proposed as an independent prognostic marker for gastric cancer.^[^ ^90^ ^]^ The expression of *PAK2* in tumor cells is regulated by various endogenous upstream regulators. In human osteosarcoma, paired box gene 3 upregulates *PAK2* expression and promotes the migration and differentiation of tumor cells by binding to the promoter region of the *PAK2* gene.^[^ ^91^ ^]^ Similarly, long‐chain fatty acyl‐CoA ligase 4 facilitates the binding of Sp1 to the *PAK2* promoter, thereby increasing *PAK2* transcription, which contributes to malignant progression in hepatocellular carcinoma.^[^ ^92^ ^]^ Here, we demonstrate that MTA metabolic dysregulation leads to a reduction in H3K79me1 levels on the *PAK2* promoter, which is implicated in the progression of Cd‐exposed BC. Recently, a study showed that the upregulation of *PAK2* expression increased neuroblastoma cell proliferation, migration, and invasion by inhibiting autophagic flux.^[^ ^93^ ^]^ Our findings are consistent with this inference and confirm the role of PAK2 in the Cd‐induced impairment of autophagic flux. However, a recent study demonstrated that a PAK2 inhibitor suppresses the migration and metastasis of TNBC cells through the inhibition of autophagy.^[^ ^94^ ^]^ These conflicting results suggest that the effects of PAK2‐regulated autophagy markedly differ across various molecular subtypes of BC, further underscoring the heterogeneity of tumors.

Several limitations must be acknowledged in this study. First, autophagy is not the only pathway altered in the process of Cd‐induced BC progression. The results of the proteomic analysis indicate that numerous biological processes related to BC warrant further investigation. Moreover, the Cd‐induced metabolic‐epigenetic axis identified here requires validation in additional BC cell lines to strengthen the generalizability and robustness of these conclusions. Additionally, the impact of Cd on tumors in real scenarios is complex and influenced by multiple confounding factors, so biases may have been introduced in the study of the relationship between human exposure to a single pollutant and tumor progression.

In this study, we first demonstrated that i) MTA metabolic reprogramming plays a core regulatory role in the epigenetic‐autophagy axis in the context of Cd‐induced BC malignancy; ii) Cd‐induced MTA depletion specifically decreases DOT1L methyltransferase activity and increases H3K79me1 levels in the *PAK2* promoter region, promoting the expression of *PAK2*, which contributes to the autophagic flux blockade required for BC progression; and iii) MTA levels negatively correlate with BC tumor stage, thereby substantiating the clinical importance of MTA depletion in BC progression. Taken together, the reported data provide a better understanding of the metabolic and epigenetic regulation of Cd‐mediated BC progression and offer a novel perspective for the comprehensive assessment of the ecological and health risks associated with Cd exposure.

## 4 Experimental Section

### Cell Culture and Cd Treatment

Human BC cell lines (T‐47D and MCF‐7 cells) were obtained from Procell Life Science and Technology Co., Ltd. (Wuhan, China), and TNBC cell lines (MDA‐MB‐231 and MDA‐MB‐468 cells) were obtained from Hysigen Bioscience. After being cultured in DMEM (Gibco, NY, USA; C11995500BT) supplemented with 10% fetal bovine serum (AUSGENEX, Molendinar, Australia; FBS500‐S) and 1% (v/v) penicillin/streptomycin (Gibco, NY, USA; 15140‐122) at 37 °C in a humidified atmosphere with 5% CO_2_,^[^ ^95^ ^]^ all the cell lines were treated with 0, 1.5, 3, or 6 µm Cd for 72 h. A stock solution of cadmium chloride (Sigma‒Aldrich, St. Louis, MO, USA; 439800) was prepared using distilled deionized water and diluted with medium accordingly. In addition, 25 µm CQ (MCE, New Jersey, USA; HY‐17589A), 100 nm Torin 1 (MCE, New Jersey, USA; HY‐13003), 100 µm MTA (MCE, New Jersey, USA; HY‐16938), 50 µM EPZ004777 hydrochloride (MCE, New Jersey, USA; HY‐15227A), and 2 µm FRAX486 (MCE, New Jersey, USA; HY‐15542B) were used in line with previous research.^[^ ^18^, ^84^, ^94^, ^96^, ^97^, ^98^ ^]^

### Cell Proliferation Assays

T‐47D cells (8 × 10^3^ cells per well), MCF‐7 cells (5 × 10^3^ cells per well), MDA‐MB‐231 cells (5 × 10^3^ cells per well), and MDA‐MB‐468 cells (8 × 10^3^ cells per well) were seeded in 96‐well plates and incubated overnight. Then, the cells were exposed to the appropriate treatment for 72 h. A CCK‐8 assay kit (Dojindo, Kyushu, Japan, CK04) was used to evaluate the proliferative ability of the cells. The absorbance readings were taken at a wavelength of 450 nm using the Infinite 200 PRO Enzyme Labeling System (Infinite M200, Tecan, Austria).

### Cell Migration and Invasion Assays

The migratory and invasive properties of BC cells were evaluated via Transwell assays. The cells were resuspended in serum‐free medium at specific concentrations (5 × 10^5^ cells mL^−1^ for T‐47D cells, 2.5 × 10^5^ cells mL^−1^ for MCF‐7 cells, 1 × 10^5^ cells mL^−1^ for MDA‐MB‐231 cells and 5 × 10^5^ cells mL^−1^ for MDA‐MB‐468 cells) prior to being seeded in the upper chambers of 24‐well Transwell plates (Corning, New York, USA; 3422). The lower chamber was replenished with 800 µL of complete medium loaded with the assigned treatment. For the invasion assays, the upper chamber was coated with Corning BD Matrigel (Corning, New York, USA; 356 234), whereas the migration assays were performed without Matrigel. After a 72‐h incubation period, the cells on the upper surface of the membrane were carefully removed with a cotton swab. The cells that had migrated to or invaded through the membrane were fixed with a 4% paraformaldehyde solution for 15 min and then stained with a 0.5% Crystal Purple Methanol Solution (Solarbio, Beijing, China; G1072) for 10 min. The stained cells were subsequently examined under an inverted microscope, and the number of cells that had migrated or invaded was subsequently quantified.

### iTRAQ‐Labeling Quantitative Proteomics Analysis

T‐47D cells (3 × 10⁶) were cultured in 10 cm dishes and exposed to 0 µm (control) or 6 µm Cd for 72 h (triplicates per group). Following lysis of 1 × 10⁷ cells sample^−1^ with SDT buffer, the protein concentration was determined via a BCA assay. Aliquots (50 µg) underwent sequential reduction (5 mm DTT, 55 °C, 30 min), alkylation (10 mm iodoacetamide, dark, 15 min), acetone precipitation, and reconstitution in 100 mm TEAB. Trypsin‐TPCK digestion (1:50 w/w) was performed overnight at 37 °C. Lyophilized peptides were labeled with iTRAQ reagents (isopropanol‐activated) for 2 h at RT, quenched with ultrapure water, and stored at −80 °C.^[^ ^99^ ^]^ Fractionation was performed with an Agilent 1100 HPLC system with a Zorbax Extend‐C18 column (2.1 × 150 mm, 5 µm) with a mobile phase pH of 10.0 for the following solutions: A) 2% acetonitrile and B) 90% acetonitrile. Gradient elution (300 µL min^−1^) was performed, and 15 fractions were collected between 8 and 60 min. For LC‐MS/MS analysis, samples were loaded onto Evotip‐coupled 15 cm columns with mobile phases containing 0.1% formic acid. The MS acquisition parameters included the following: 1.5 kV capillary voltage, 180 °C drying gas, 100–1700 m z^−1^ scan range, and 20–59 eV collision energy. MaxQuant 1.6.17.0 processed raw data against reference databases using thresholds of ≥ 1 unique peptide and a ≥50% protein identification rate. Missing values (≤ 50% per group) were replaced by group means. Statistical significance was determined via Benjamini‒Hochberg adjusted p values (FDR), with DEPs defined as those with a |fold change| > 1.5 and an FDR < 0.05. The iTRAQ‐labeling quantitative proteomics analysis was conducted by OE Biotechnology Co., Ltd. (Shanghai, China). The mass spectrometry proteomics data have been deposited in the BIG submission portal with the dataset identifier PRJCA033458.

### Western Blotting

Following treatment, the cells or tissues were collected and lysed with RIPA buffer (Beyotime, Shanghai, China; P0013B) supplemented with protease inhibitor cocktails (Roche, Indianapolis, IN, USA; 0 469 312 4001) and phosphatase inhibitors (Roche, Indianapolis, IN, USA; 0 490 683 7001) for subsequent Western blot analysis. SDS‒PAGE‐separated proteins were transferred to polyvinylidene fluoride (PVDF) membranes (Bio‐Rad, CA, USA; 162–0177), and the ChemiDoc XRS+System (Bio‐Rad, Hercules, CA, USA) facilitated blot visualization and quantification. Additionally, proteins on histone methylation detection blots were transferred to low‐fluorescence PVDF (PVDF‐LF) membranes (Bio‐Rad, CA, USA; 162–0264), and rabbit or mouse fluorescent secondary antibodies were used to assess signal intensity with an Odyssey Infrared Imaging System 301 (LI‐COR, Lincoln, NV, USA). A comprehensive list of the antibodies utilized in this study is presented in Table S3 (Supporting Information).

### Autophagic Flux Monitoring

T‐47D cells and MCF‐7 cells were seeded in confocal dishes and treated overnight with the Premo Autophagy Tandem Sensor RFP‐GFP‐LC3B Kit (Invitrogen, Carlsbad, CA, USA; P36239). The T‐47D cells were then treated with 6 µm Cd for an additional 72 h. All the samples were observed under a Zeiss LSM 800 confocal laser scanning microscope (Carl Zeiss, Jena, Germany) equipped with a 63 × oil immersion objective.

### Untargeted Metabolomics Profiling

LC‒MS and GC‒MS were employed for a comprehensive analysis of metabolites in the cellular samples. The sample preparation adhered to the protocol previously published.^[^ ^100^ ^]^ For LC‒MS analysis, a precooled methanol‒water mixture was used for the extraction of cellular metabolites, and mass spectrometric information was subsequently acquired using a Nexera UPLC coupled with a Q Exactive high‐resolution mass spectrometer. GC‒MS analysis was conducted on a 7890B‐5977A GC‒MS system (Agilent Technologies, Santa Clara, CA, USA) equipped with a DB‒5MS column, employing a temperature gradient and electron ionization for metabolite separation and detection. Data processing was performed using Progenesis QI v2.3 software, and compound identification and metabolic pathway analysis were facilitated by integration with multiple databases. Principal component analysis and partial least squares‐discriminant analysis were employed to assess sample distribution and analytical stability, with DAMs selected on the basis of VIP scores and p values. Statistical significance was determined via Benjamini‒Hochberg adjusted *p* values (FDR), with DAMs defined as those with a |fold change| > 2, FDR < 0.05, and variable importance in projection (VIP) > 1. The analysis was carried out by OE Biotechnology Co., Ltd. The metabolomics data have been deposited in the BIG submission portal with the dataset identifier PRJCA033456.

### Quantitative Determination of MTA by LC–MS

LC–MS was employed for the quantification of MTA in T‐47D and MCF‐7 cell samples by Servicebio Technology (Wuhan, Hubei, China). Chromatographic separation was accomplished using a Welch XB‐C8 column (150 × 4.6 mm, 5 µm) (Thermo Fisher Scientific, San Jose, CA, USA) with a mobile phase composed of 0.1% aqueous formic acid (aqueous phase) and methanol (organic phase) at a flow rate of 1.0 mL min^−1^. The column temperature was maintained at 40 °C, while the autosampler was set at 10.0 °C with an injection volume of 5.00 µL. For mass spectrometric detection, a TSQ Quantum triple quadrupole mass spectrometer equipped with a positive ion mode electrospray ionization source was operated under selected reaction monitoring conditions. The ESI voltage was set at + 3500 V, with a capillary temperature of 400 °C. Nitrogen gas (purity ≥ 99.999%) was used to maintain the sheath gas pressure at 60 Arb, while the auxiliary gas pressure was set at 20 Arb with the same grade of nitrogen. Argon gas (purity ≥ 99.999%) served as the collision gas. Data acquisition was carried out over a period of 5.00 min. The mass spectrometry method was optimized for the analysis of MTA. Calibration standards were prepared by dissolving an appropriate amount of the standard compound in 0.1% methanolic formic acid to create a 2.00 mg mL^−1^ stock solution, which was further diluted to a working concentration of 2 µg mL^−1^. Sample preparation involved the precise extraction of 100 µL of sample, followed by the addition of 300 µL of pure methanol and vortex mixing with zirconia beads for 5 min. After centrifugation at 13 000 rpm for 10 min at 4 °C, the supernatant was collected for analysis. Chromatographic data acquisition and integration for methionine adenosine were processed and integrated using Xcalibur 3.0 software (Thermo), which employs weighted coefficients for linear regression analysis to ensure accurate quantification.

### Quantitative Histone Methylation Proteomics via LC‒MS/MS

The analysis was performed as previously described.^[^ ^101^ ^]^ Specifically, T‐47D cell samples were collected, followed by the addition of hypotonic lysis buffer and ultrasonic disruption. Histones were isolated and enriched through trichloroacetic acid precipitation. The samples were subsequently loaded onto a 12% separation gel and subjected to SDS‒PAGE for protein separation. The target band was excised into 1 mm^3^ gel pieces, which were then digested with trypsin. An extraction solution consisting of 5% trifluoroacetic acid, 50% acetonitrile, and 45% water was prepared to extract the peptides. Desalting was performed using a self‐packed desalting column, followed by solvent evaporation in a vacuum centrifugal concentrator at 45 °C to prepare for subsequent mass spectrometry analysis. Finally, the peptides were analyzed via LC‒MS/MS with an Acclaim PepMap RPLC C18 column (3 µm, 100 Å, 150 µm inner diameter × 150 mm). The mobile phase consisted of water containing 0.1% formic acid and an 80% ACN solution, with a flow rate of 300 nL min^−1^ and an analysis duration of 120 min. PEAKS Studio software was used to search target protein databases and identify variable modifications. Monomethylation of lysine (K)/arginine (R)(+14.02 Da), dimethylation of K/R(+28.03 Da), and trimethylation of K(+42.05 Da) were specified as modifications. Statistical significance was determined via Benjamini‒Hochberg adjusted p values (FDR) < 0.05 and |fold change| > 2. A reference database containing only human (*Homo sapiens*) histone sequences was utilized. LC‒MS/MS was performed by BiotechPack Technology Company, Ltd. (Beijing, China), and the acquisition data were deposited in the BIG submission portal with the dataset identifier PRJCA033478.

### RT–qPCR

Total RNA was isolated with TRIzol reagent (Thermo Fisher Scientific, MA, USA; 15596026CN). cDNA was synthesized from 2 µg of RNA using a PrimeScript RT Reagent Kit (Takara, Shiga, Japan, RR047A) in accordance with the manufacturer's instructions. The quantification of all gene transcripts was conducted via RT‒qPCR with SYBR Green Master Mix (Takara, Shiga, Japan, RR820A). The specific primers utilized are detailed in Table S4 (Supporting Information). The results were analyzed using the 2^−ΔΔCT^ method.

### DOT1L Enzyme Activity Assay

The enzymatic activity of DOT1L was assessed via the use of 5 µm S‐adenosyl methionine as the methyl group donor, along with a synthesized DOT1L substrate. The reaction involved 25 ng µL^−1^ DOT1L enzyme obtained from BPS Bioscience (San Diego, CA, USA; 52 202), in accordance with the manufacturer's guidelines.^[^ ^37^ ^]^

### Plasmid Construction and Transfection

For *DOT1L* overexpression and knockdown, the plasmids pcDNA3.1‐CMV‐H_DOT1L‐3×Flag‐EF1‐ZsGreen‐T2A‐Puro and short hairpin RNA‐*DOT1L* were provided by Hanheng Biotechnology (Shanghai) Co., Ltd. For *PAK2* overexpression, full‐length *PAK2* was cloned and inserted into the PGMLV‐CMV‐MCS‐3×Flag‐EF1‐ZsGreen1‐T2A‐Puro vector by Genomeditech (Shanghai) Co., Ltd. All expression constructs were verified through Sanger sequencing. The plasmids were transfected into T‐47D and MCF‐7 cells for 24 h using the X‐tremeGENE HP DNA Transfection Reagent (Roche, Indianapolis, IN, USA; 0 636 623 6001), with the empty vector serving as a negative control.

### ChIP and ChIP‒qPCR

ChIP was performed using the ChIP‐IT High Sensitivity Kit (Active Motif, Carlsbad, CA, USA; 5030) according to the manufacturer's instructions. A total of 2 × 10^7^ cells were subjected to crosslinking with 1% formaldehyde at room temperature for 10 min. Crosslinking was terminated by the addition of glycine to achieve a final concentration of 0.125 m. These cells were then collected via high‐speed centrifugation and washed twice with prechilled PBS. Subsequently, lysis buffer was added to lyse the cells on ice for 15 min, followed by sonication to fragment the chromatin to a size ranging from 200 to 600 bp. An anti‐H3K79me1 antibody (CST, Beverly, MA, USA; 12522S) or a control IgG antibody (Sigma‒Aldrich, Burlington, MA, USA; 12–370) was used to immunoprecipitate the fragmented chromatin, which was subsequently incubated overnight at 4 °C. After incubation, protein A/G magnetic beads were used to recover the antibody‐target protein‒DNA complex. To release the DNA fragments, a decrosslinking buffer containing proteinase K and NaCl was introduced. DNA purification was carried out using a Qiagen kit in accordance with the manufacturer's instructions, and the purified DNA was subsequently utilized for qPCR analysis with the primers described in Table S4 (Supporting Information).

### Animal Models and Treatment

All animal experiments in this study were conducted in strict accordance with the guidelines approved by the Experimental Animal Welfare and Ethics Committee of the Third Military Medical University (AMUWEC20211175). The MMTV‐ErbB2 transgenic mouse model used in this research was sourced from the Jackson Laboratory's colony in Sacramento, California, USA. Only female mice were used in this study, and the mice were genotyped via PCR amplification and agarose gel electrophoresis to ensure genetic uniformity with the primers described in Table S5 (Supporting Information). The heterozygous MMTV‐ErbB2 mice developed mammary tumors with an average latency period of 23 weeks, and only virgin female mice were selected for the experiments. The experimental environment simulated a natural light‒dark cycle, consisting of a 12‐h alternation of light and darkness (from 7:00 AM to 7:00 PM), and ample food and water were provided to the experimental animals. Six‐week‐old female MMTV‐ErbB2 mice were randomly assigned to four groups: a) the control group, b) the MTA treatment group, c) the Cd exposure group, and d) the combined MTA treatment and Cd exposure (MTA+Cd) group, each comprising eight mice. The mice received intraperitoneal injections of 1% DMSO, either containing MTA (96 µmol kg^−1^ body weight) or not, twice weekly. The MTA dosage has been previously shown to be effective and nontoxic in other in vivo models.^[^ ^29^, ^102^, ^103^ ^]^ In both the Cd exposure group and the MTA+Cd group, the mice were exposed to Cd via their drinking water for 23 weeks at a concentration of 3.6 mg L^−1^.^[^ ^11^, ^12^ ^]^ The Cd‐supplemented drinking water was replaced twice a week.

### Determination of Tumorigenesis

All the mice were palpated twice weekly to assess tumor growth. When the mice reached 29 weeks of age or the tumor diameter exceeded 1.5 cm, euthanasia was performed by cervical dislocation under deep anesthesia, following a previously described method.^[^ ^11^ ^]^ Under stringent aseptic protocols, the mice were subjected to anatomical dissection. The primary tumors of MMTV‐ErbB2 mice were isolated via dissection of the overlying skin, surrounding adipose tissue, and lymph nodes using sterile surgical scissors. Specifically, tumors from the fourth mammary gland (inguinal) were collected as representative samples for study.^[^ ^12^ ^]^ The tumor volume was calculated on the basis of the length and width of the tumor (V = 1/2 × length × width).^[^ ^18^ ^]^

### Hematoxylin‒Eosin and Immunofluorescence Staining

Fresh organ samples, including mammary tumor, liver, lung, and jejunum samples, were immediately collected and fixed with 4% paraformaldehyde for histopathological observation of tumor proliferation and metastasis. After fixation, the organ samples were dehydrated in ethanol and then embedded in paraffin, from which 5 µm‐thick sections were prepared for hematoxylin‐eosin and immunofluorescence staining. Comprehensive information on the antibodies used is provided in Table S2 (Supporting Information).

### Transmission Electron Microscopy

Fresh breast tumor tissues were collected and sectioned into 1 mm^3^ cubes, followed by fixation in 2.5% glutaraldehyde and subsequent resin embedding. Ultrathin sections were prepared for examination of ultrastructures with a transmission electron microscope.^[^ ^2^ ^]^

### Statistical Analysis

Data were analyzed using GraphPad Prism 9.0 and were presented as the means ± standard deviations or means ± standard errors of the means according to the experimental design. Normality was assessed via the Shapiro‒Wilk test, and homogeneity of variance was assessed via Levene's test. For data violating parametric assumptions, nonparametric tests were applied. Two‐group comparisons were performed via unpaired two‐tailed t tests (parametric) or Mann‒Whitney U tests (nonparametric). For multigroup comparisons, one‐way ANOVA followed by Tukey's HSD post hoc test or the Kruskal‒Wallis test with Dunn's correction was used, with adjusted p values reported. A log‐rank test was performed to establish differences in tumor appearance. Statistical significance was defined as two‐tailed *p* < 0.05. The experiments included ≥ 3 biological replicates.

## Conflict of Interest

The authors declare no conflict of interest.

## Author Contributions

J.L., P.D., T.F., Y.Q., and M.T. contributed equally to this work. H.P., Z.Z., Z.Y., and T.F. designed the project. J.L., P.D., Y.Q., M.T., Y.L., P.G., Y.P., M.Q., S.J., R.H., L.W., L.Z., C.C., M.H., Q.M., Y.L., L.T., J.X., M.C., M.L., and R.T. performed the experiments. J.L., P.D., and M.T. analyzed the data. J.L. and H.P. wrote the manuscript. H.P., Z.Z., and Z.Y. reviewed and edited the manuscript.

## Supporting information



Supporting Information

## Data Availability

The data supporting the findings of this study can be obtained from the corresponding author upon reasonable request.
