# Supplementary material for: 5’‐Methylthioadenosine Metabolic Reprogramming Drives H3K79 Monomethylation‐Mediated PAK2 Upregulation to Promote Cadmium‐Induced Breast Cancer Progression by Impairing Autophagic Flux
Source: Adv Sci (Weinh). 2025 Sep 8;12(44):e00941. doi: 10.1002/advs.202500941 (PMC12667465; doi:10.1002/advs.202500941)

Supporting Information

**5’-Methylthioadenosine Metabolic Reprogramming Drives H3K79 Monomethylation-Mediated PAK2 Upregulation to Promote Cadmium-Induced Breast Cancer Progression by Impairing Autophagic Flux**

*Jingdian Li, Ping Deng, Tengfei Fan^*^, Yuchen Qu, Miduo Tan, Yidan Liang, Peng Gao, Yongchun Peng, Mingke Qin, Sheng Jie, Rongrong Hao, Liting Wang, Lei Zhang, Chunhai Chen, Mindi He, Qinlong Ma, Yan Luo, Li Tian, Jia Xie, Mengyan Chen, Rui Tian, Min Li, Zhengping Yu**^*^, Zhou Zhou^*^, and Huifeng Pi^*^*

**Experimental Section**

*Gene set enrichment analysis (GSEA):* GSEA was performed to systematically identify biological pathways associated with differential protein expression profiles. GSEA software (Broad Institute, v4.0.3) was used to evaluate the enrichment of C5 category (Gene Ontology) gene sets from the Molecular Signatures Database (MSigDB)^[1]^. Differentially expressed proteins (DEPs) were ranked using the signal-to-noise ratio metric. The analysis utilized 1,000 permutations and incorporated the weighted enrichment statistic^[2]^, with gene set size filtering (15–500 genes) to improve reliability. Differences with an absolute normalized enrichment score (|NES|) >1.5 and a false discovery rate (FDR) q value <0.05 were defined as statistically significant^[3]^. All the raw proteomic data and GSEA results have been deposited in the BIG submission portal under the dataset identifier PRJCA033458.

*KEGG pathway enrichment analysis:* Pathway enrichment analysis via the Kyoto Encyclopedia of Genes and Genomes (KEGG) database (https://www.kegg.jp/) was performed for the DEPs and differentially abundant metabolites (DAMs). The species-specific protein set served as the protein background, whereas the KEGG metabolite database was used as the metabolite background. DEPs and metabolites were analyzed against their corresponding backgrounds via hypergeometric tests (see formula in Figure S14A). The Benjamini‒Hochberg procedure was applied to control the false discovery rate (FDR), with enrichment significance set at FDR < 0.05. The enrichment score was calculated using the formula in Figure S14B. The enrichment factor (m/M) was calculated as the ratio of differentially expressed molecules to all annotated molecules in a pathway. Enrichment plots and heatmaps of the DEPs were generated to visualize prioritized pathways.

*Ingenuity pathway analysis (IPA):* The Pearson correlation coefficient method was employed to assess the associations between the expression levels of DEPs and the magnitude of the effects on DAMs on the basis of their one-to-one sample correspondence pairs^[4,5]^. The Benjamini‒Hochberg procedure was applied for multiple testing correction, with a significance threshold of 0.05 for correlation significance. An association network diagram was subsequently constructed according to the correlation analysis results between the DEPs and the DAMs. Network analysis of DEPs identified via proteomics and DAMs identified via metabolomics was performed using IPA software (Qiagen, Redwood City, CA, USA), which yielded a metabolite‒protein interaction network.

*CUT&Tag:* CUT&Tag experiments were performed by Jingjie PTM Biolab Co., Ltd. In brief, 1 × 10^6^ cells were harvested and washed twice with 200 μl of CUT&Tag wash buffer (20 mM HEPES pH 7.5, 150 mM NaCl, 0.5 mM spermidine, and 1× protease inhibitor cocktail (Roche)). The cells were bound to concanavalin A-conjugated magnetic beads (ConA beads) for 10 min at room temperature, followed by permeabilization with 0.01% digitonin in wash buffer. The cells were incubated overnight at 4°C in a thermomixer (BIONOON-100HH) with either an anti-H3K79me1 antibody (Active Motif, Carlsbad, CA, USA; 39921) or control IgG (Sigma‒Aldrich, Burlington, MA, USA; 12–370) diluted in antibody buffer. Unbound primary antibody was removed using a magnetic stand (Vazyme CM101), and then secondary antibody (1:100 in 100 μL of Dig-Wash Buffer) was added for 60 min at room temperature. The pA-Tn5 transposase complexes (1:100 in 100 μL of dig-med buffer containing 0.05% digitonin, 20 mM HEPES pH 7.5, 300 mM NaCl, 0.5 mM spermidine, and 1× protease inhibitor) were subsequently incubated for 1 h at room temperature. Libraries were amplified via the TruePrep Index Kit V2 (Illumina) under the following cycling conditions: 72 °C for 5 min; 98 °C for 30 s; 12 cycles of 98 °C for 10 s and 63 °C for 30 s; and a final extension at 72 °C for 1 min and a hold at 4 °C^[6]^. Amplified DNA was purified with VAHTS DNA Clean Beads (Vazyme), quantified on an Agilent 2100 Bioanalyzer, and sequenced in paired-end mode (2×150 bp) on an Illumina NovaSeq 6000.

*Clinical samples**:* BC tissue samples were collected from the Central Hospital of Zhuzhou City. Samples were taken from 80 patients with luminal-type BC who had not undergone neoadjuvant therapy prior to the surgical procedure from January 2018 to December 2019, which was followed by a histological examination to diagnose BC. All patients involved in the study provided written informed consent beforehand. The ethics committee of the Central Hospital of Zhuzhou City approved this research (NO. YILUN20170988). MTA levels in fresh BC tissue samples were analyzed via liquid chromatography–mass spectrometry (LC–MS), which was performed by Servicebio Technology. To categorize patients into high or low MTA groups, the median MTA level (rounded) served as the cutoff value^[5]^. Tumors with MTA levels exceeding this threshold were classified as having high MTA levels, whereas those below this threshold were classified as having low MTA levels. Statistical analyses employed chi-square or adjusted chi-square tests to assess associations between MTA levels and clinicopathological parameters. For all tests, the type I error rate was set at 0.05, and p values < 0.05 were considered to indicate statistical significance.

**References**

[1] H. A. Carey, B. E. Hildreth, J. A. Geisler, M. C. Nickel, J. Cabrera, S. Ghosh, Y. Jiang, J. Yan, J. Lee, S. Makam, N. A. Young, G. R. Valiente, W. N. Jarjour, K. Huang, T. J. Rosol, R. E. Toribio, J. F. Charles, M. C. Ostrowski, S. M. Sharma, *Bone Res.* **2018**, *6*, 8.

[2] H.-H. Lu, S.-Y. Lin, R. R. Weng, Y.-H. Juan, Y.-W. Chen, H.-H. Hou, Z.-C. Hung, G. A. Oswita, Y.-J. Huang, S.-Y. Guu, K.-H. Khoo, J.-Y. Shih, C.-J. Yu, H.-C. Tsai, *EBioMedicine* **2020**, *57*, 102846.

[3] N. Giménez, R. Schulz, M. Higashi, M. Aymerich, N. Villamor, J. Delgado, M. Juan, M. López-Guerra, E. Campo, L. Rosich, M. Seiffert, D. Colomer, *Leukemia* **2020**, *34*, 100.

[4] L.-C. Kong, J. Tap, J. Aron-Wisnewsky, V. Pelloux, A. Basdevant, J.-L. Bouillot, J.-D. Zucker, J. Doré, K. Clément, *Am. J. Clin. Nutr.* **2013**, *98*, 16.

[5] J. Declercq, K. F. A. Van Damme, E. De Leeuw, B. Maes, C. Bosteels, S. J. Tavernier, S. De Buyser, R. Colman, M. Hites, G. Verschelden, T. Fivez, F. Moerman, I. K. Demedts, N. Dauby, N. De Schryver, E. Govaerts, S. J. Vandecasteele, J. Van Laethem, S. Anguille, J. Van Der Hilst, B. Misset, H. Slabbynck, X. Wittebole, F. Liénart, C. Legrand, M. Buyse, D. Stevens, F. Bauters, L. J. M. Seys, H. Aegerter, U. Smole, V. Bosteels, L. Hoste, L. Naesens, F. Haerynck, L. Vandekerckhove, P. Depuydt, E. Van Braeckel, S. Rottey, I. Peene, C. Van Der Straeten, F. Hulstaert, B. N. Lambrecht, *Lancet Respir. Med.* **2021**, *9*, 1427.

[6] R. S. Ziffra, C. N. Kim, J. M. Ross, A. Wilfert, T. N. Turner, M. Haeussler, A. M. Casella, P. F. Przytycki, K. C. Keough, D. Shin, D. Bogdanoff, A. Kreimer, K. S. Pollard, S. A. Ament, E. E. Eichler, N. Ahituv, T. J. Nowakowski, *Nature* **2021**, *598*, 205.

**Figures and Legends**


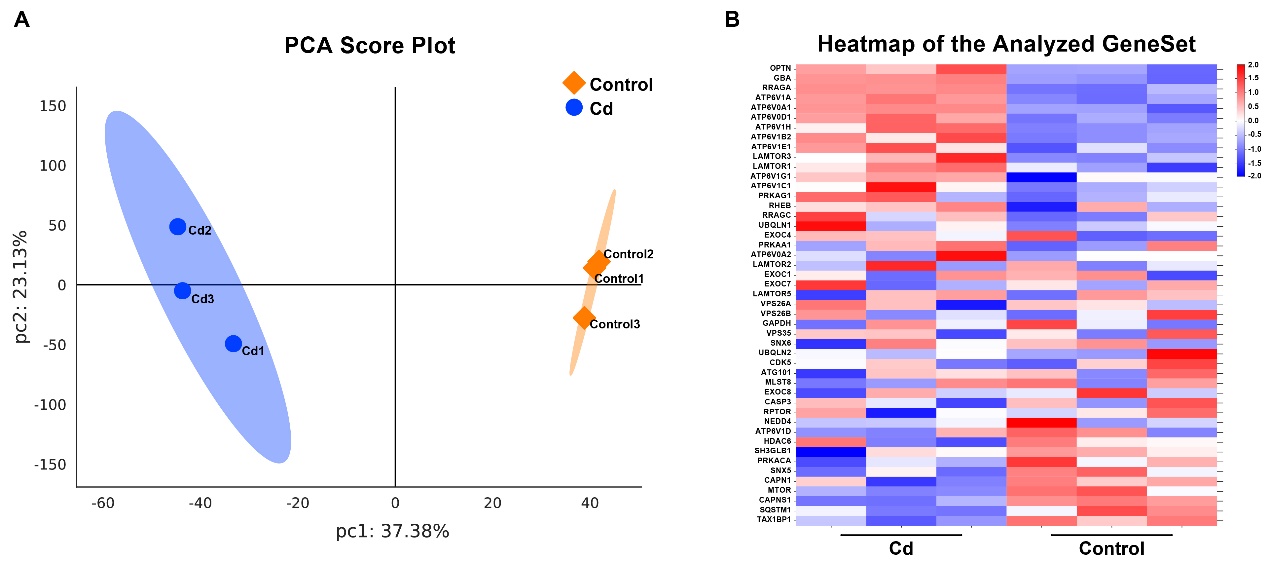


Figure S1. Proteomics principal components analysis (PCA) and heatmap analysis. (A) PCA plot of proteomics data. (B) Heatmap displaying relative expression levels of autophagy-related proteins identified through GSEA and KEGG pathway enrichment (|Fold Change| >1.5 and FDR <0.05).


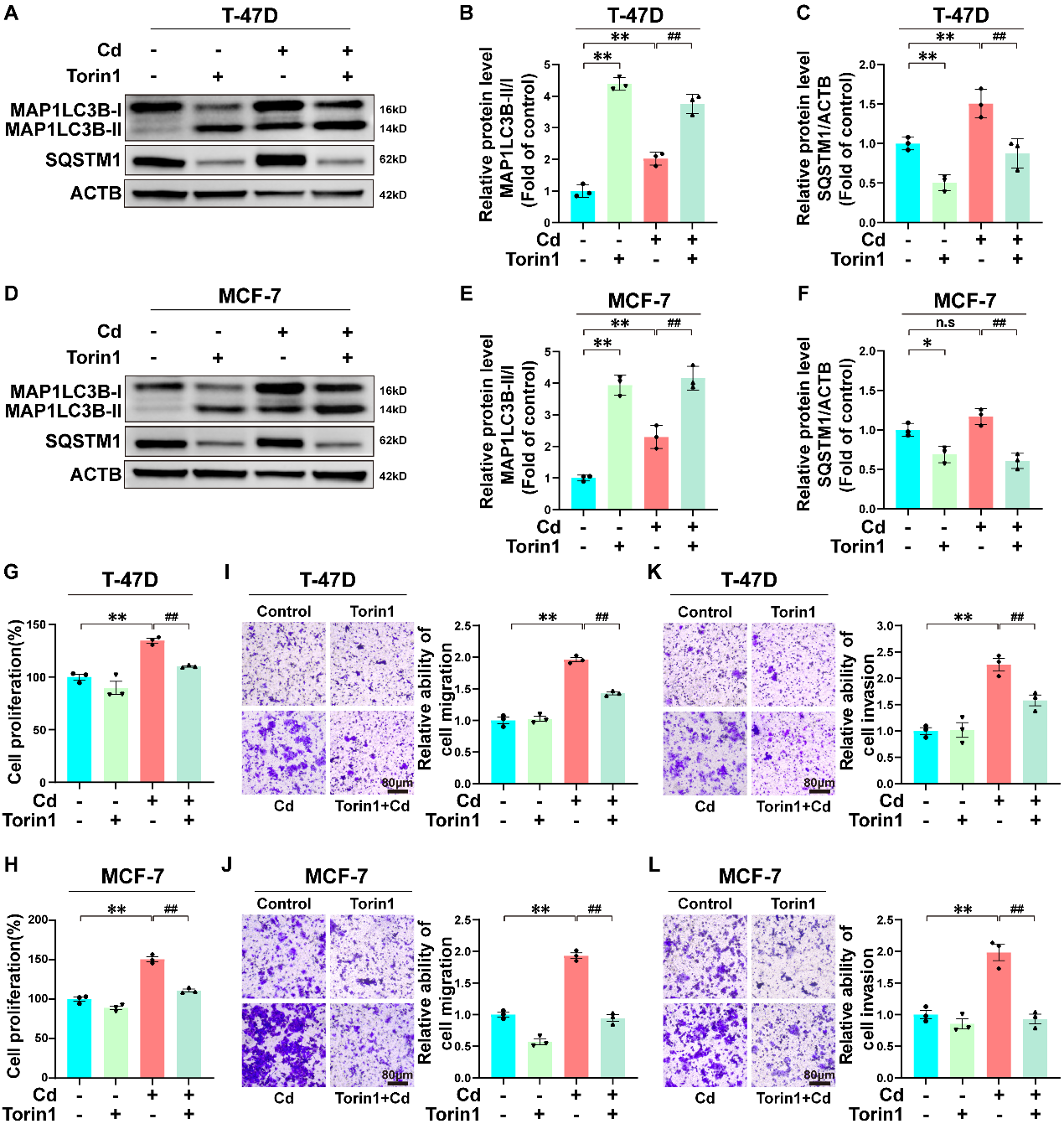


Figure S2. Torin1 antagonized the Cd-induced proliferation, migration, and invasion of BC cells by increasing autophagic flux. In A-L, all T-47D and MCF-7 cells were treated with or without 100 nM Torin 1 in the absence or presence of 6 μM Cd for 72 h. Immunoblots (A) and quantification (B, C) of MAP1LC3B and SQSTM1 levels in T-47D cells. Immunoblots (D) and quantification (E, F) of MAP1LC3B and SQSTM1 levels in MCF-7 cells. (G, H) Proliferative activity of T-47D and MCF-7 cells. (I, J) Migratory capabilities of T47D and MCF7 cells. Scale bar: 80 μm. (K, L) The invasive capabilities of T47D and MCF7 cells. Scale bar: 80 μm. **p* <0.05 and ***p* <0.01 compared with the control group. ^##^*p* <0.01 compared with the Cd group. ns: not significant (*Tukey’s HSD*).


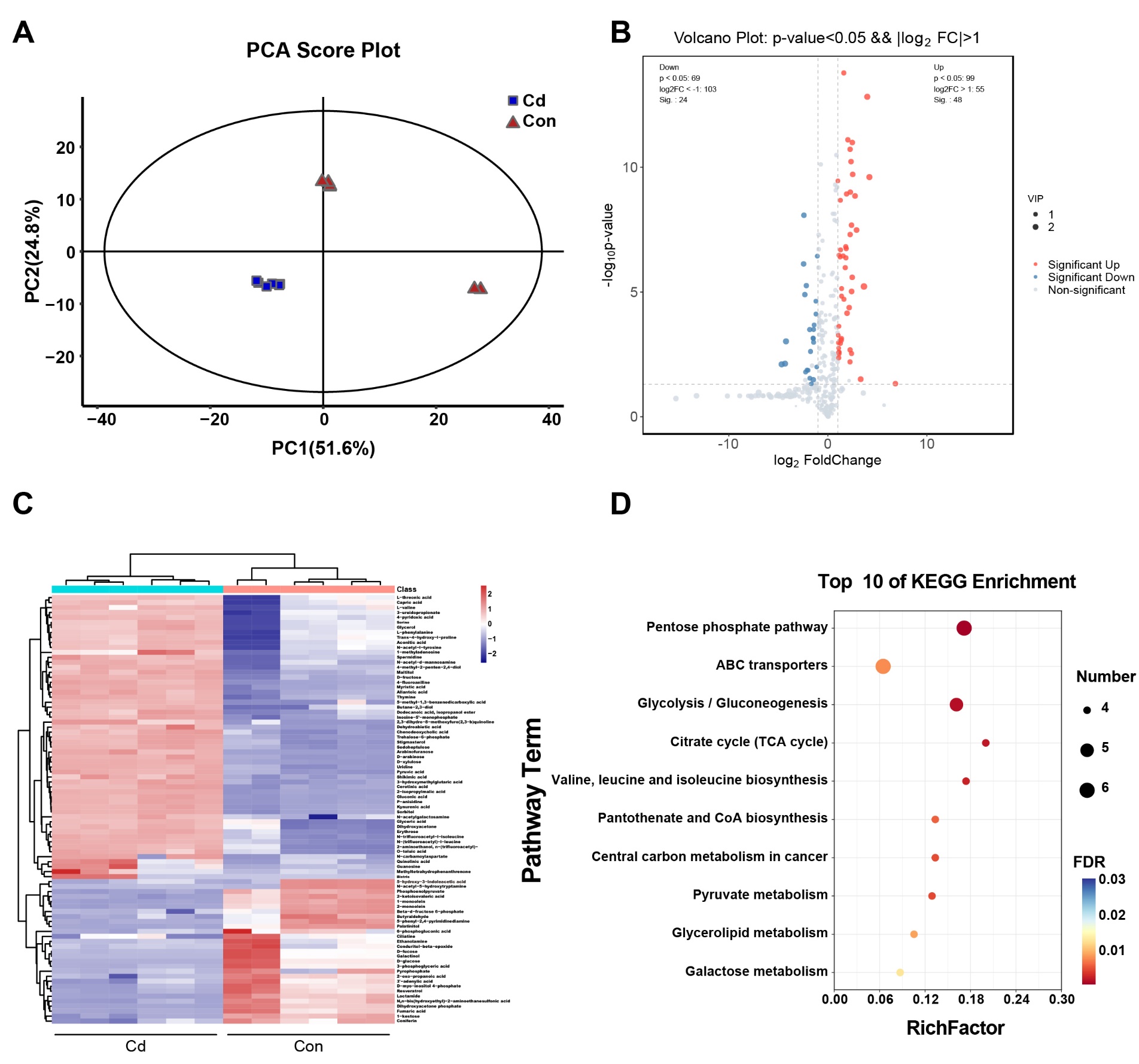


Figure S3. Cd exposure alters the metabolic profile of T-47D cells (GC-MS). (A) Principal components analysis plot of the metabolomics analysis. (B) Volcano plot of the metabolomics. (C) Clustering heatmap of the DAMs (|Fold Change| >2, FDR <0.05 and variable important in projection (VIP) >1). (D) Top 10 KEGG pathways associated with the DAMs.


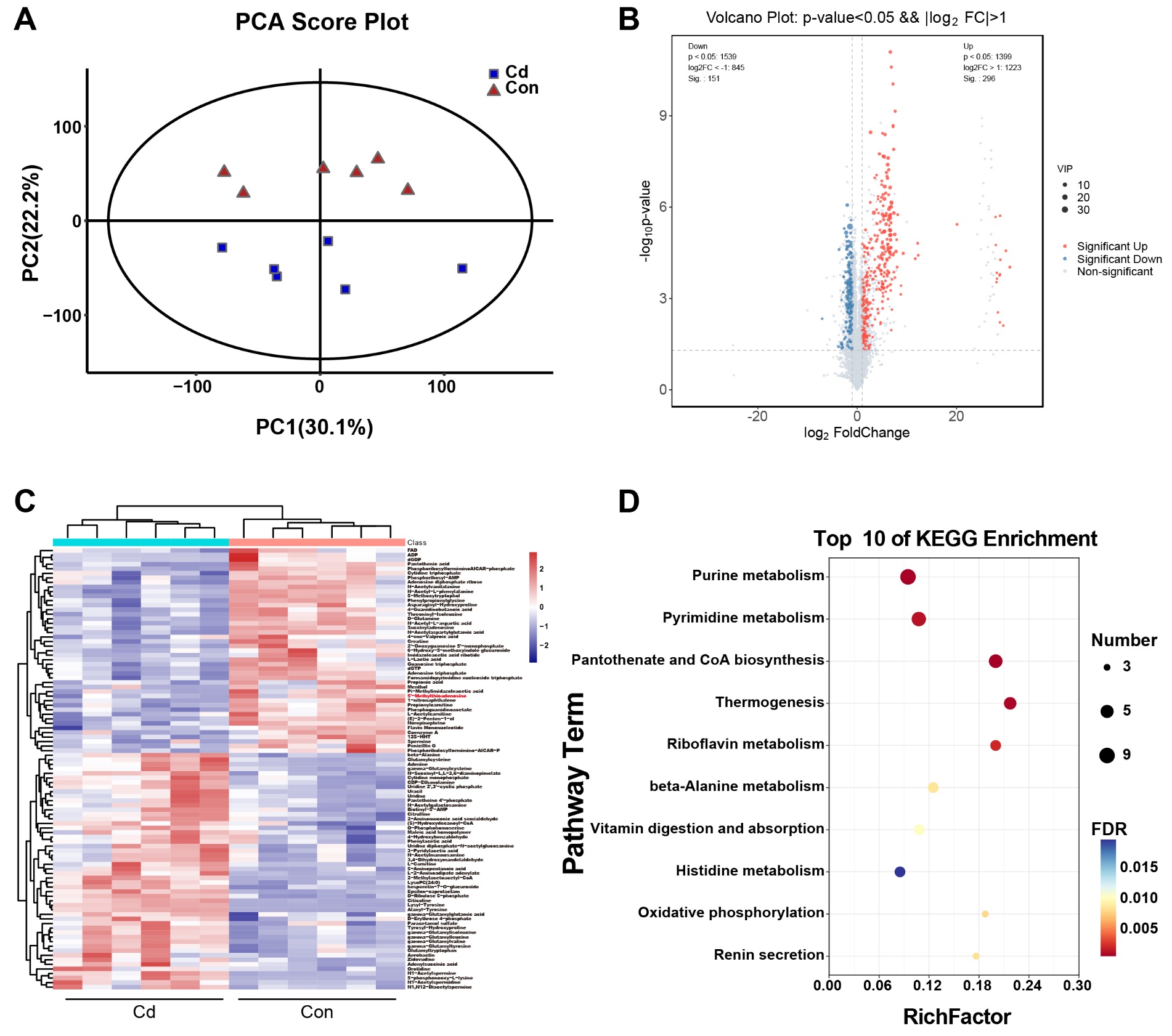


Figure S4. Cd exposure alters the metabolic profile of T-47D cells (LC -MS). (A) Principal components analysis plot of the metabolomics analysis. (B) Volcano plot of the metabolomics. (C) Clustering heatmap of the DAMs (|Fold Change| >2, FDR <0.05 and VIP >1). (D) Top 10 KEGG pathways associated with the DAMs.


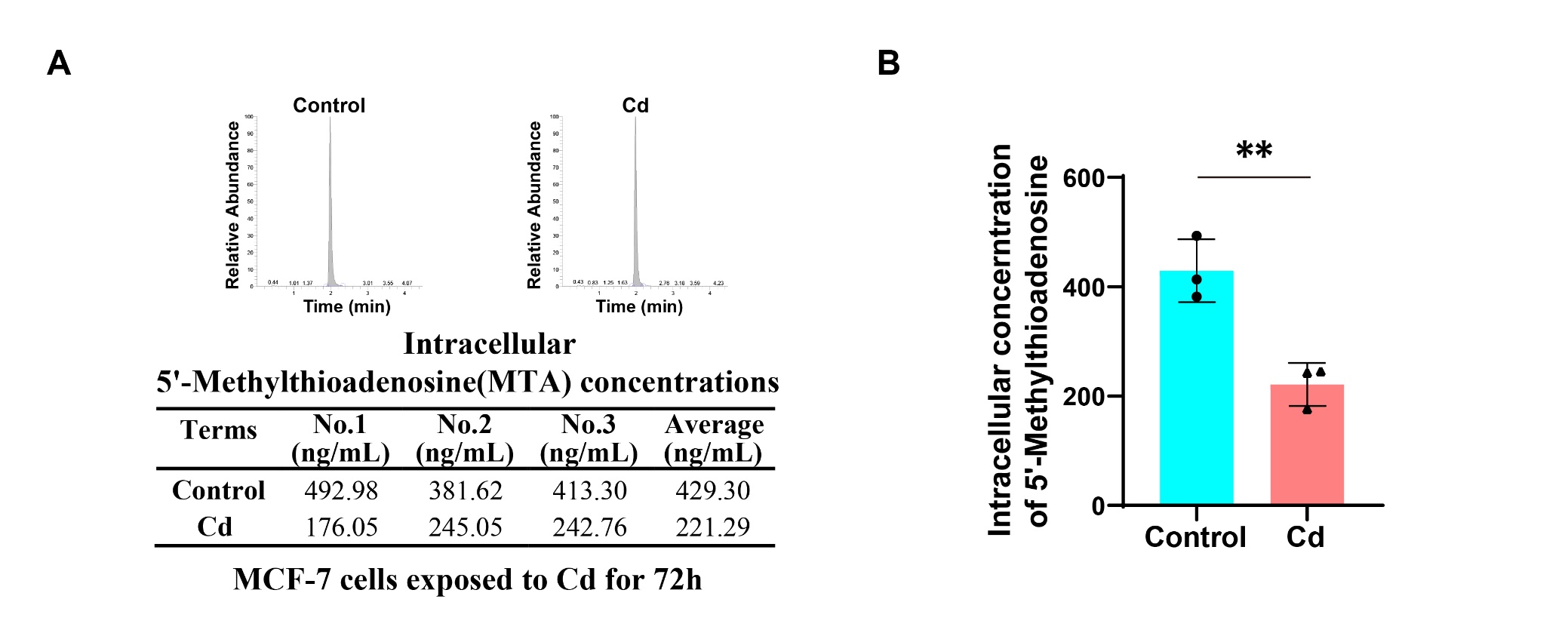


Figure S5. LC/MS chromatogram (A) and quantification analysis (B) of intracellular MTA concentrations in MCF-7 cells. ***p* <0.01 vs. the control group.


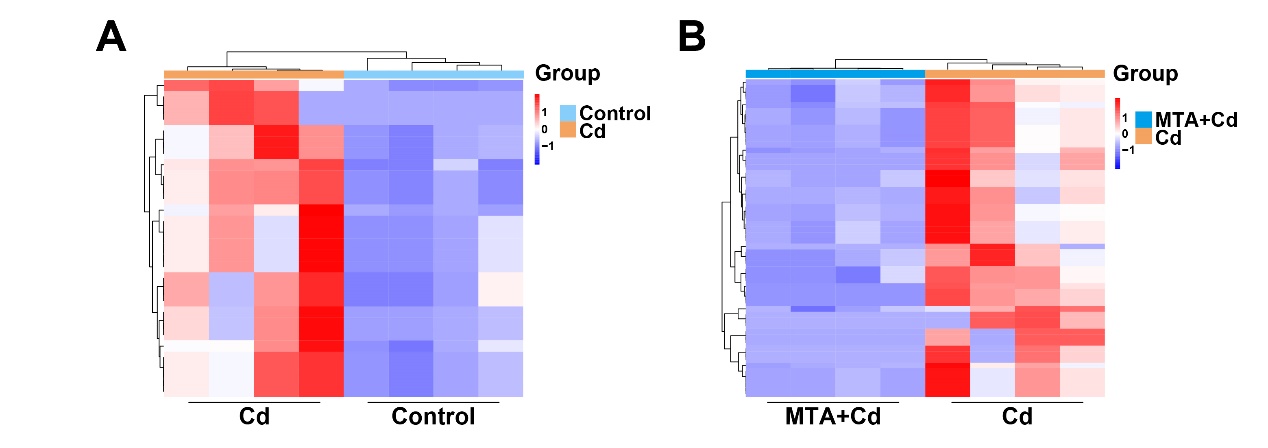


Figure S6. Histone peptides with differential methylation modifications, as identified by LC-MS/MS (|Fold Change| >2, FDR <0.05). (A) Clustering heatmap of the differential methylation modifications (Cd vs. Control). (B) Clustering heatmap of the differential methylation modifications (MTA + Cd vs. Cd).


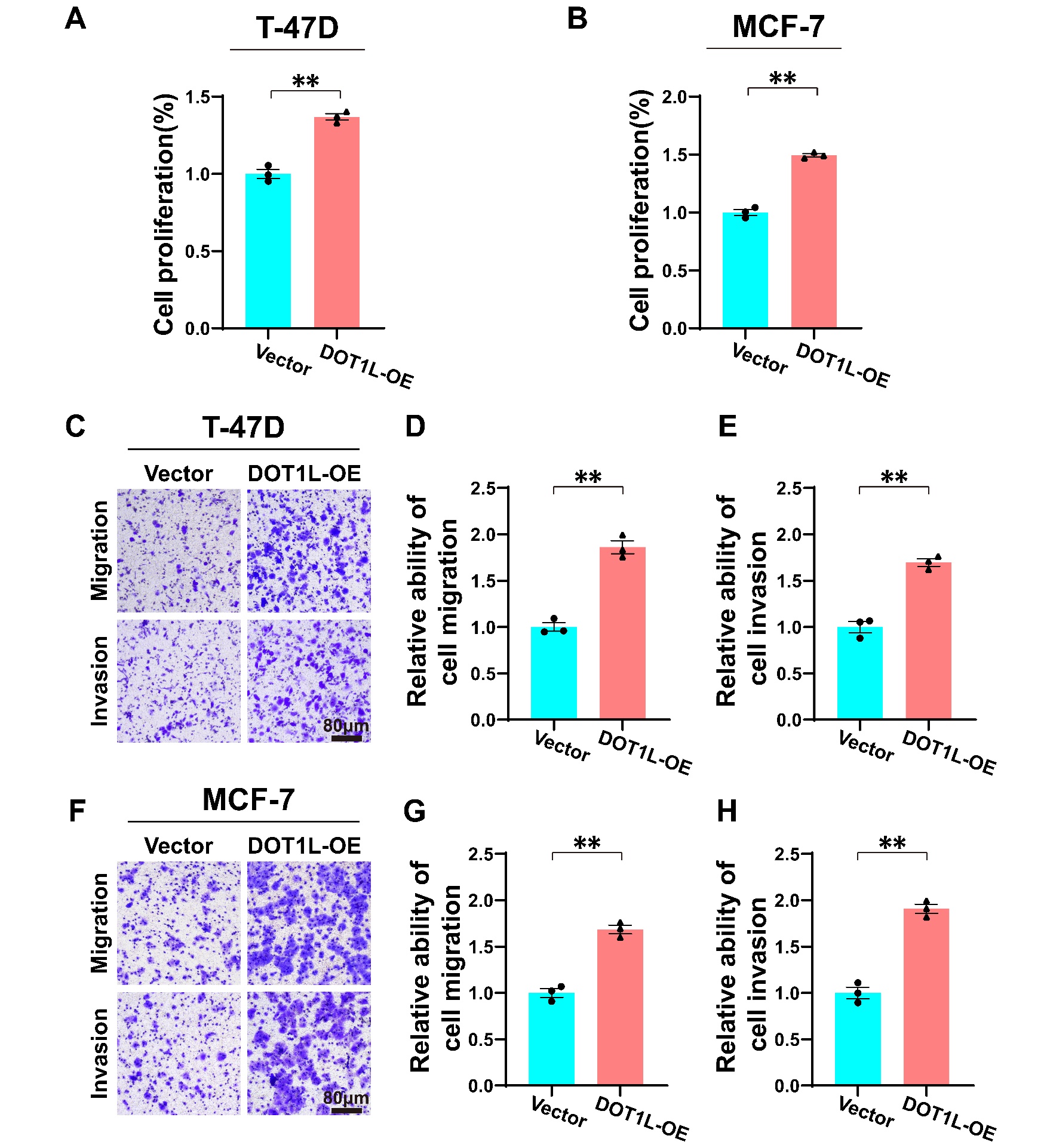


Figure S7. Overexpression of DOT1L promotes proliferation, migration, and invasion in T-47D and MCF-7 cells. (A, B) The proliferative activity of T-47D and MCF-7 cells. (C, D and E) The migratory capabilities and invasive ability of T-47D cells. Scale bar: 80 μm. (F, G and H) The migratory capabilities and invasive ability of MCF-7 cells. Scale bar: 80 μm.


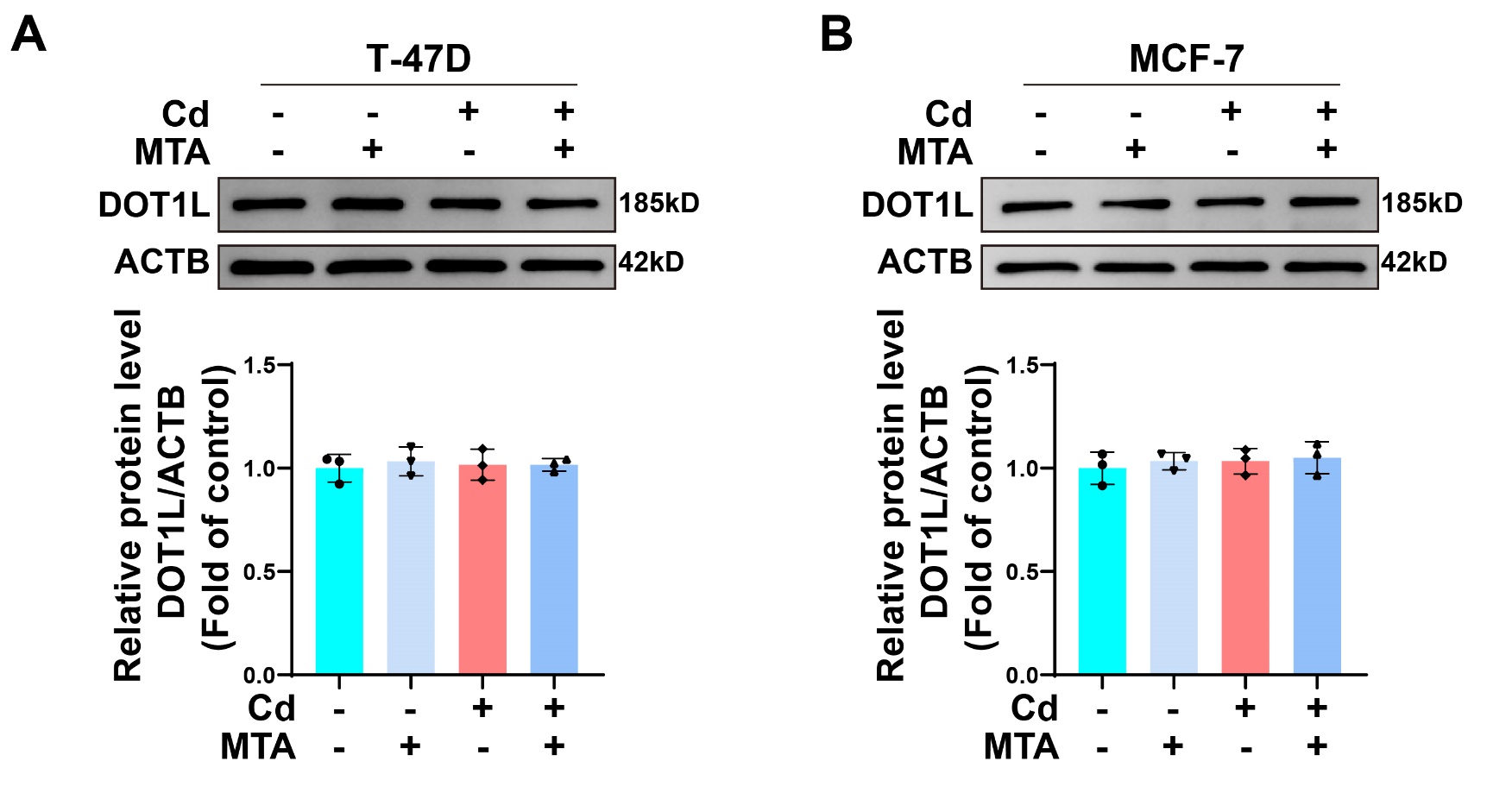


Figure S8. Cd or/and MTA treatments show no significant effect on DOT1L expression levels in T-47D (A) and MCF-7 (B) cells.


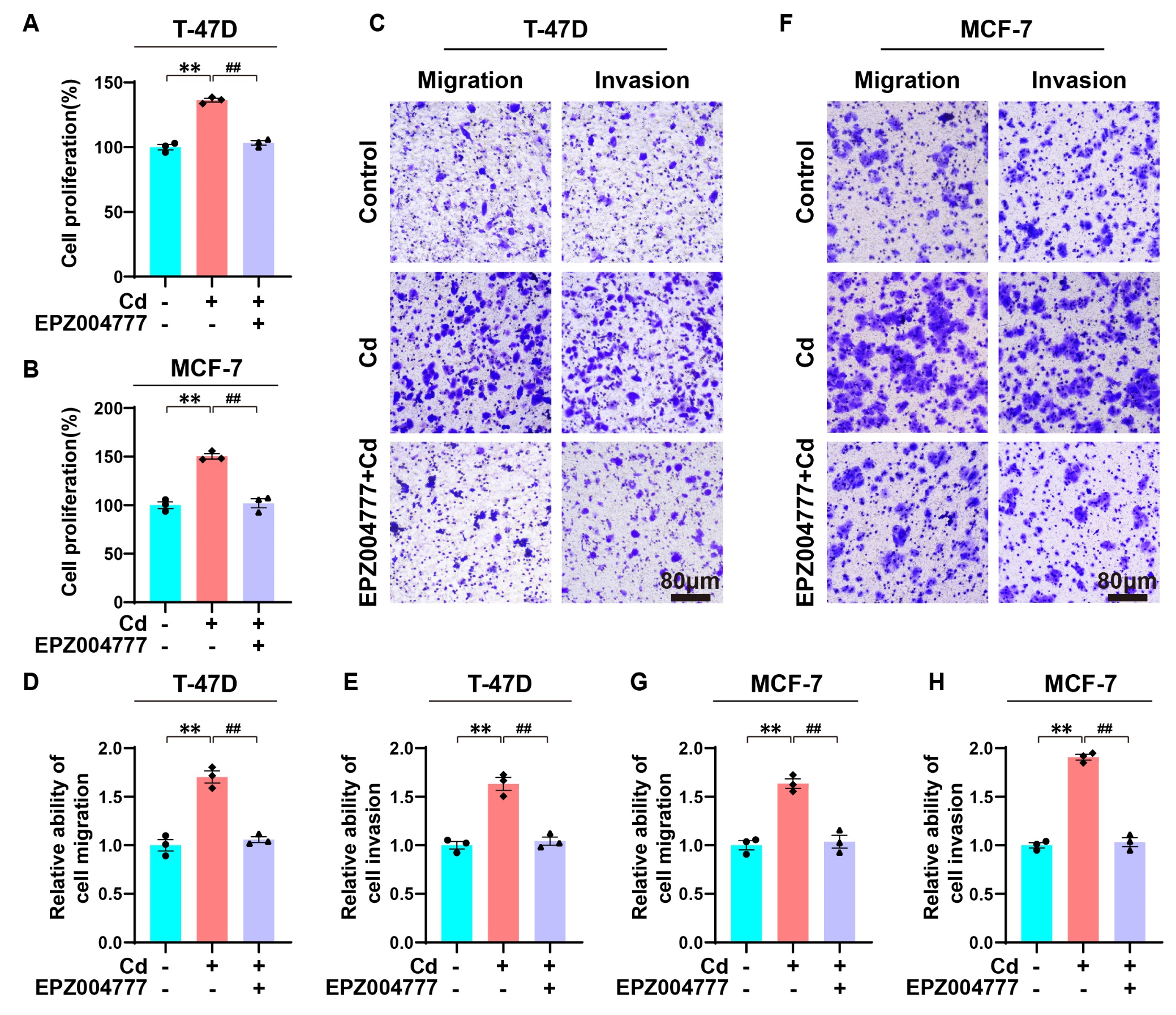


Figure S9. EPZ004777 hydrochloride antagonized the Cd-induced proliferation, migration, and invasion of BC cells. In A-H, all T-47D and MCF-7 cells were treated with or without 50 μM EPZ004777 hydrochloride in the absence or presence of 6 μM Cd for 72 h. (A, B) Proliferative activity of T-47D and MCF-7 cells. (C-E) The migratory capabilities and invasive ability of T47D cells. Scale bar: 80 μm. (F-H) The migratory capabilities and invasive ability of MCF7 cells. Scale bar: 80 μm. **p* <0.05 and ***p* <0.01 compared with the control group. ^##^*p* <0.01 compared with the Cd group. ns: not significant (*Tukey’s HSD*).


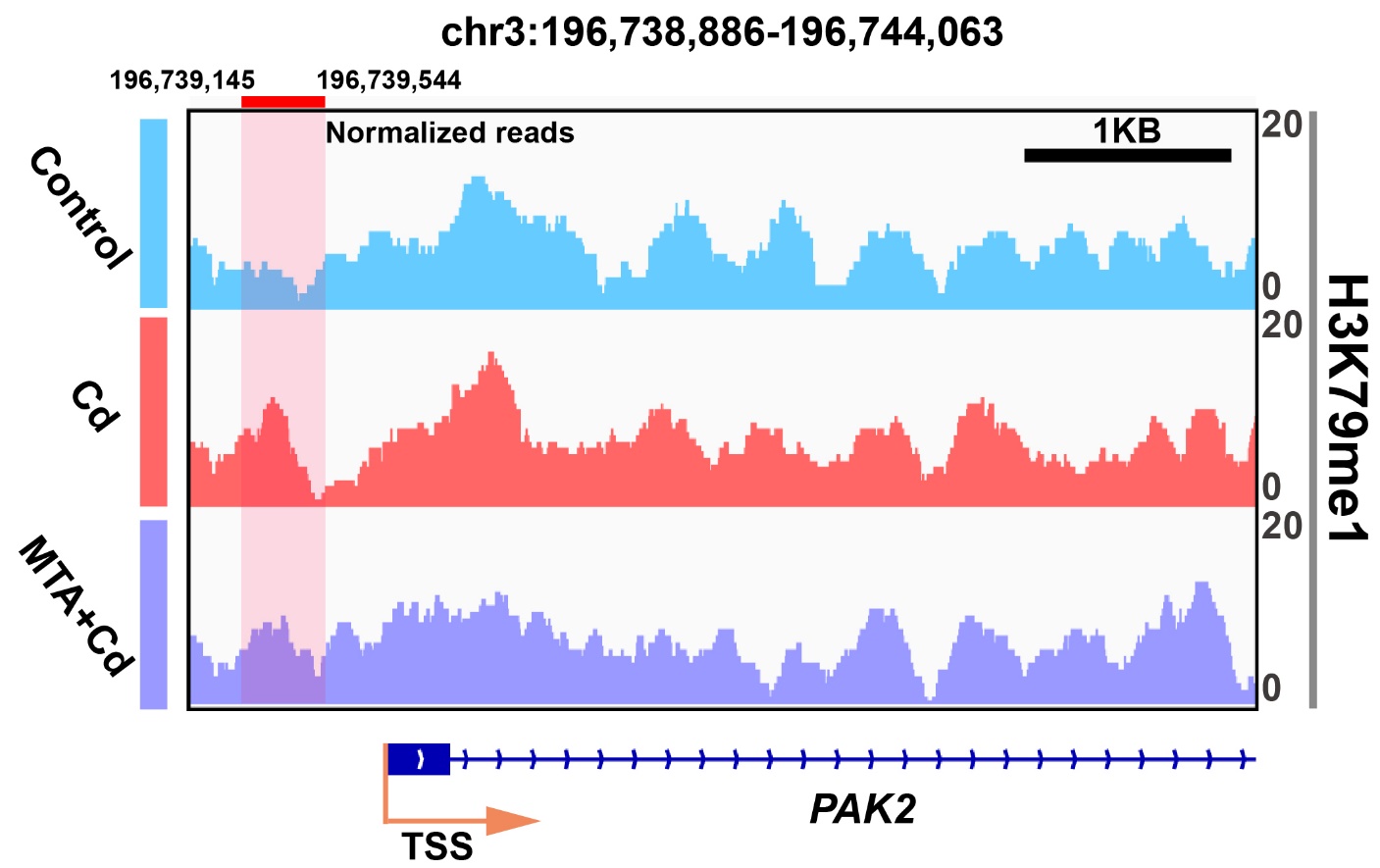


Figure S10. Integrative Genomics Viewer tracks of H3K79me1 peaks at the PAK2 promoter region.


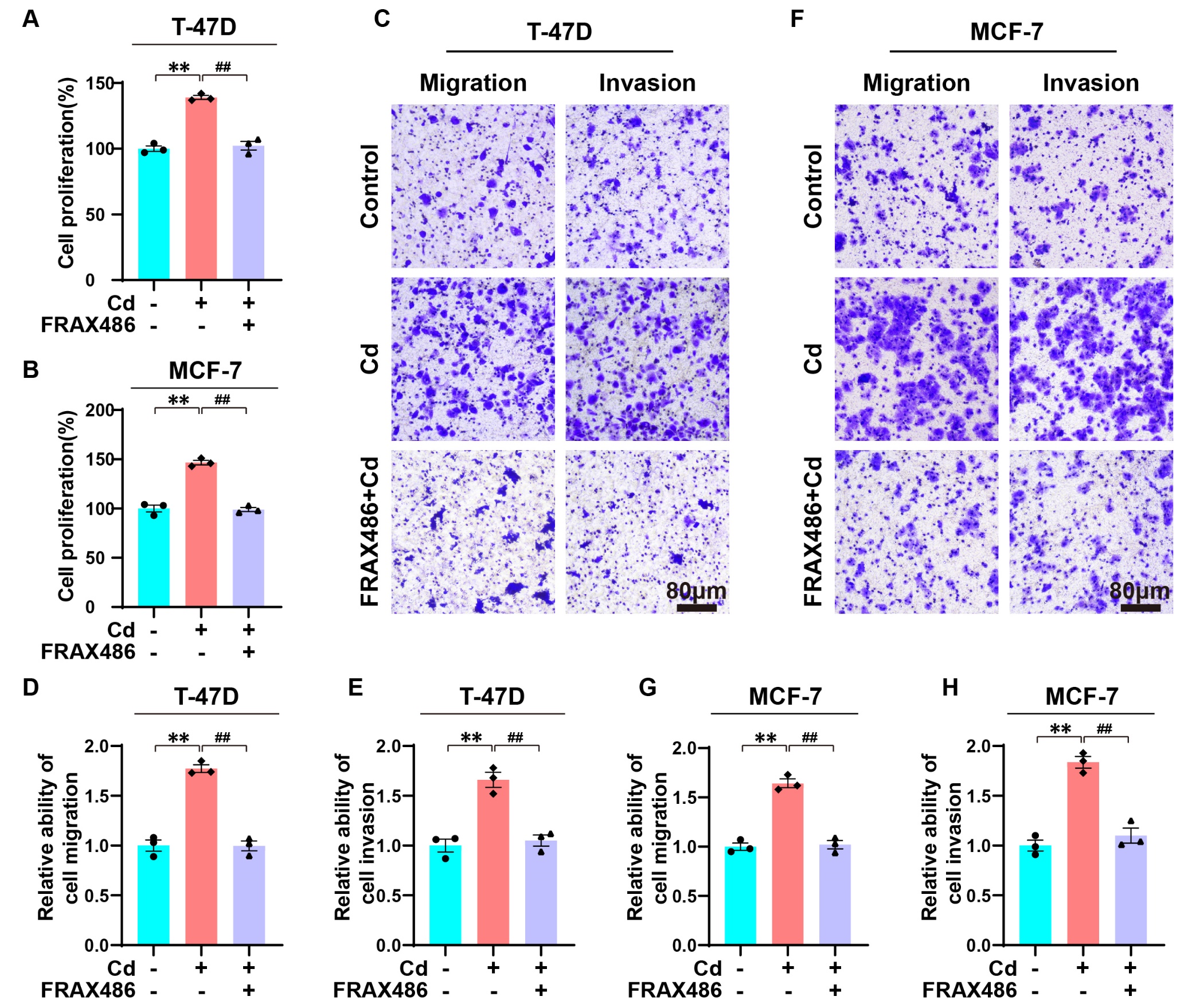


Figure S11. FRAX486 antagonized the Cd-induced proliferation, migration, and invasion of BC cells. In A-F, all T-47D and MCF-7 cells were treated with or without 2 μM FRAX486 in the absence or presence of 6 μM Cd for 72 h. (A, B) Proliferative activity of T-47D and MCF-7 cells. (C-E) The migratory capabilities and invasive ability of T47D cells. Scale bar: 80 μm. (F-H) The migratory capabilities and invasive ability of MCF7 cells. Scale bar: 80 μm. **p* <0.05 and ***p* <0.01 compared with the control group. ^##^*p* <0.01 compared with the Cd group. ns: not significant (*Tukey’s HSD*).


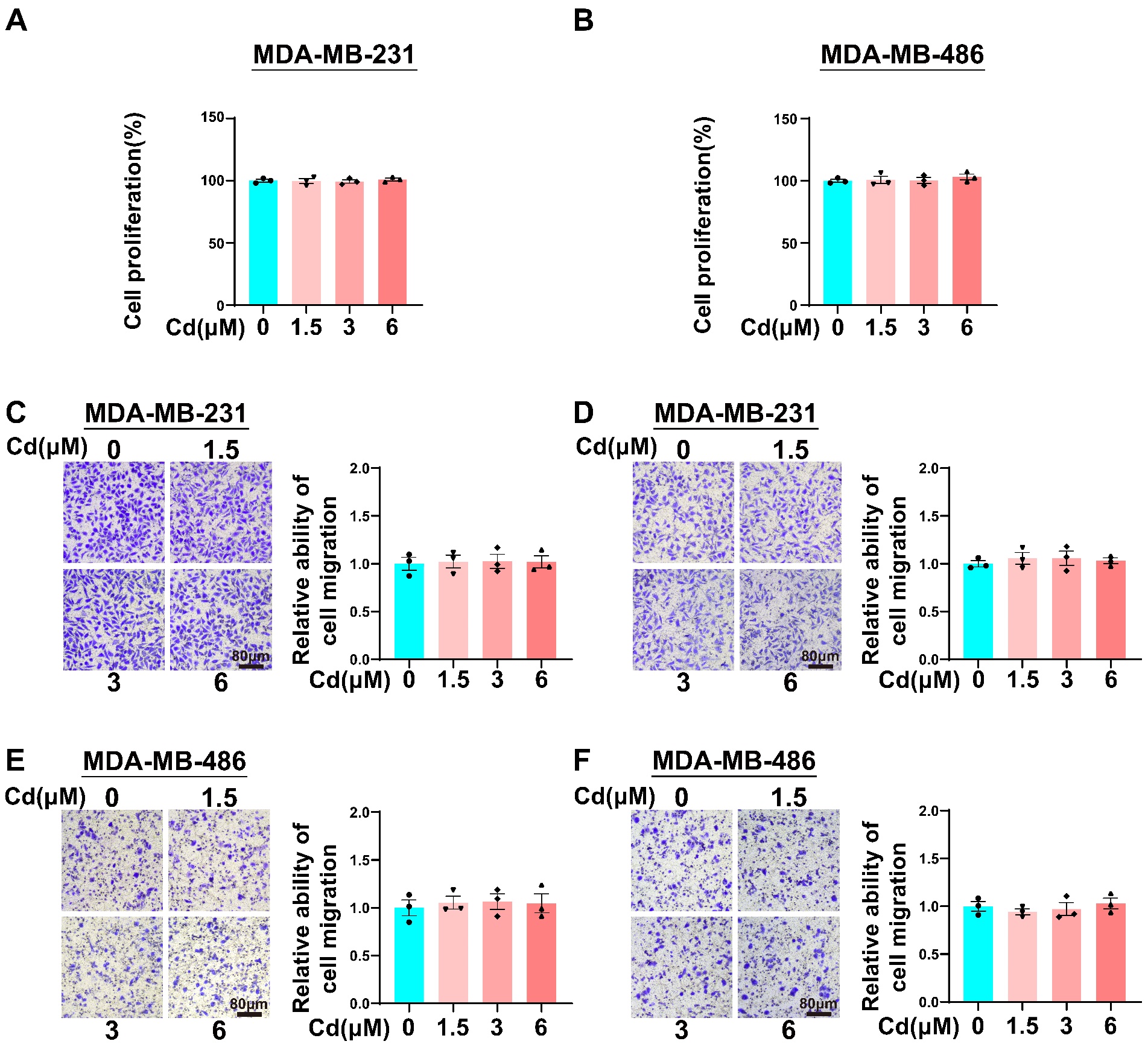


Figure S12. Cd exposure induces no significant alterations in proliferation, migration, or invasion capacities of MDA-MB-231 and MDA-MB-468 cells. (A, B) Proliferative activity of MDA-MB-231 and MDA-MB-468. (C, D) The migratory capabilities and invasive ability of MDA-MB-231 cells. Scale bar: 80 μm. (E, F) The migratory capabilities and invasive ability of MDA-MB-468 cells. Scale bar: 80 μm.


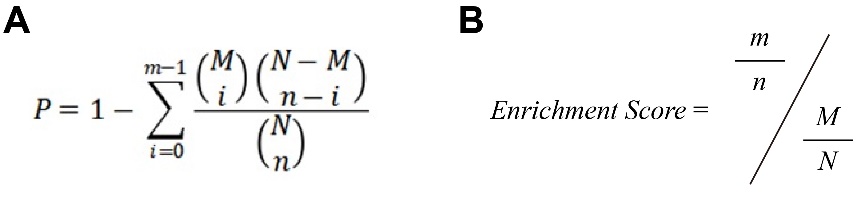


Figure S13. Formulas used in KEGG enrichment analysis. (A) Calculation of P-value using the hypergeometric distribution. (B) Computation of the enrichment score. *N* represents the total number of species-specific proteins with KEGG annotations, *n* denotes the number of DEPs /DAMs bearing annotations, *M* indicates the protein count annotated to a specific pathway, and *m* corresponds to the DEPs /DAMs mapped to that pathway.

**Table S1 The association between MTA levels and clinical**

**characteristics of breast cancer patients**

|  | **MTA level** | | ***P*** |
| --- | --- | --- | --- |
|  | **High** | **Low** |  |
| **Age** |  |  | 0.812 |
| ≥45 | 28 | 26 |  |
| ＜45 | 12 | 14 |  |
| **Histology grade** |  |  | 0.402 |
| Well-moderately (SBR I-II) | 34 | 30 |  |
| Poorly-signet (SBR III) | 6 | 10 |  |
| **Tumor size** |  |  | 0.310 |
| ≤2cm | 13 | 8 |  |
| ＞2cm | 27 | 32 |  |
| **Lymph node metastasis** |  |  | **0.013** |
| Negative | 26 | 14 |  |
| Positive | 14 | 26 |  |
| **TNM Stage** |  |  | **0.015** |
| I/II | 36 | 26 |  |
| III/IV | 4 | 14 |  |

**Table S2. Summary of the shared differentially methylated peptides identified for Cd vs Control (upregulated) and MTA+Cd vs Cd (downregulated)**

| **Peptide Sequence** | **m/z** | **-10lgP** | **Detected PTMs** |
| --- | --- | --- | --- |
| R.K(+28.03)SAPATGGVK(+112.05)K(+14.02)PHR.Y | 529.98 | 45.81 | H3K27me2;H3K37me1 |
| R.K(+14.02)SAPATGGVKK(+112.05)PHR.Y | 520.64 | 45.27 | H3K27me1 |
| R.K(+28.03)SAPSTGGVK(+112.05)K(+14.02)PHR.Y | 401.74 | 43.23 | H3K27me2;H3K37me1 |
| R.K(+28.03)SAPATGGVK(+14.02)K(+112.05)PHR.Y | 397.74 | 43.16 | H3K27me2;H3K36me1 |
| R.K(+28.03)SAPSTGGVK(+112.05)KPHR.Y | 398.23 | 41.03 | H3K27me2 |
| K.SAPATGGVK(+112.05)K(+14.02)PHR.Y | 716.41 | 39.38 | H3K37me1 |
| R.ISGLIYEETR(+14.02).G | 597.82 | 35.19 | H4R55me1 |
| R.EIAQDFK(+14.02).T | 432.73 | 32.50 | H3K79me1 |
| K.GTGASGSFK(+112.05)LNK(+28.03)K(+28.03)AASGEAKPK.A | 768.09 | 30.88 | H1K108me2;H1K109me2 |
| K.GTGASGSFK(+112.05)LNK(+28.03)K(+28.03)AASGEAKPK.A | 768.09 | 30.88 | H1K111me2;H1K112me2 |
| K.GTGASGSFK(+112.05)LNK(+28.03)K(+28.03)AASGEAKPK.V | 768.09 | 30.88 | H1K108me2;H1K109me2 |
| MPEPAK(+28.03)SAPAPK(+112.05)KGSK.K | 882.50 | 24.56 | H2BK5me2 |
| K.AAK(+28.03)PK(+112.05)AAK(+112.05)PKAAK.A | 511.32 | 22.40 | H1K206me2 |

**Table S3. Antibodies used in this study**

| Antigen | Catalogue number | Supplier | Dilution ratio | Application |
| --- | --- | --- | --- | --- |
| MAP1LC3B  SQSTM1  TAX1BP1 | L7543  Ab56416 | SIGMA  Abcam | 1：1000 | Western blot |
|  |  |  | 1：200 | Immunohistochemistry |
|  |  |  | 1：1000 | Western blot |
|  |  |  | 1：200 | Immunohistochemistry |
|  | 702840 | Invitrogen | 1：200 | Western blot |
|  |  |  | 1：100 | Immunohistochemistry |
| Mono-Methyl-H3K79 | 12522S | CST | 1：1000  1:50 | Western blot  ChIP |
| Mono-Methyl-H3K27 | 84932S | CST | 1：1000 | Western blot |
| Di-Methyl-H3K27  Mono-Methyl-H3K36 | 9728T | CST | 1：1000 | Western blot |
|  | 14111T | CST | 1：1000 | Western blot |
| Mono-Methyl-H3K37 | Ab272160 | Abcam | 1：1000 | Western blot |
| Histone H3 | Ab10799 | Abcam | 1：1000 | Western blot |
| Histone H3K79me1 antibody (pAb) | 39921 | Active Motif | 1：100 | CUT&Tag |
| Normal Rabbit IgG | 12-370 | Sigma-Aldrich | 1：100  1：100 | CUT&Tag  ChIP |
| DOT1L | Ab239358 | Abcam | 1：3000 | Western blot |
| PAK2  ACTB | A4553  A1978 | Abclonal  SIGMA | 1：1000 | Western blot |
|  |  |  | 1：5000 | Western blot |
| GAPDH | Ab8245 | Abcam | 1：2000 | Western blot |
| Goat Anti-Mouse IgG （HRP） | A0216 | Beyotime | 1：1000 | Western blot |
| Goat Anti-Rabbit IgG （HRP） | A0208 | Beyotime | 1：1000 | Western blot |
| IRDye 800CW Donkey Anti-Mouse | 926-32212 | LI-COR | 1：5000 | Western blot |
| IRDye 680RD Donkey anti-Rabbit | 926-68073 | LI-COR | 1：5000 | Western blot |

**Table S4. Primers used in this study**

| **Target gene** | **Primer** | **Nucleotide sequence（5'-3'）** | **Supplier** | **Applications** |
| --- | --- | --- | --- | --- |
| *DOT1L(Homo)* | F | CTGACCTACAACGACCTGATTCA | Beijing Tsingke Biotech Co., Ltd. | RT-qPCR |
|  | R | CTGCTTCTCCTTCAACAGCTTCT |  |  |
| *PLA2G4B(Homo)* | F | GCTCTTTGGCTCCGAGTTCTTTA | Beijing Tsingke Biotech Co., Ltd. | RT-qPCR |
|  | R | GCTGTTGAGGGTGGTTCTTCTAT |  |  |
| *PAK2(Homo)* | F | CCATGTTGGCTTTGATGCTGTTA | Beijing Tsingke Biotech Co., Ltd. | RT-qPCR |
|  | R | CTTCACTGTGTTGGAGTCGTAGA |  |  |
| *Pak2(Mus)* | F | GGGTCCCTCACTGATGTTGTAAC | Beijing Tsingke Biotech Co., Ltd. | RT-qPCR |
|  | R | CACAGAAGCCGAAGTCAGTAAGT |  |  |
| *ACTB(Homo)* | F | CCTTCCTGGGCATGGAGTC | Beijing Tsingke Biotech Co., Ltd. | RT-qPCR |
|  | R | TGATCTTCATTGTGCTGGGTG |  |  |
| *Actb(Mus)* | F | ACTGTCGAGTCGCGTCC | Beijing Tsingke Biotech Co., Ltd. | RT-qPCR |
|  | R | CTGACCCATTCCCACCATCA |  |  |
| *PAK2(Homo)* | F | TCTCGAACTCCCGACCTCA | Accurate Biotechnology Co., Ltd. | ChIP-qPCR |
|  | R | TGGACTAGACACTGTTAAGCATCA |  |  |

**Table S5. The detailed information of mouse genotype identification**

| Primer type | Sequence | Band Size |
| --- | --- | --- |
| Positive Control Forward  Positive Control Reverse | AGT GGC CTC TTC CAGAAA TG | WT: 0 bp  Targeted: 521 bp |
|  | TGC GAC TGT GTC TGATTT CC |  |
| Transgene Forward  Transgene Reverse | GAG CCT TCG GCACTG TCT AC | WT: 0 bp  Targeted: 202 bp |
|  | AGC TGCACT GTG GAT GTC AG |  |

**All full western blot data in the manuscript**

Fig.1J and Fig.1L


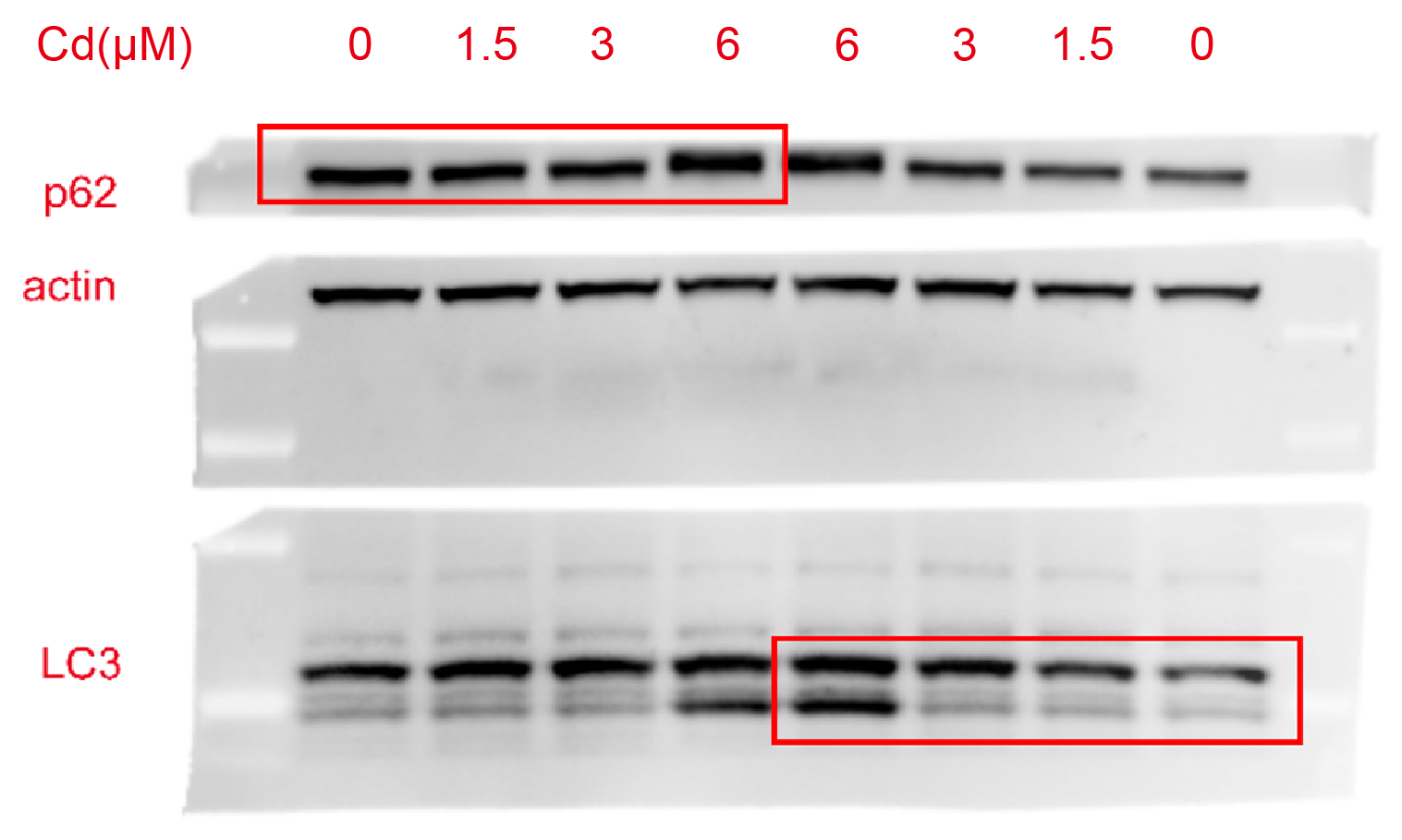


Fig.1K


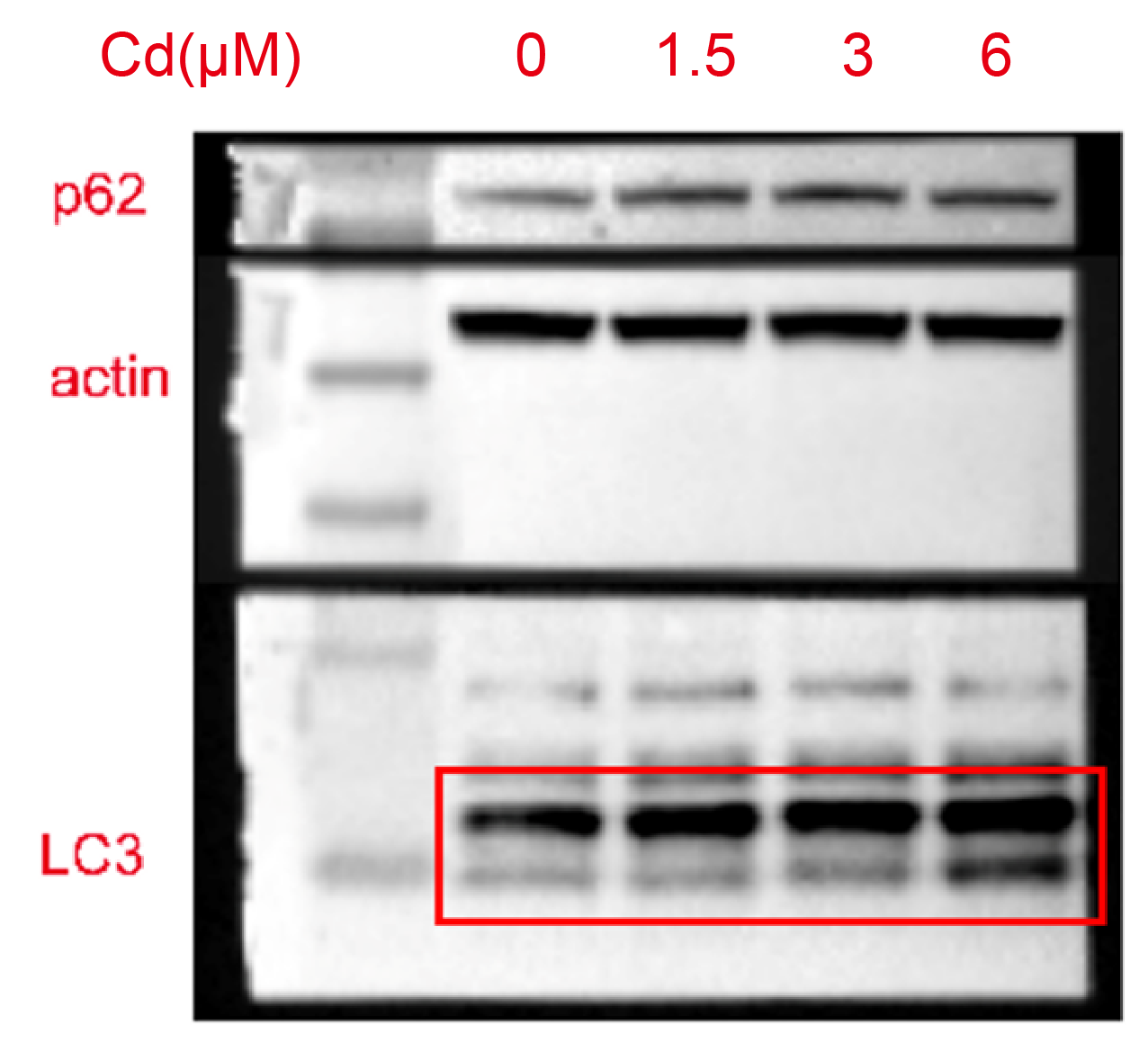


Fig.1M


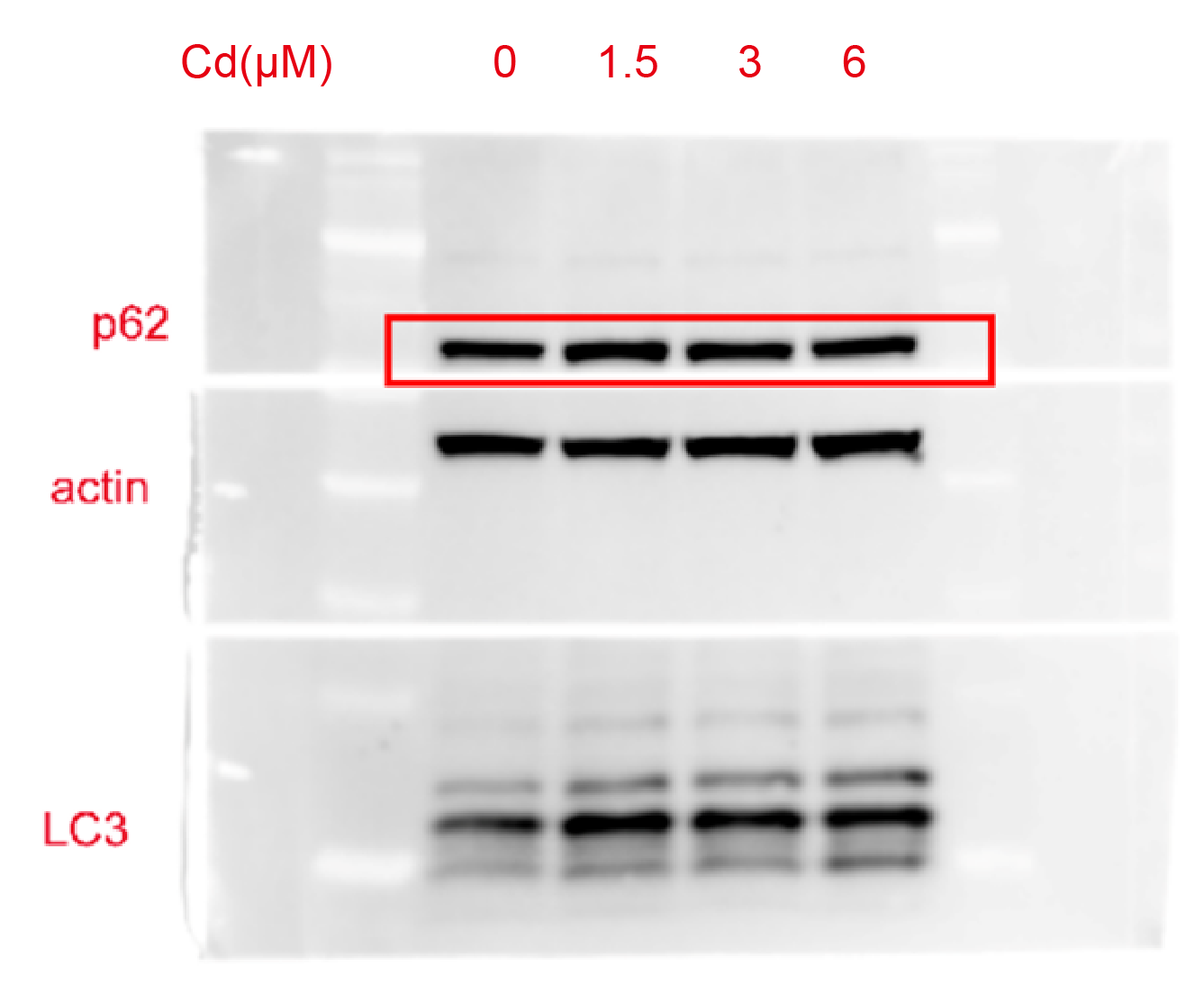


Fig.1N


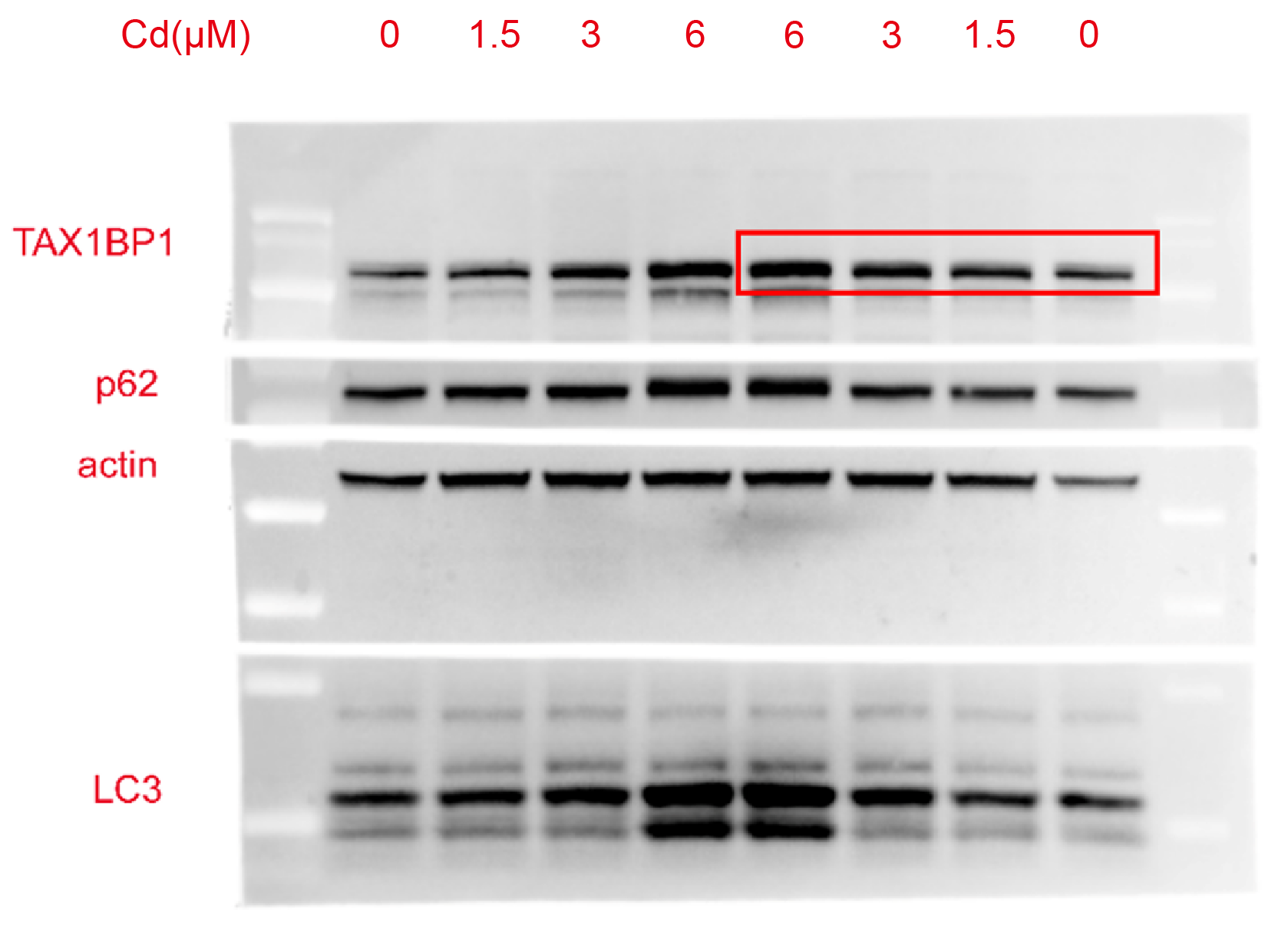


Fig.1O


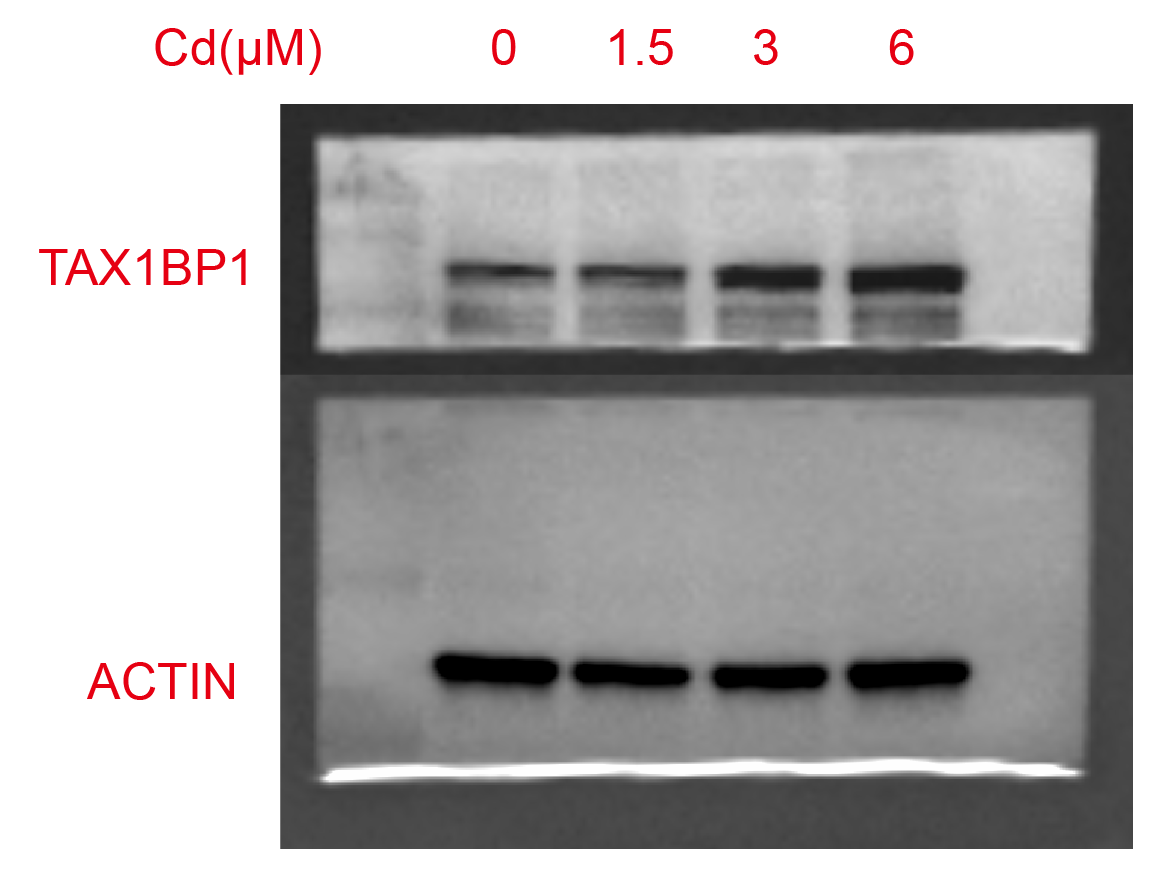


Fig.1P


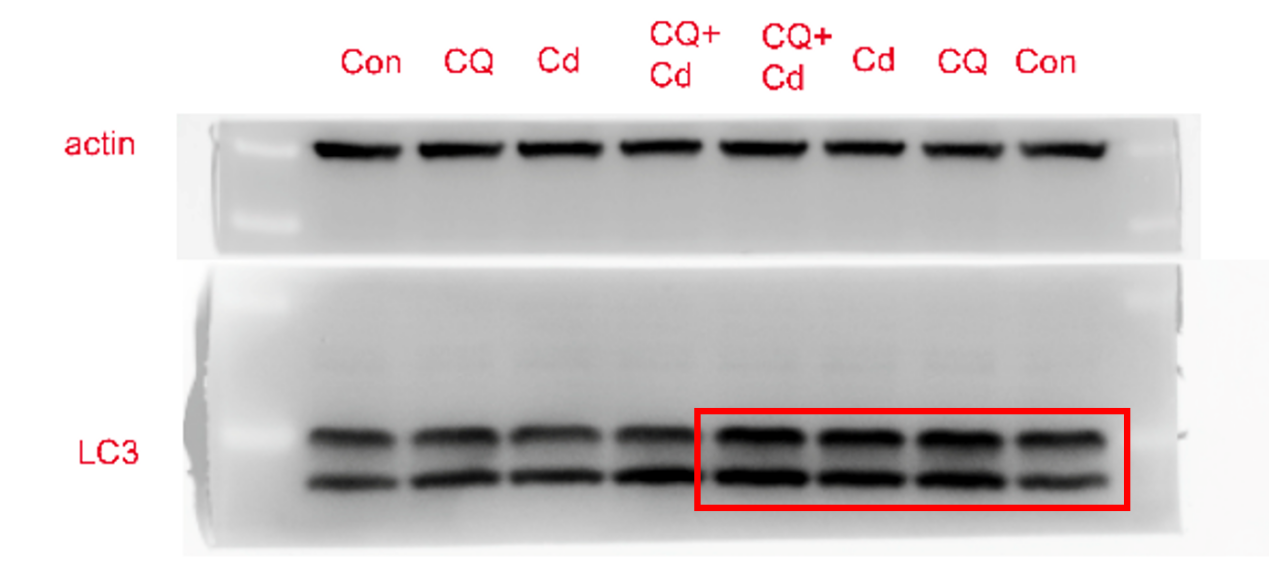


Fig.1Q


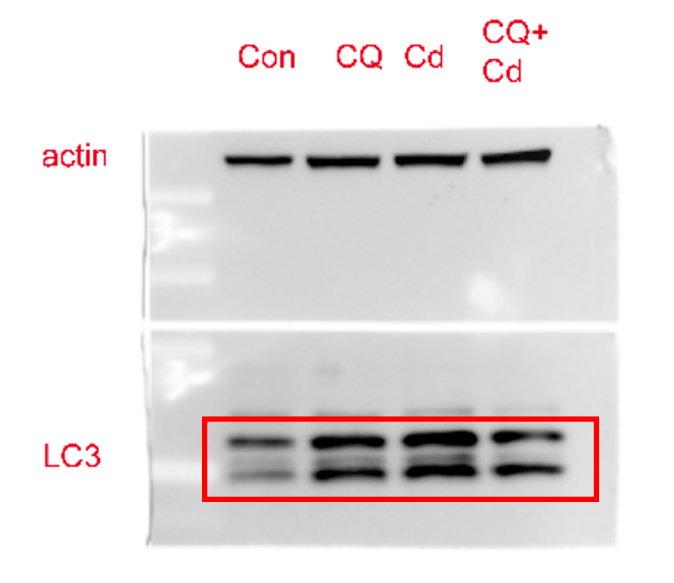


Fig.2D


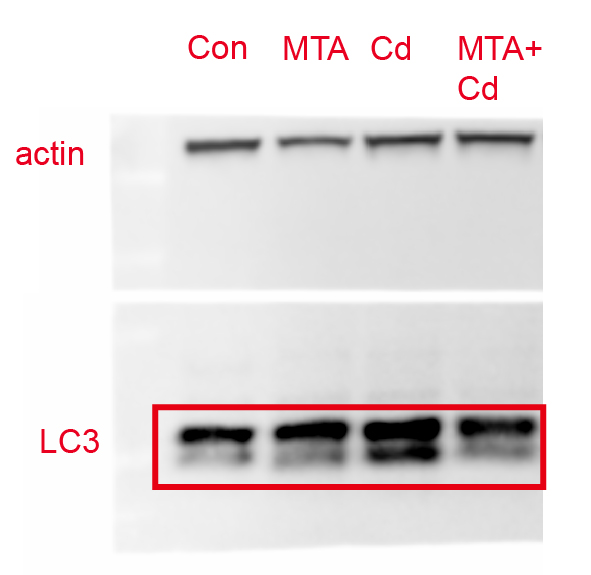


Fig.2E


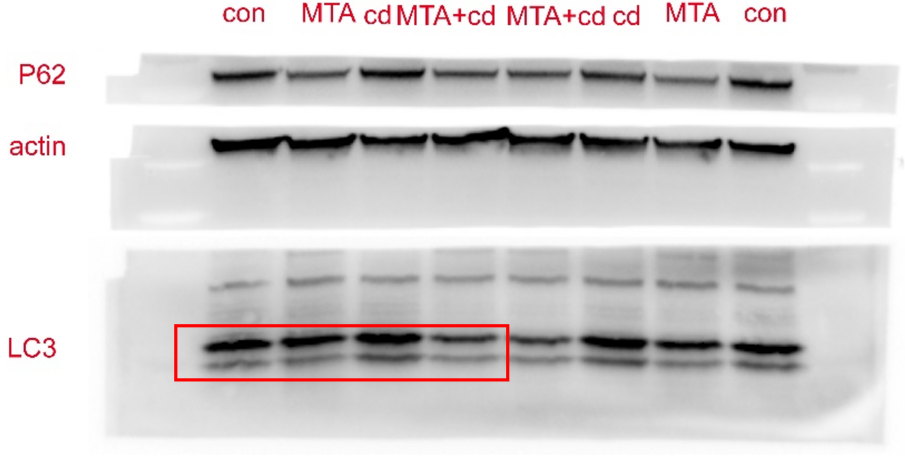


Fig.2F


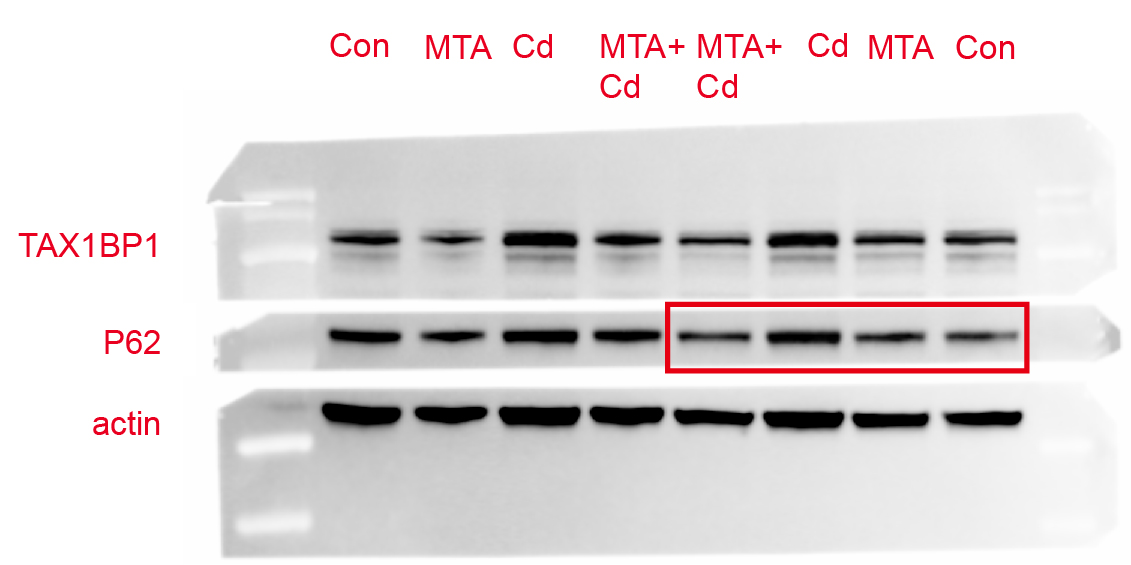


Fig.2G


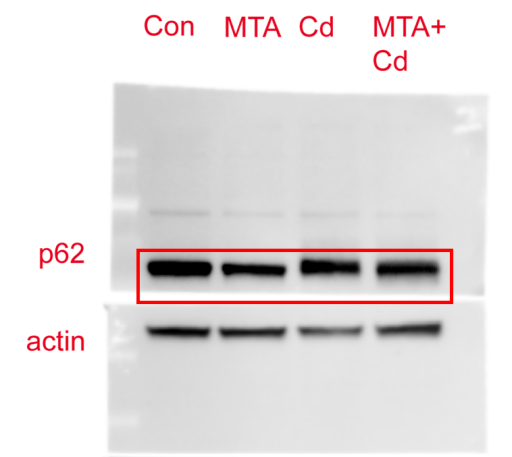


Fig.2H


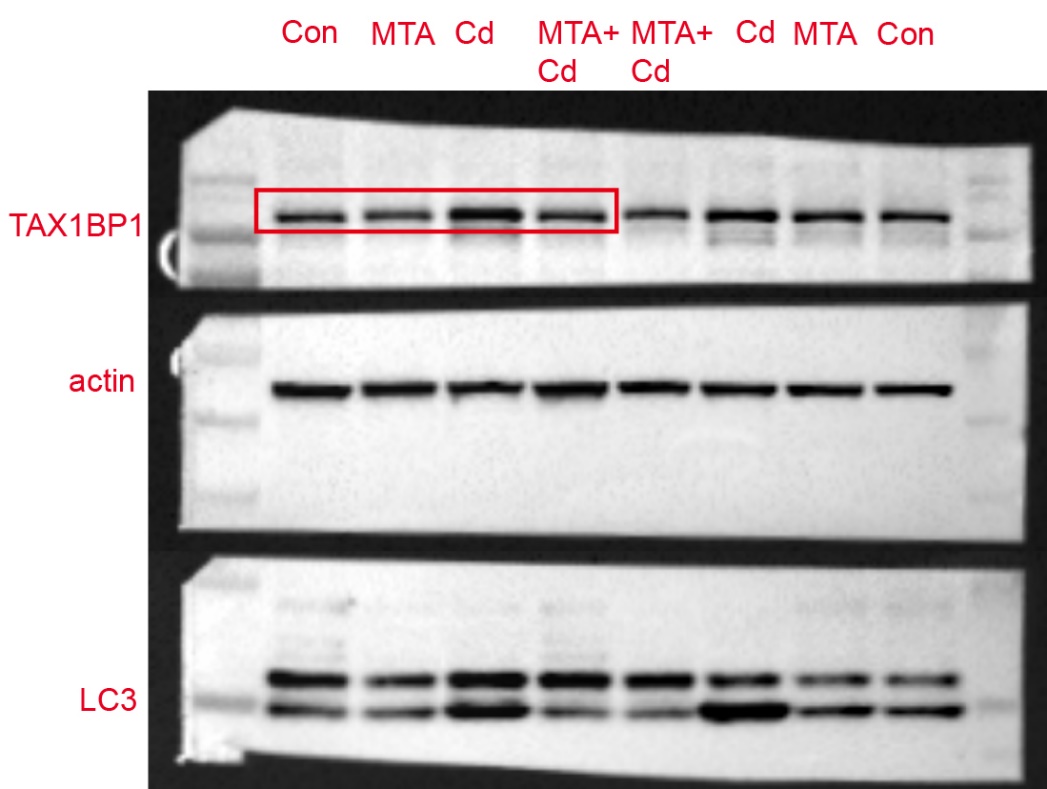


Fig.2I


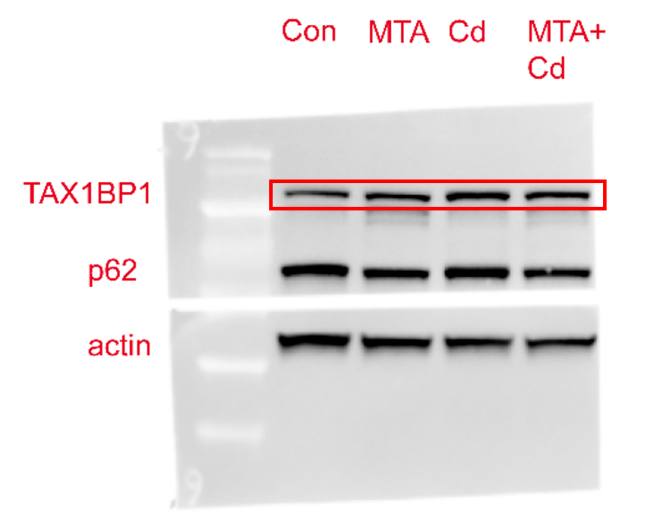


Fig.3D and Fig.3E


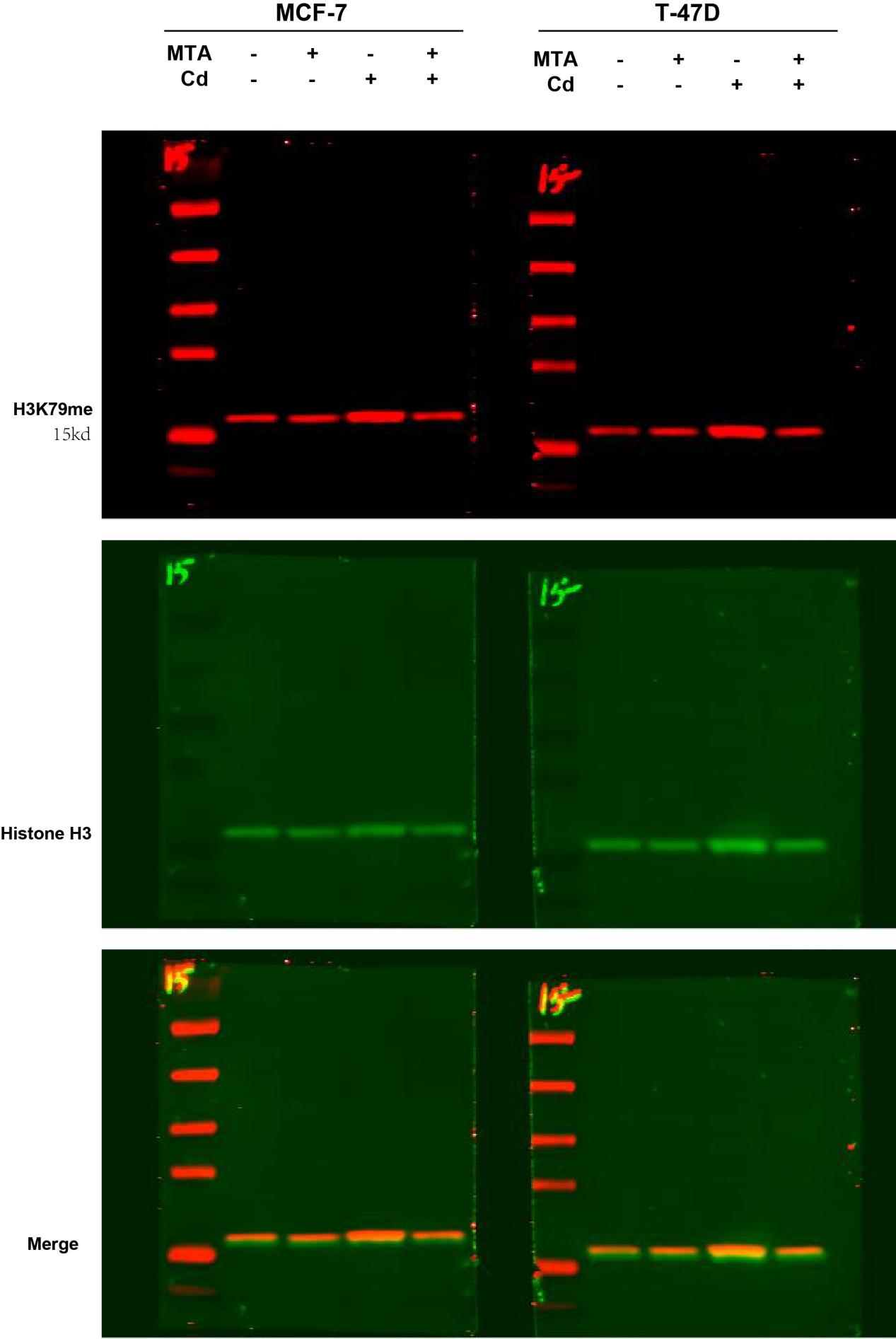


Fig.3F and Fig.3G


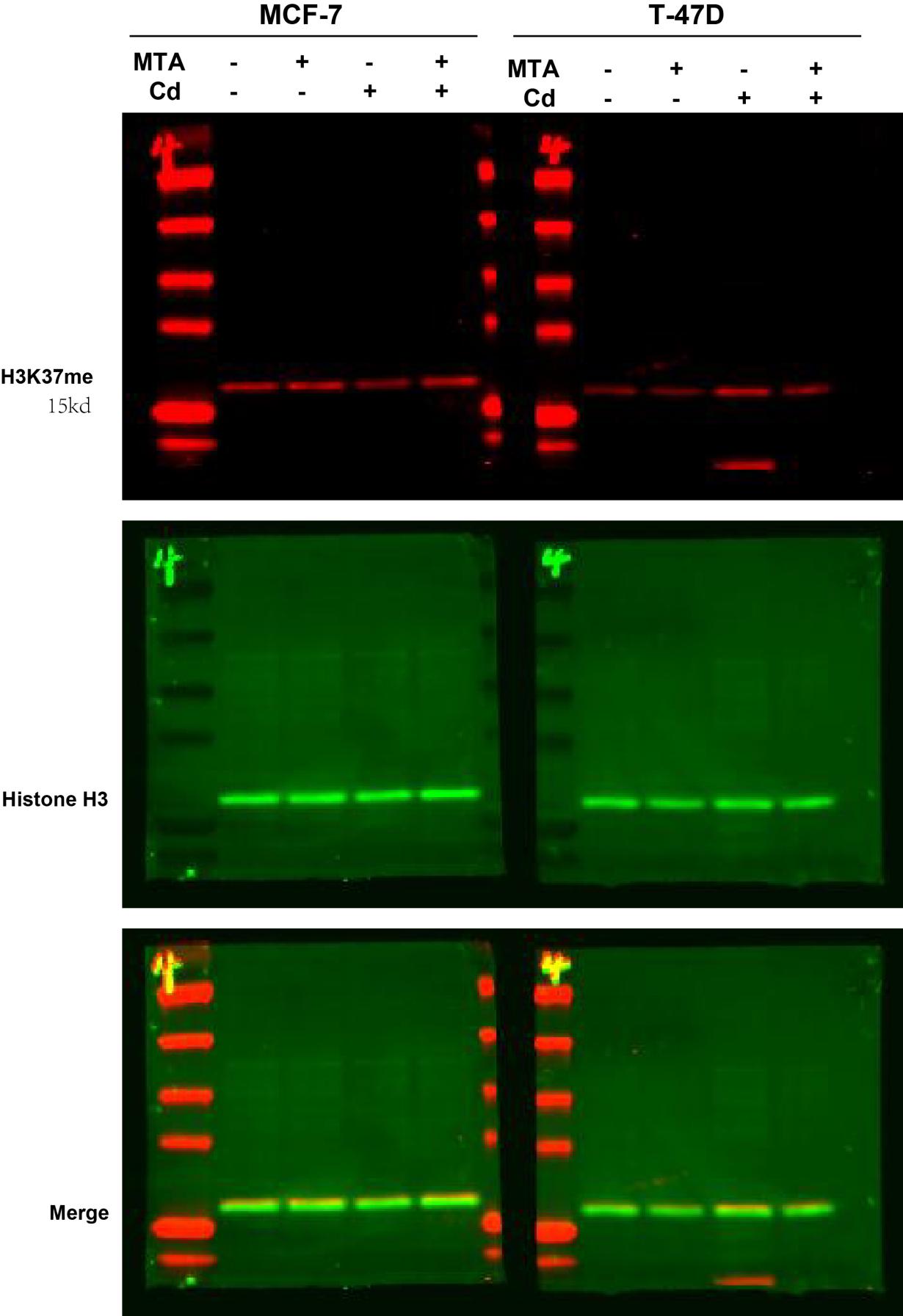


Fig.3H and Fig.3I


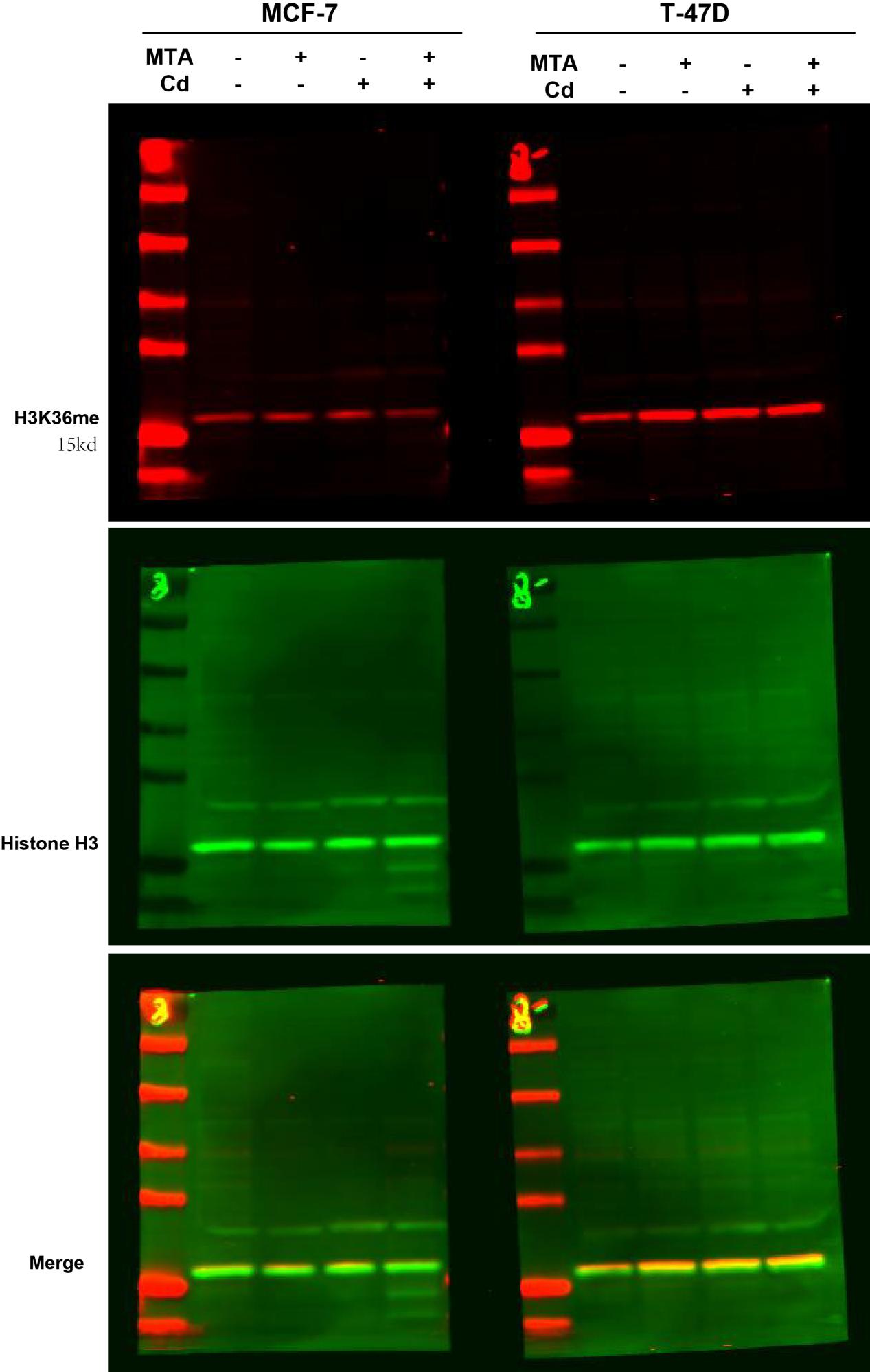


Fig.3J and Fig.3K


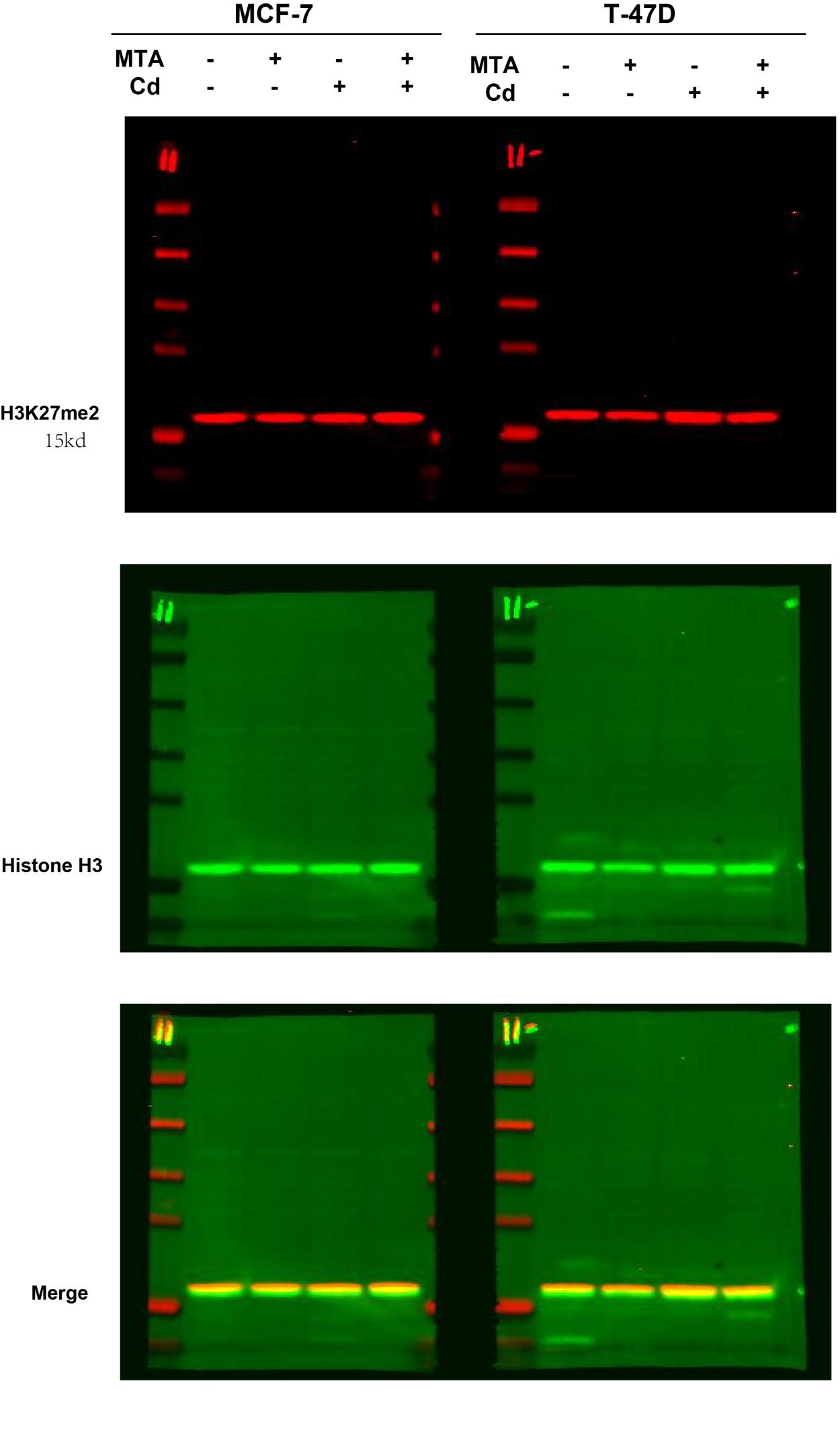


Fig.3L and Fig.3M


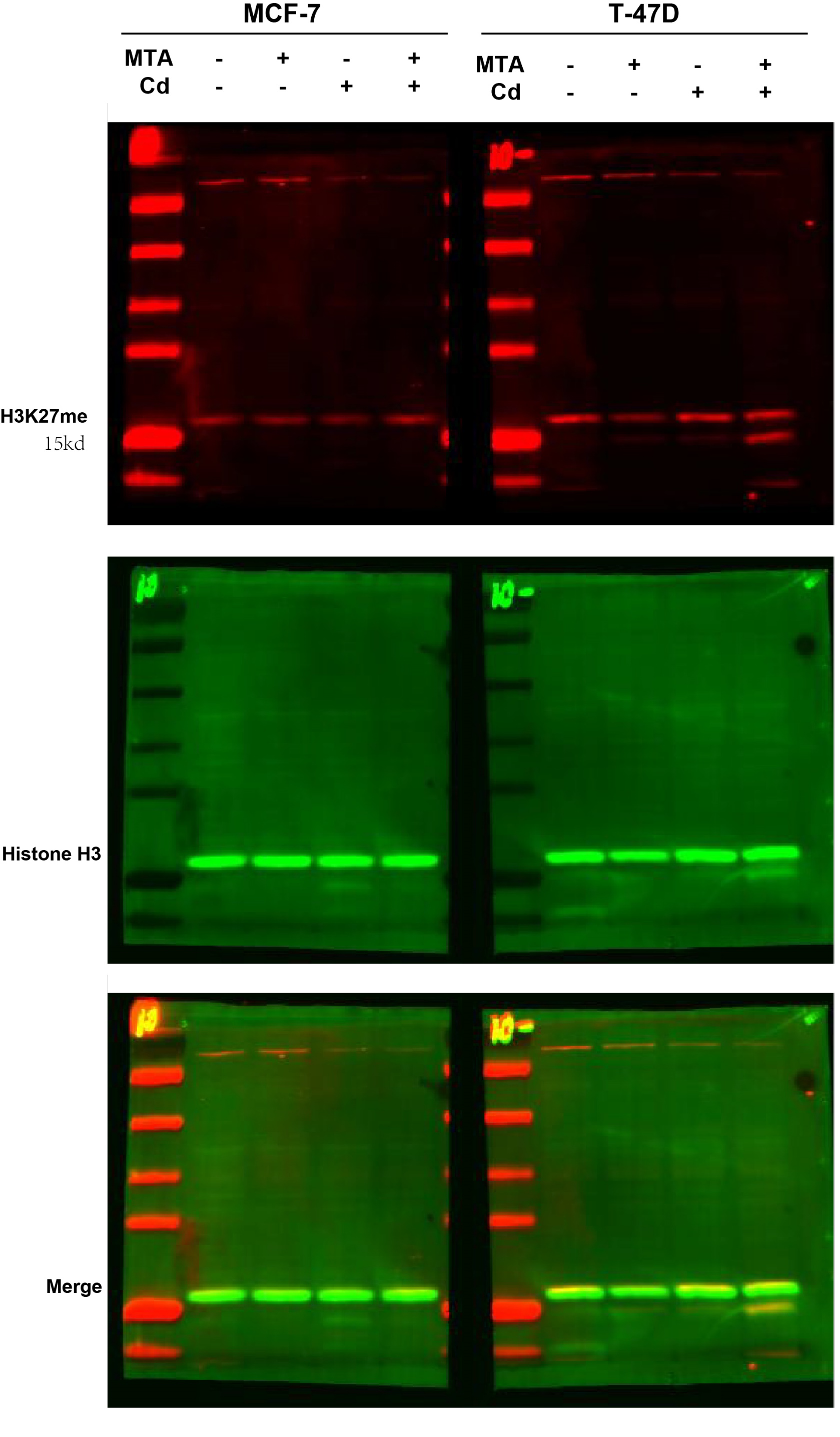


Fig.3N


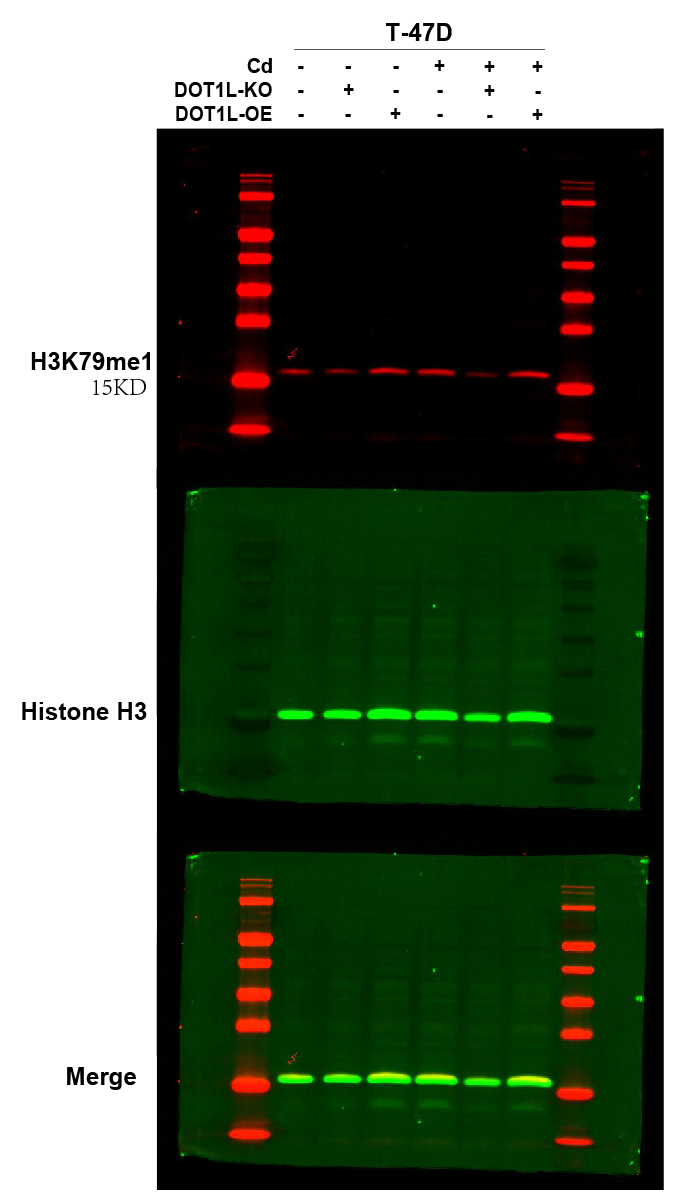


Fig.3O


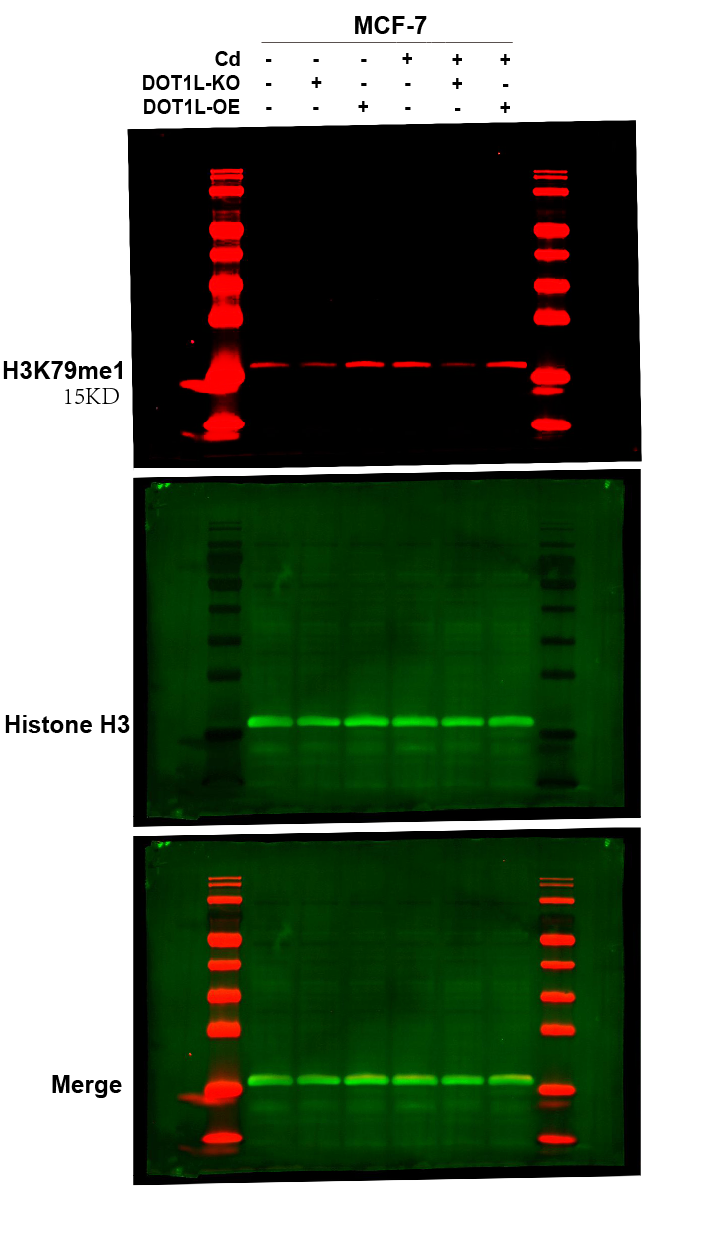


Fig.5A


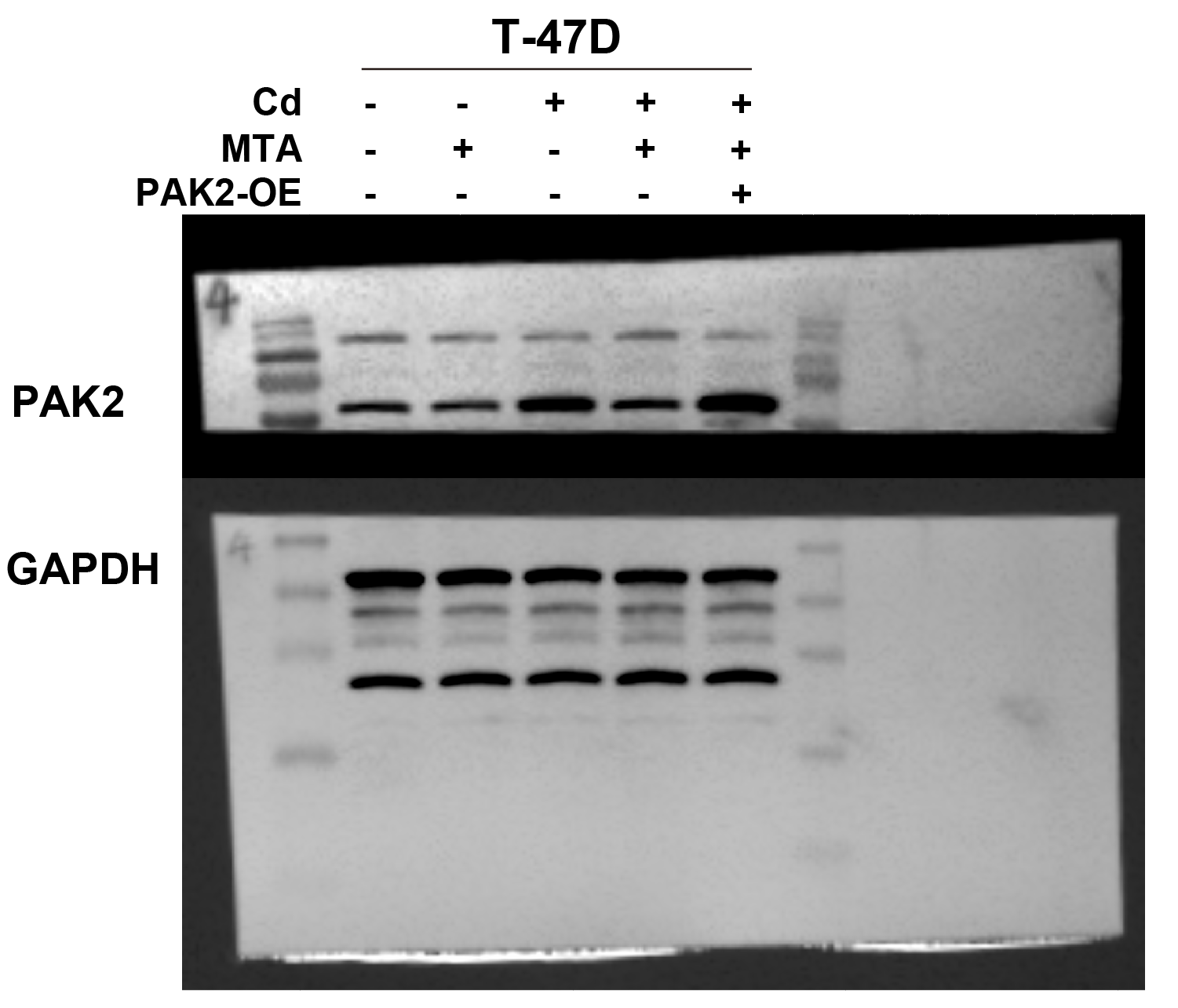


Fig.5B


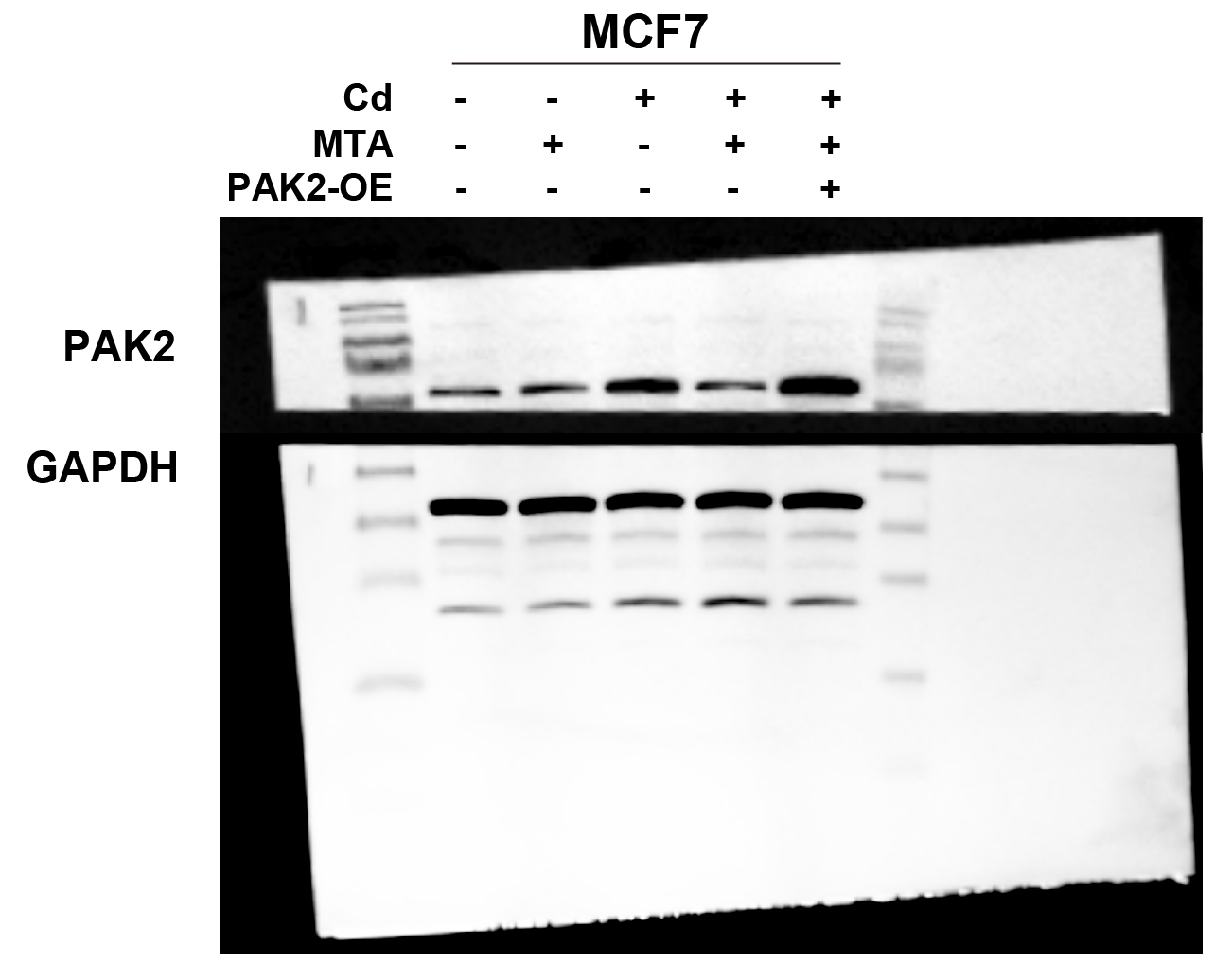


Fig.5C


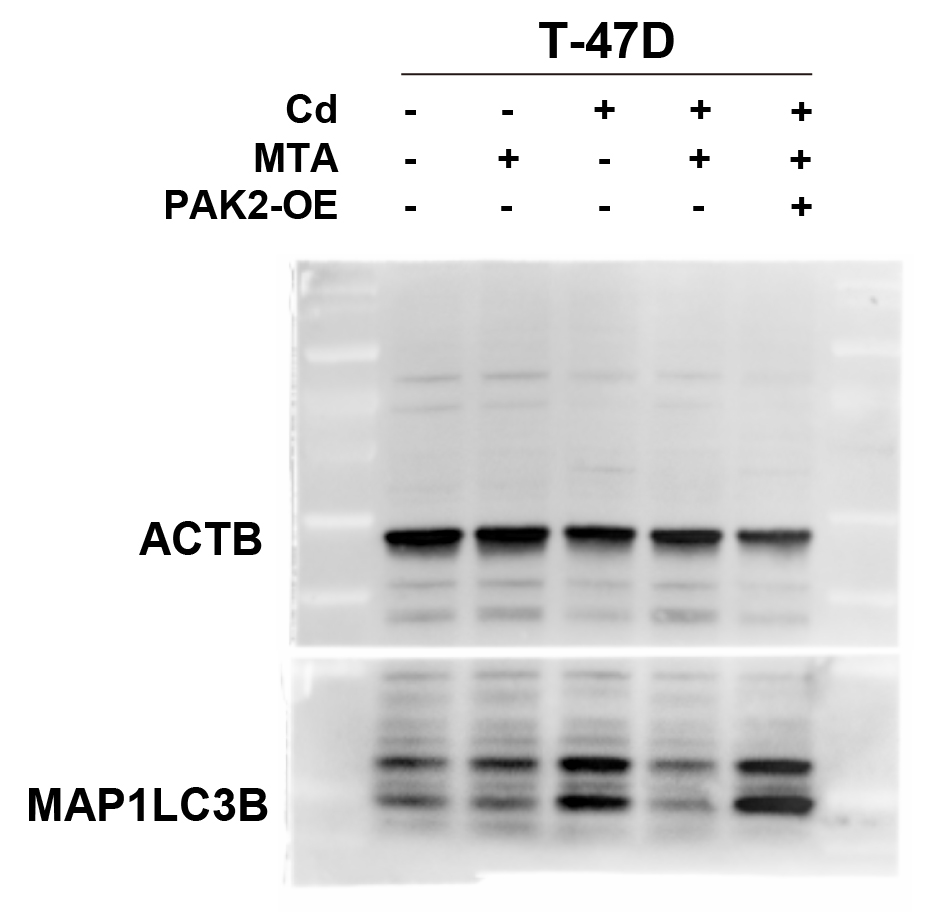


Fig.5D


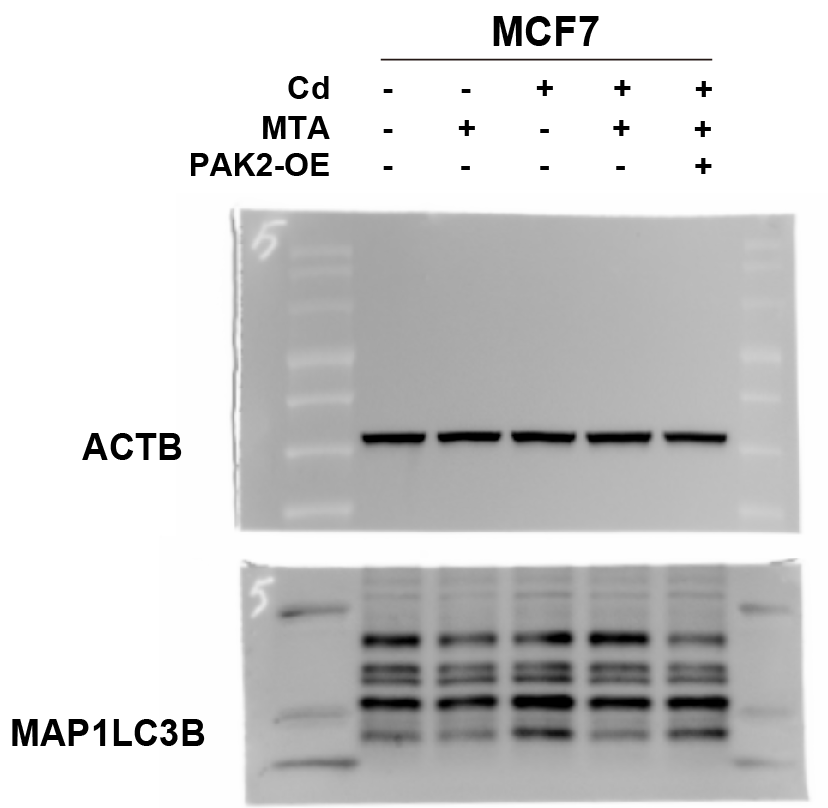


Fig.5E


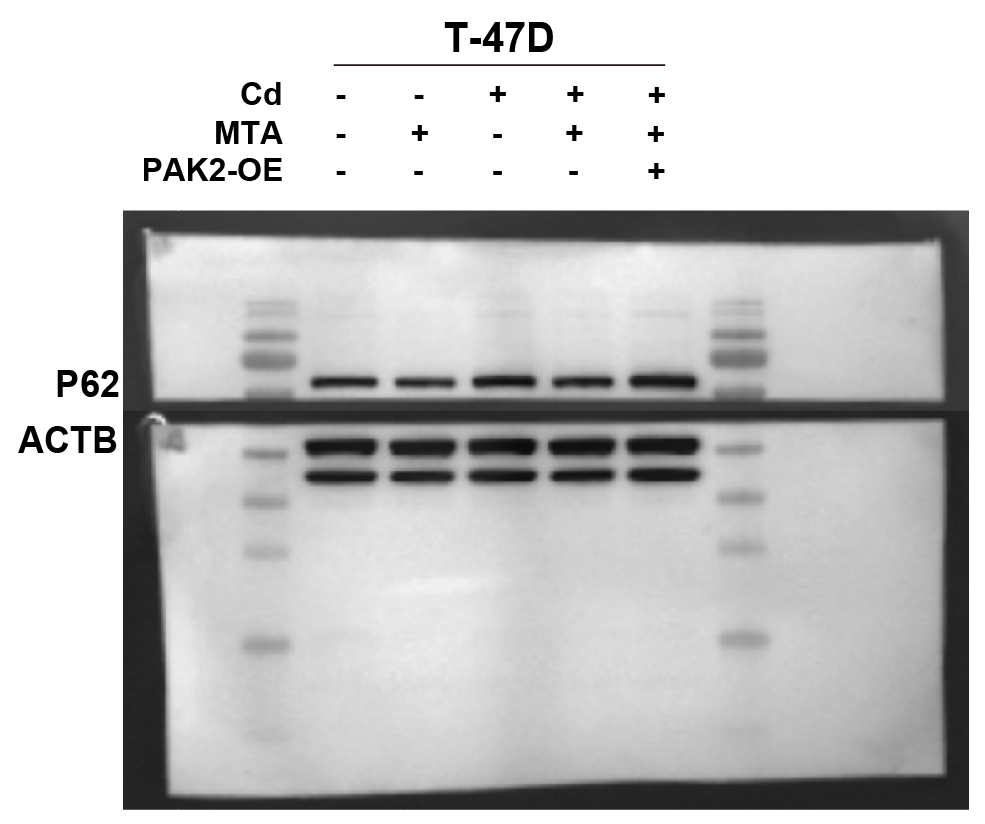


Fig.5F


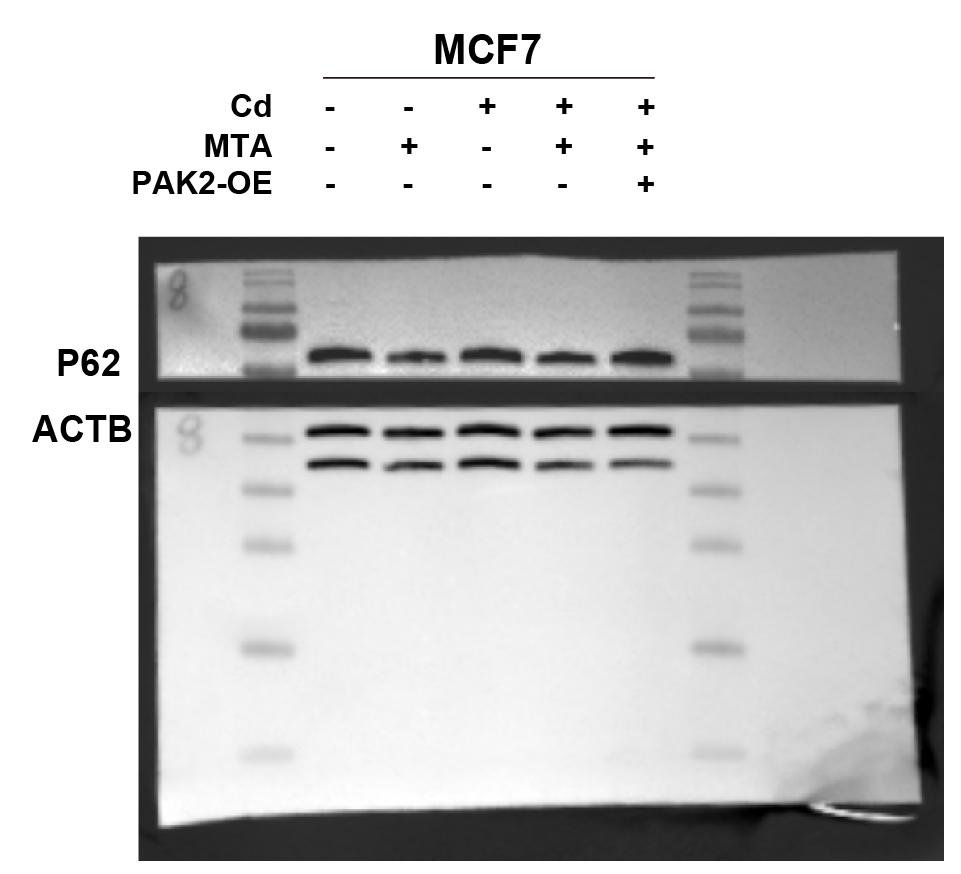


Fig.5G


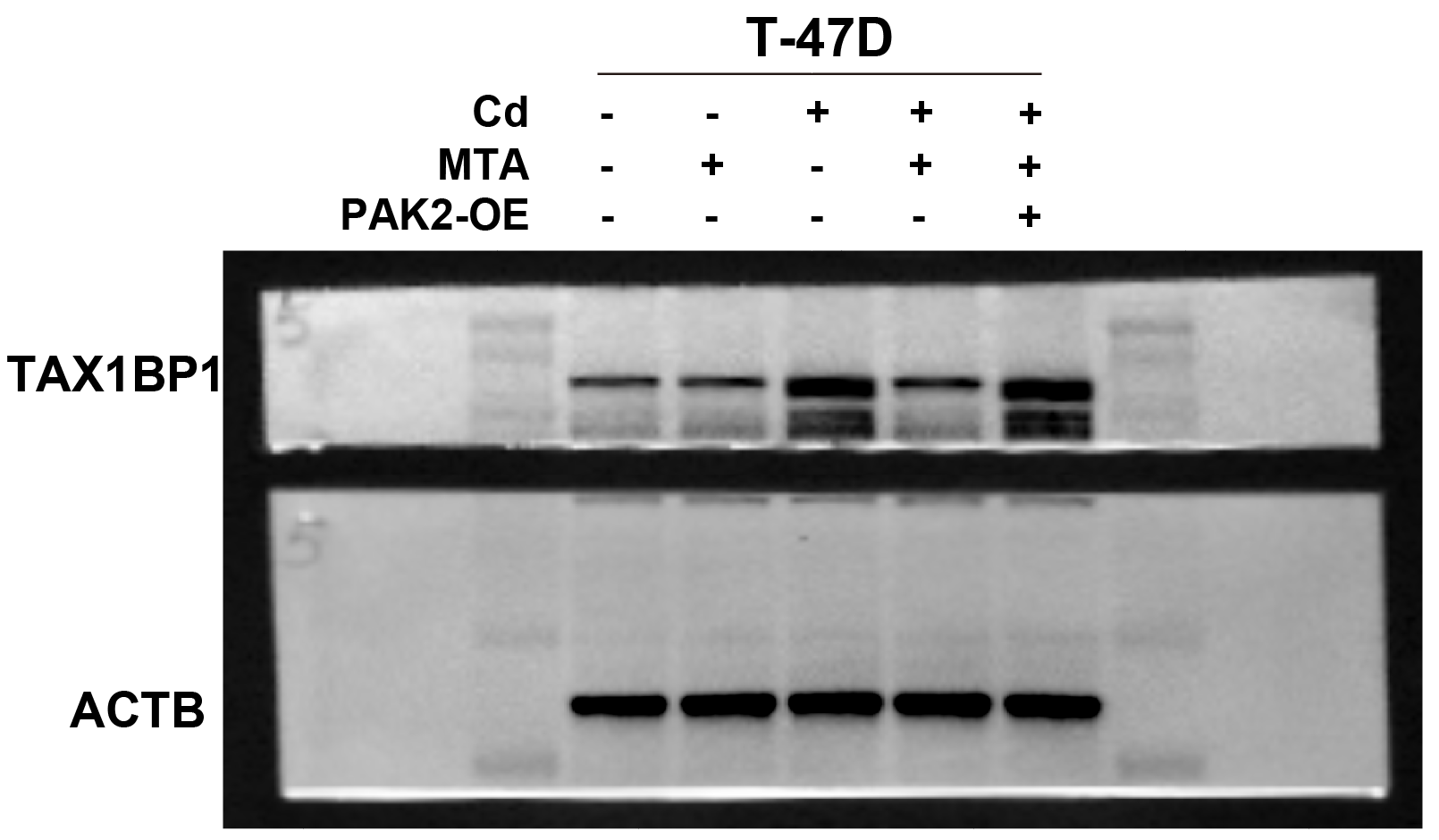


Fig.5H


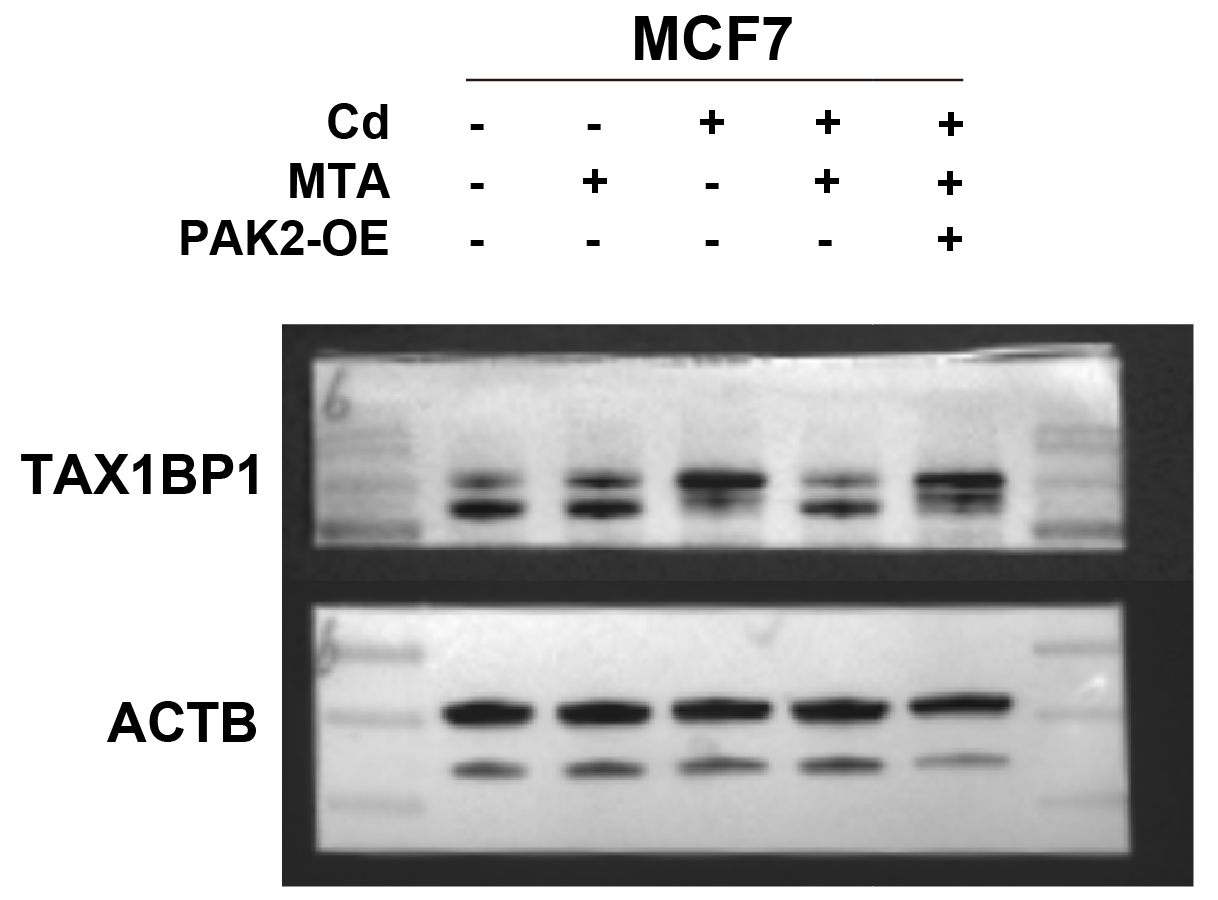


Fig.7A


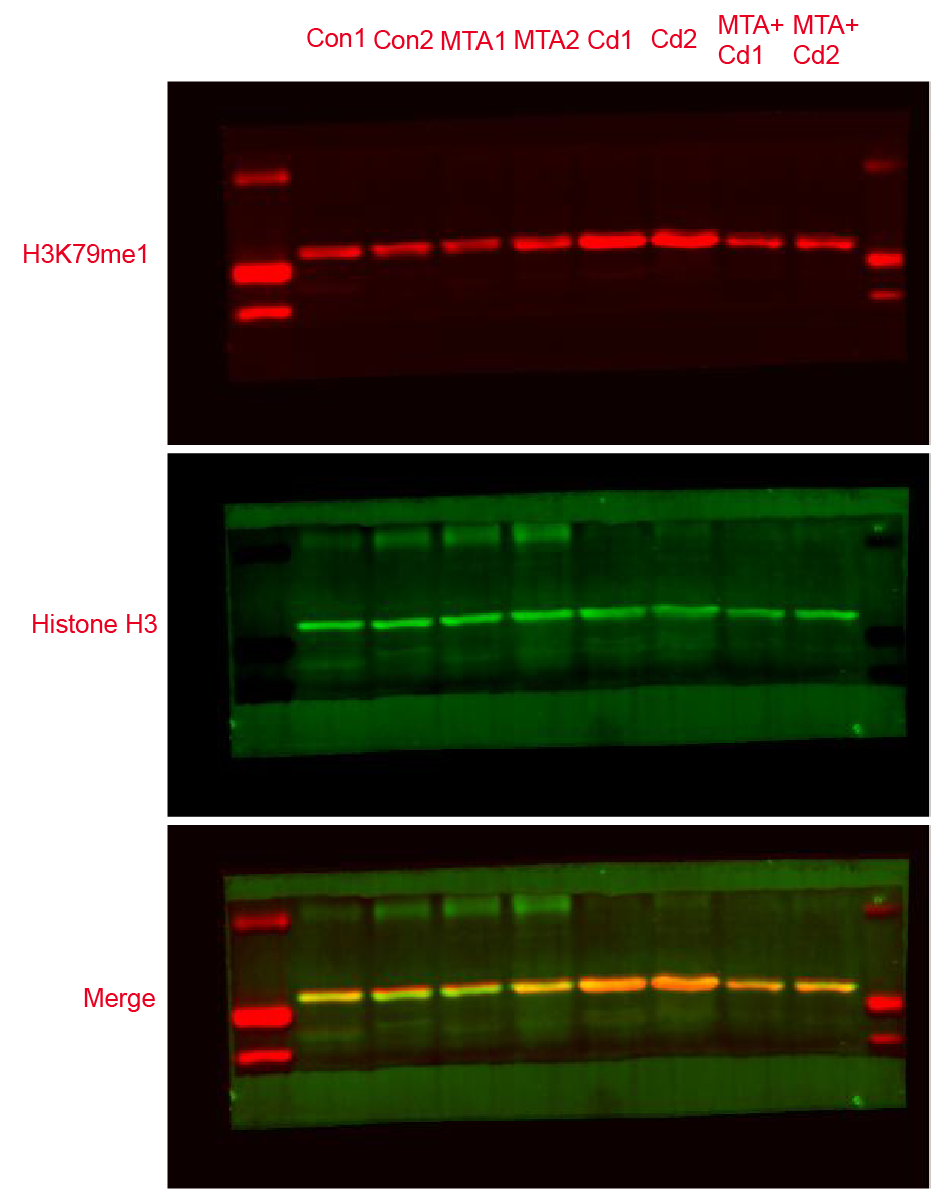


Fig.7C


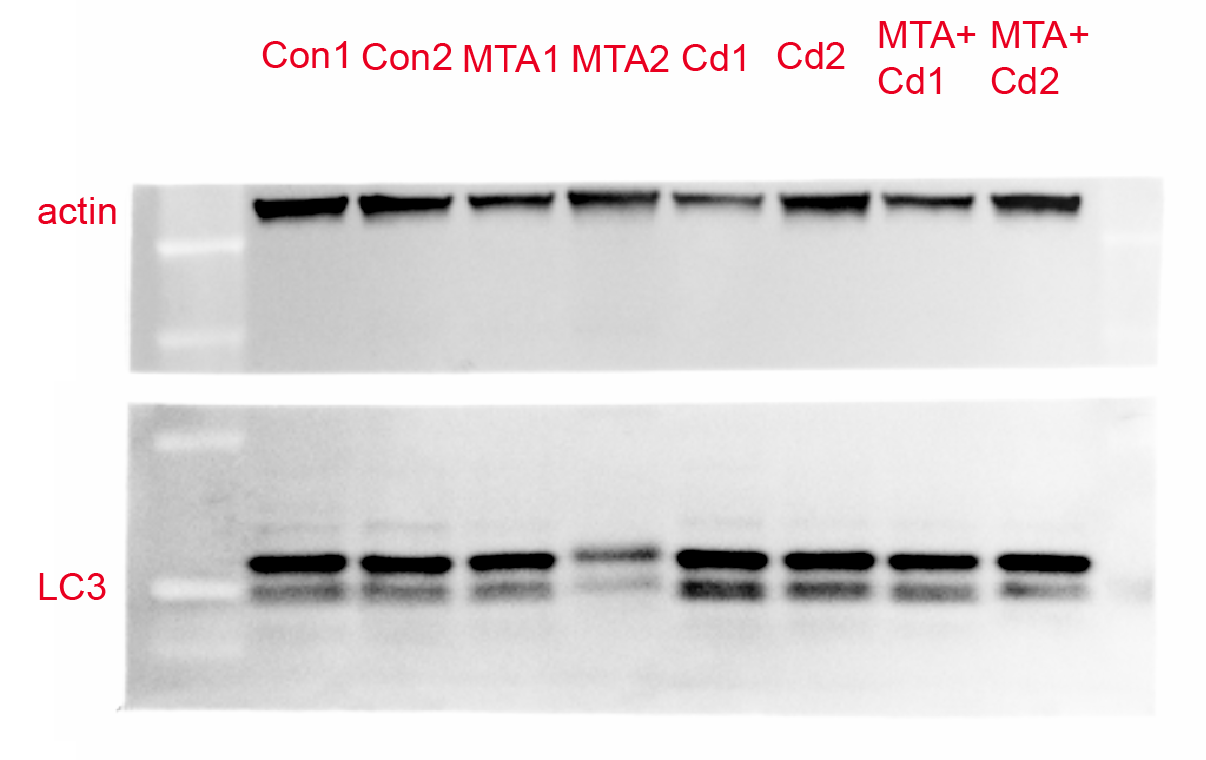


Fig.7D


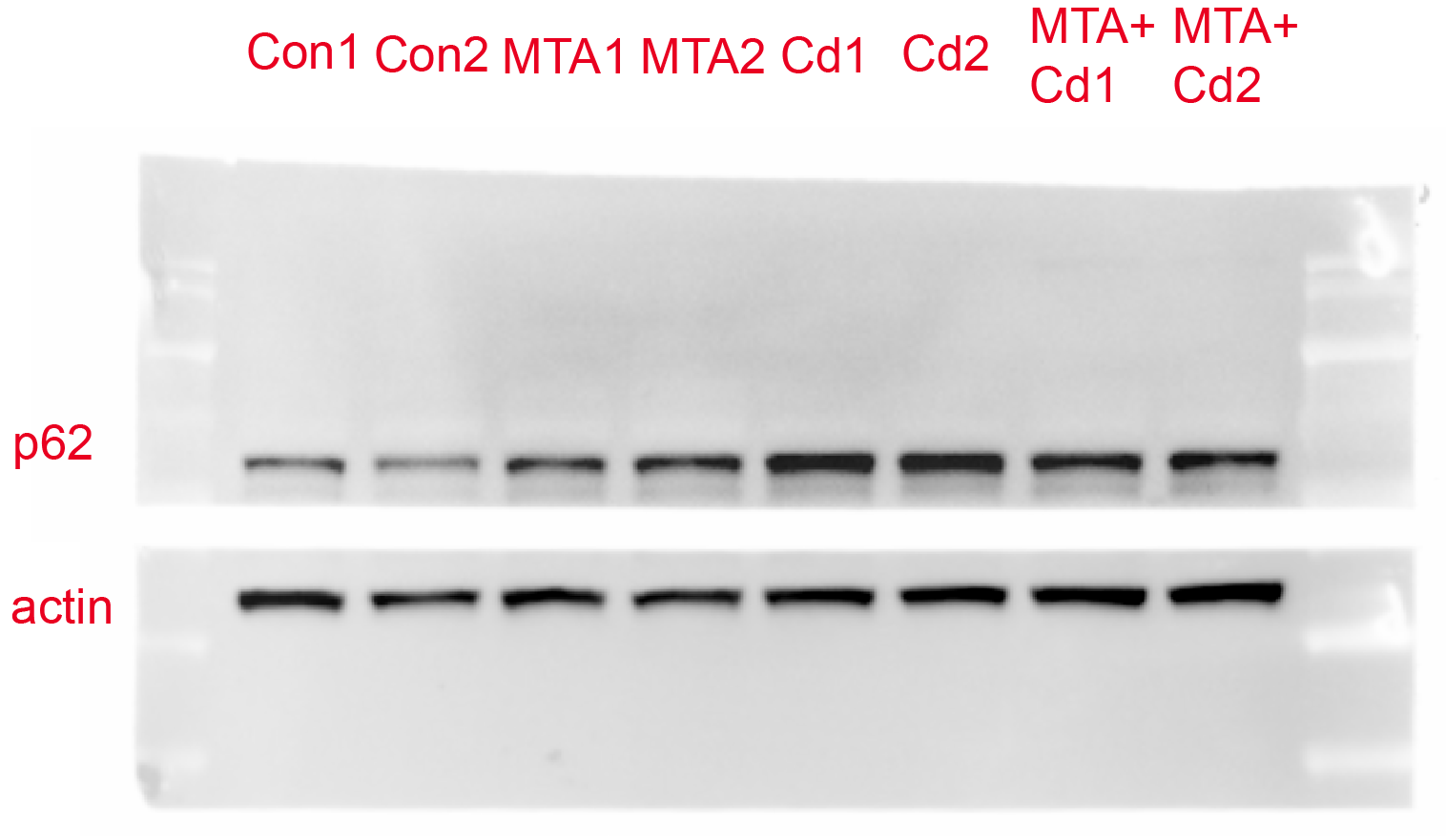


Fig.S2A


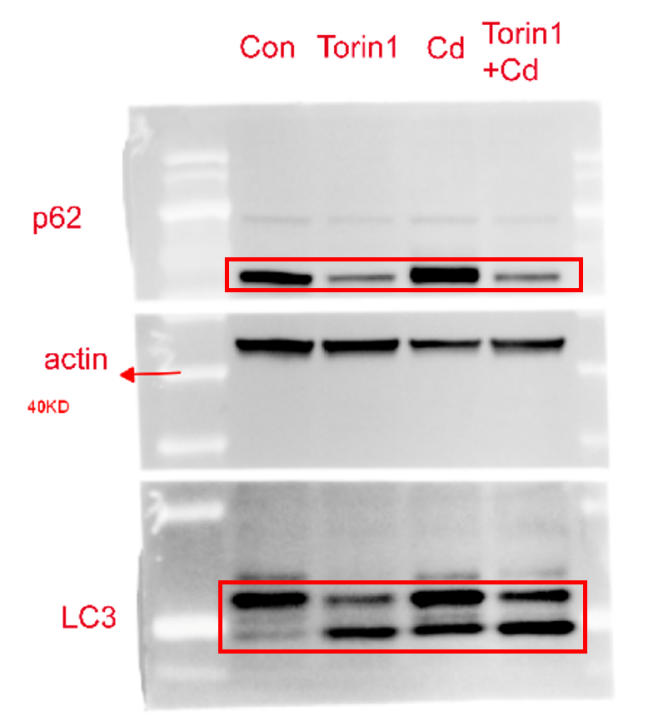


Fig.S2D


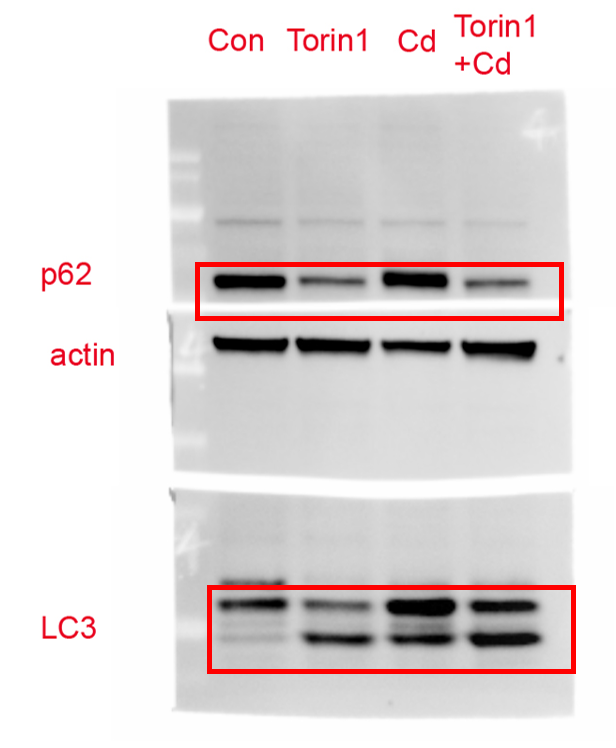


Fig.S8


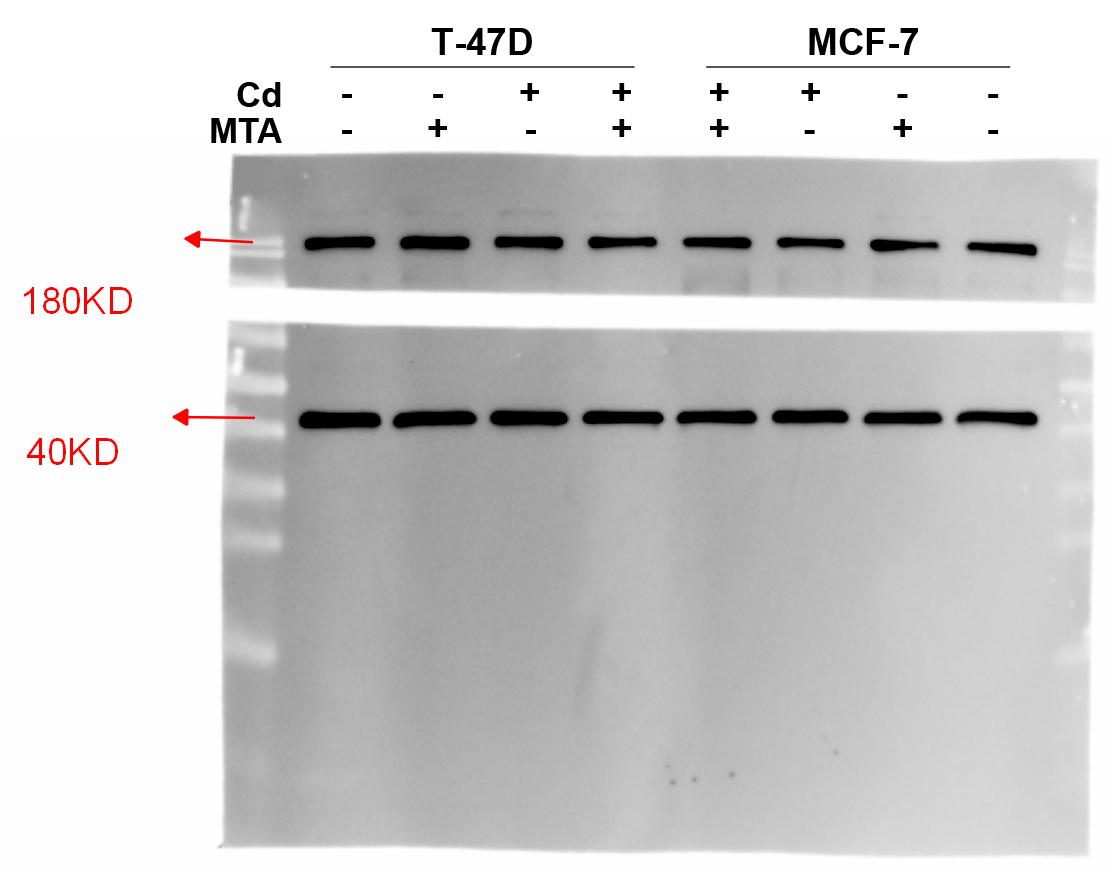


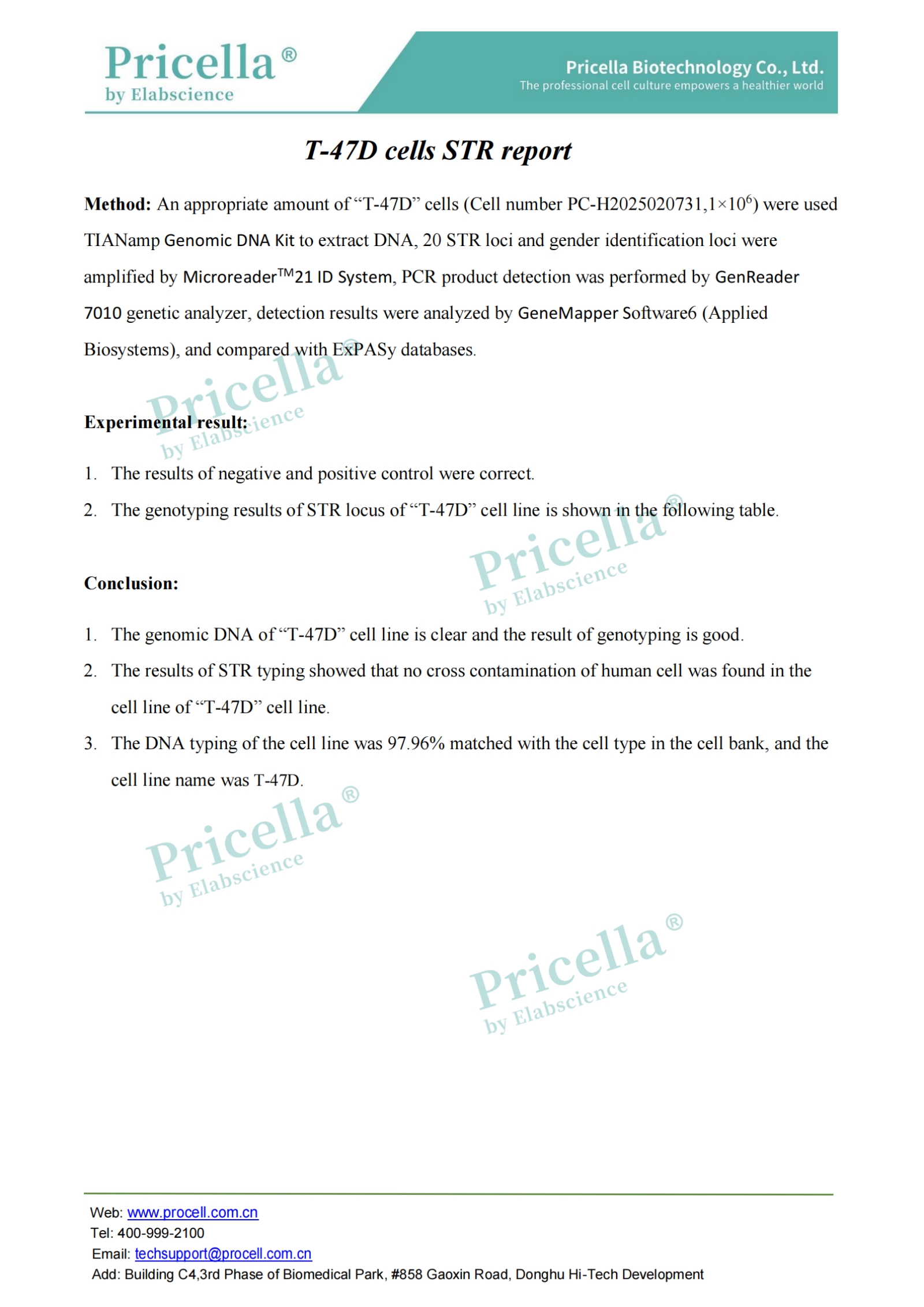


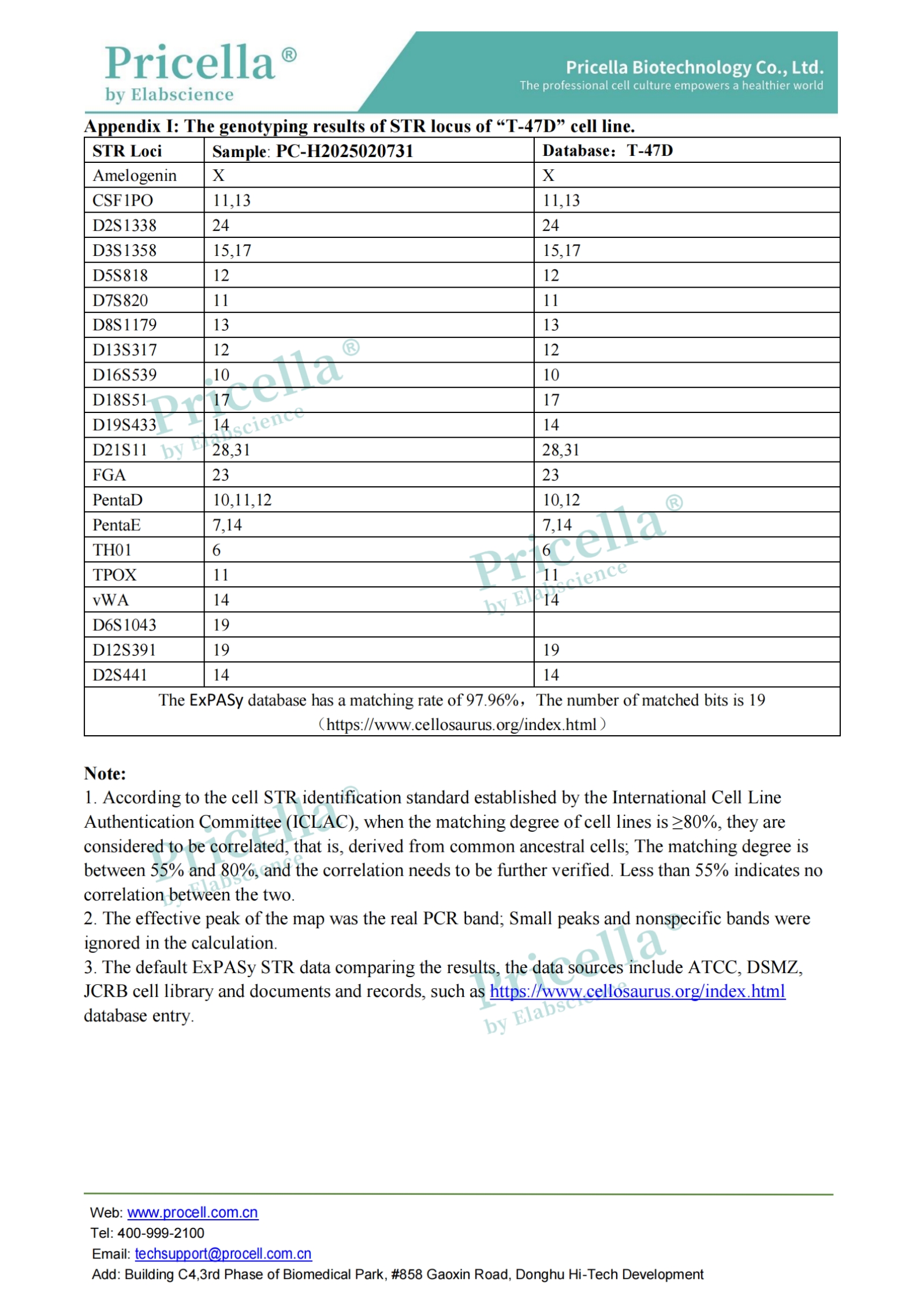


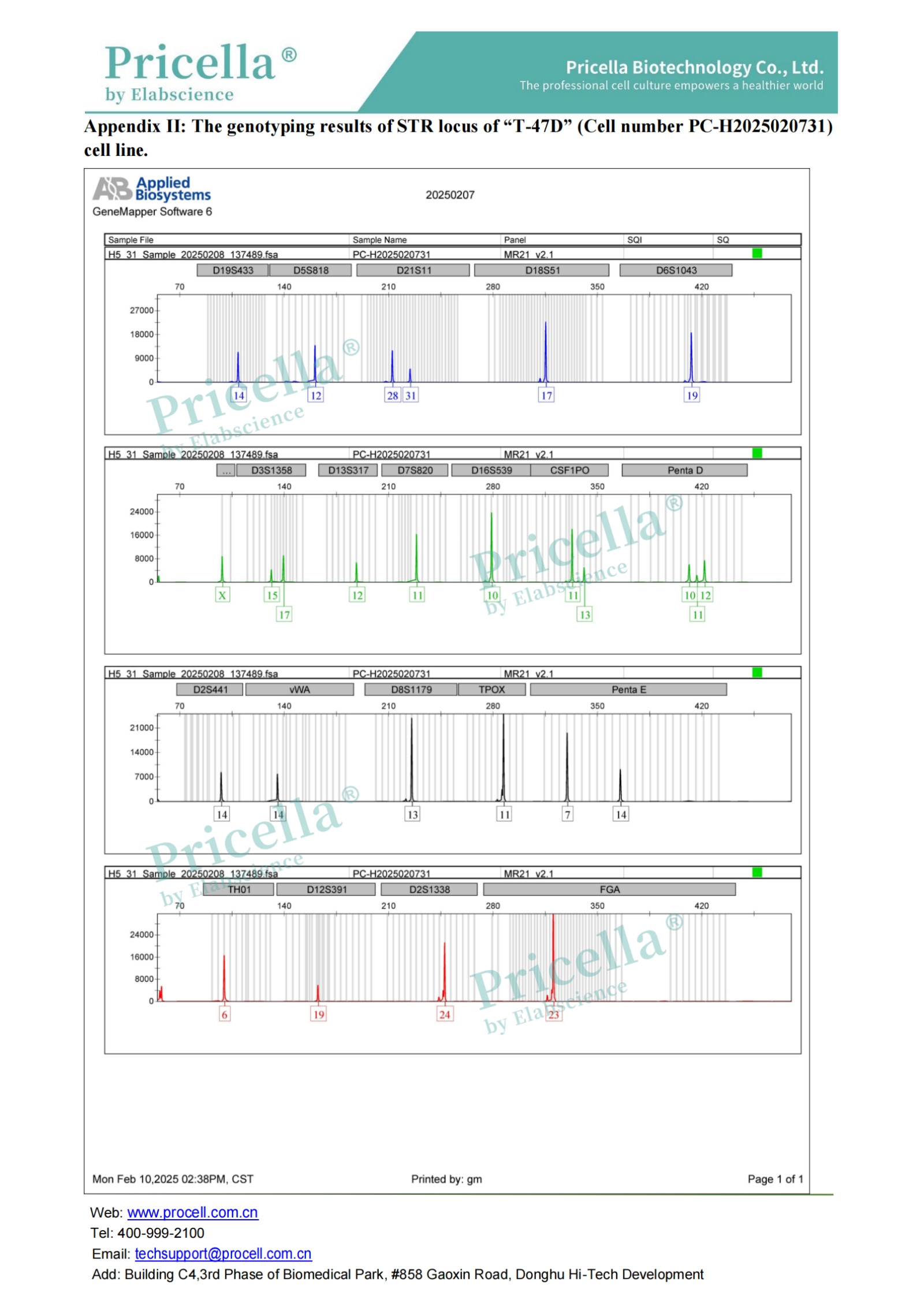


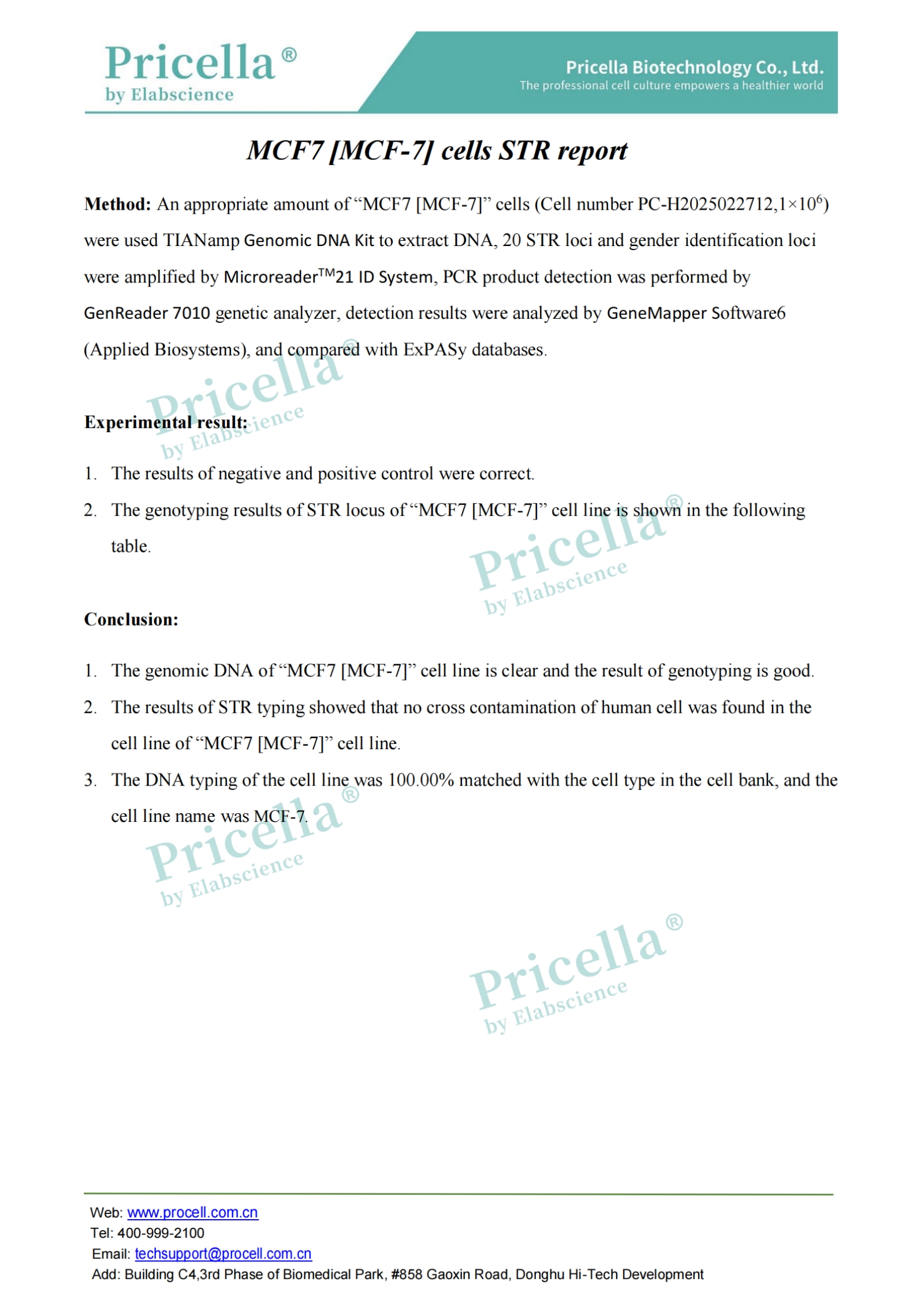


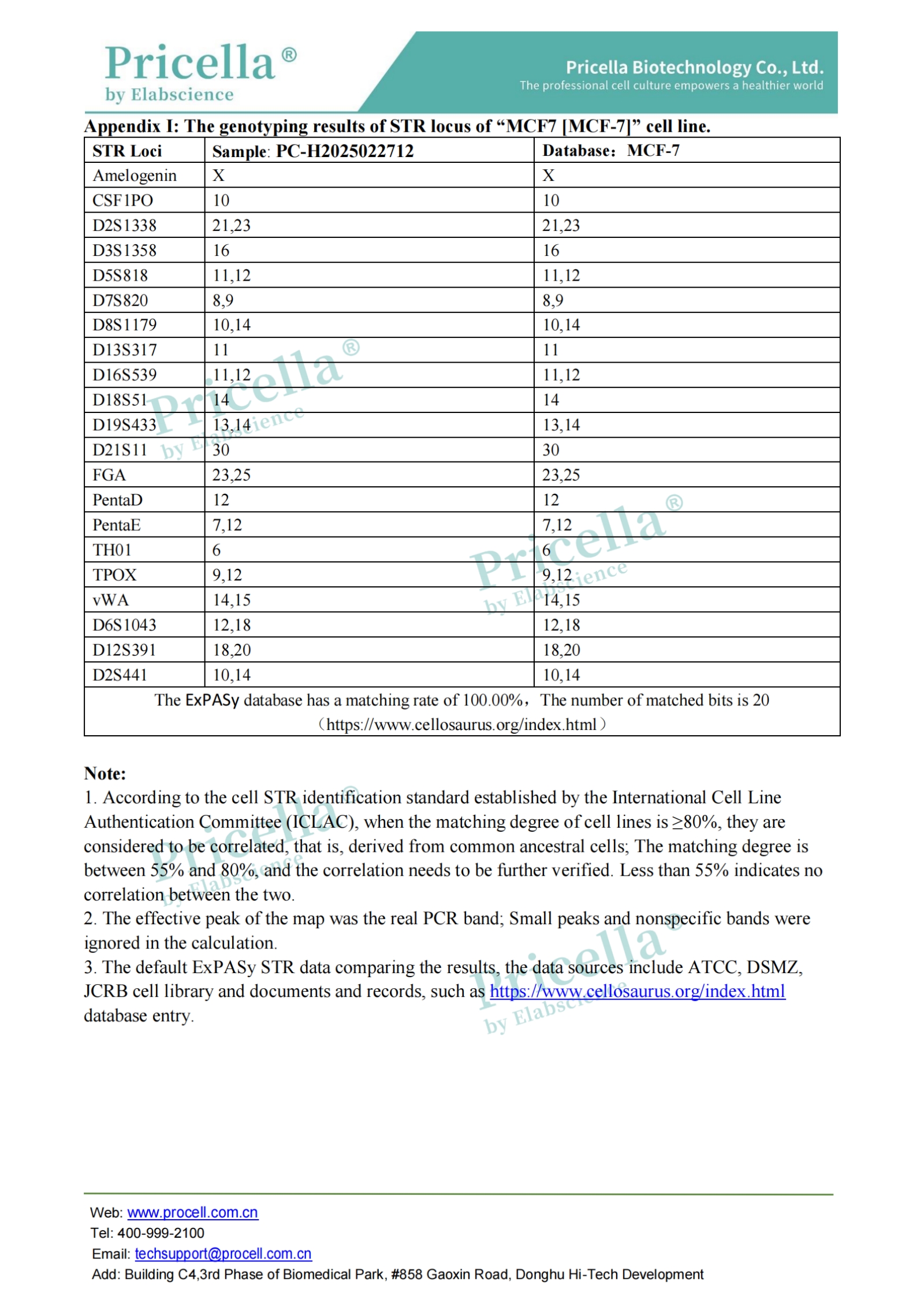


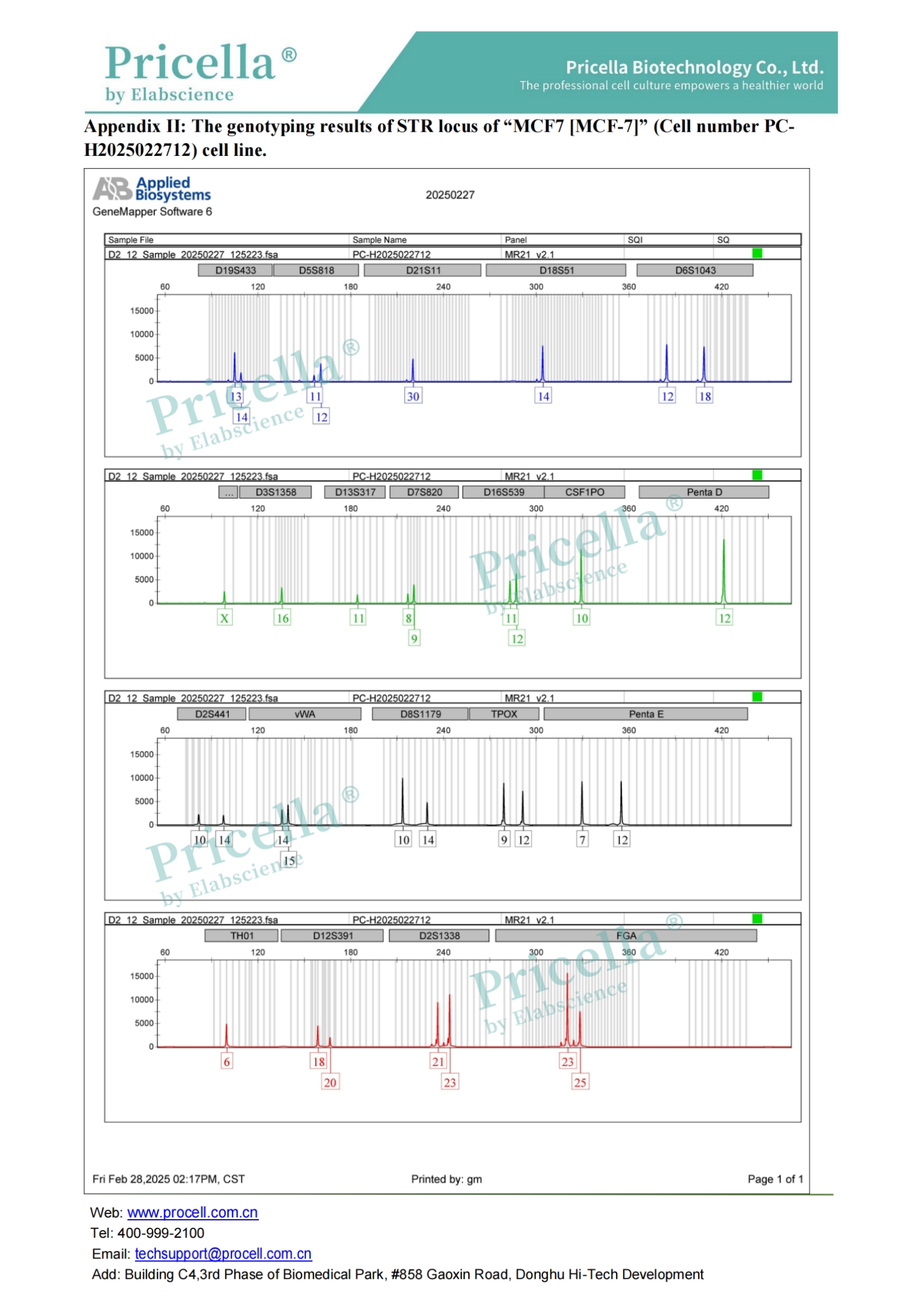


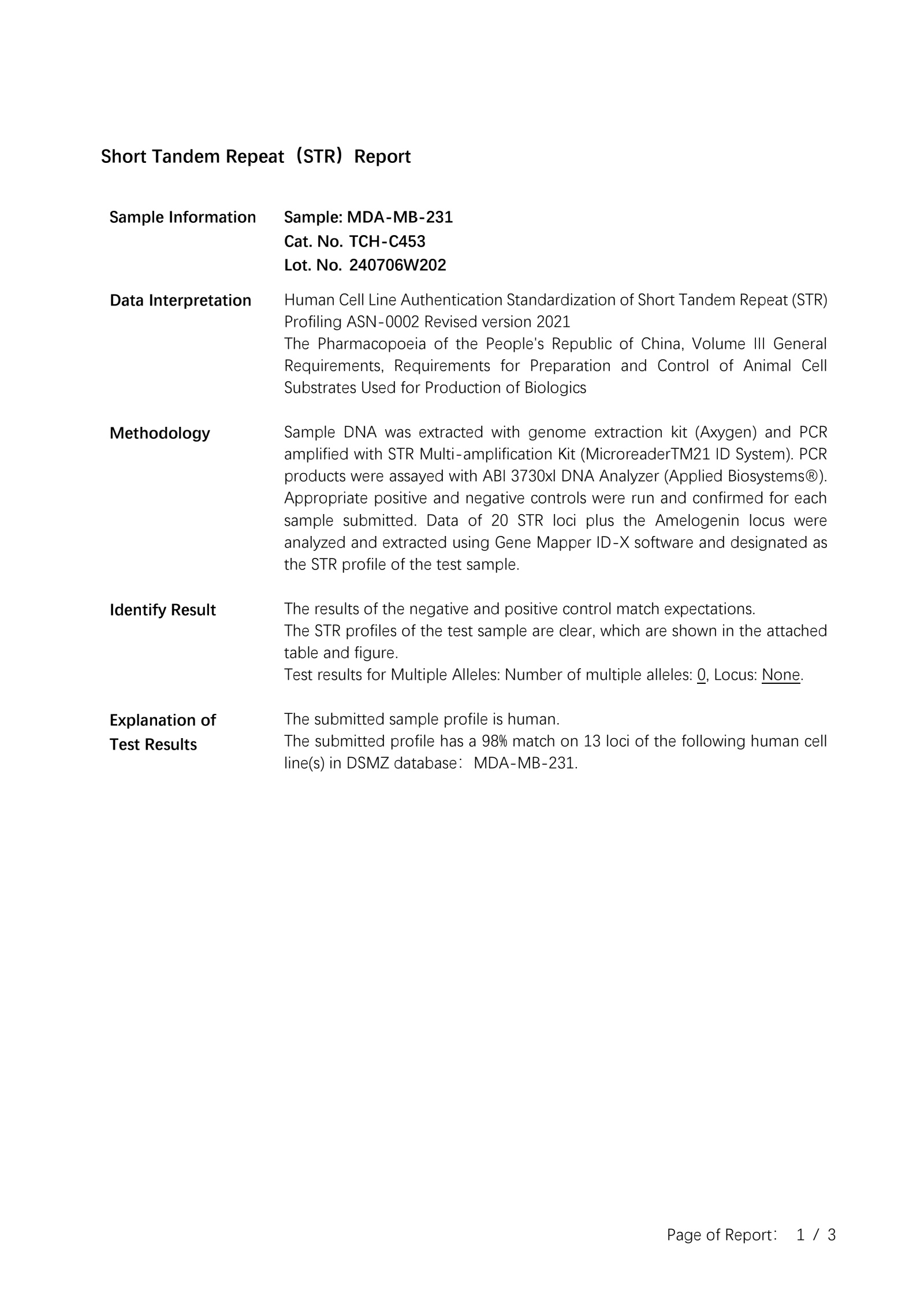


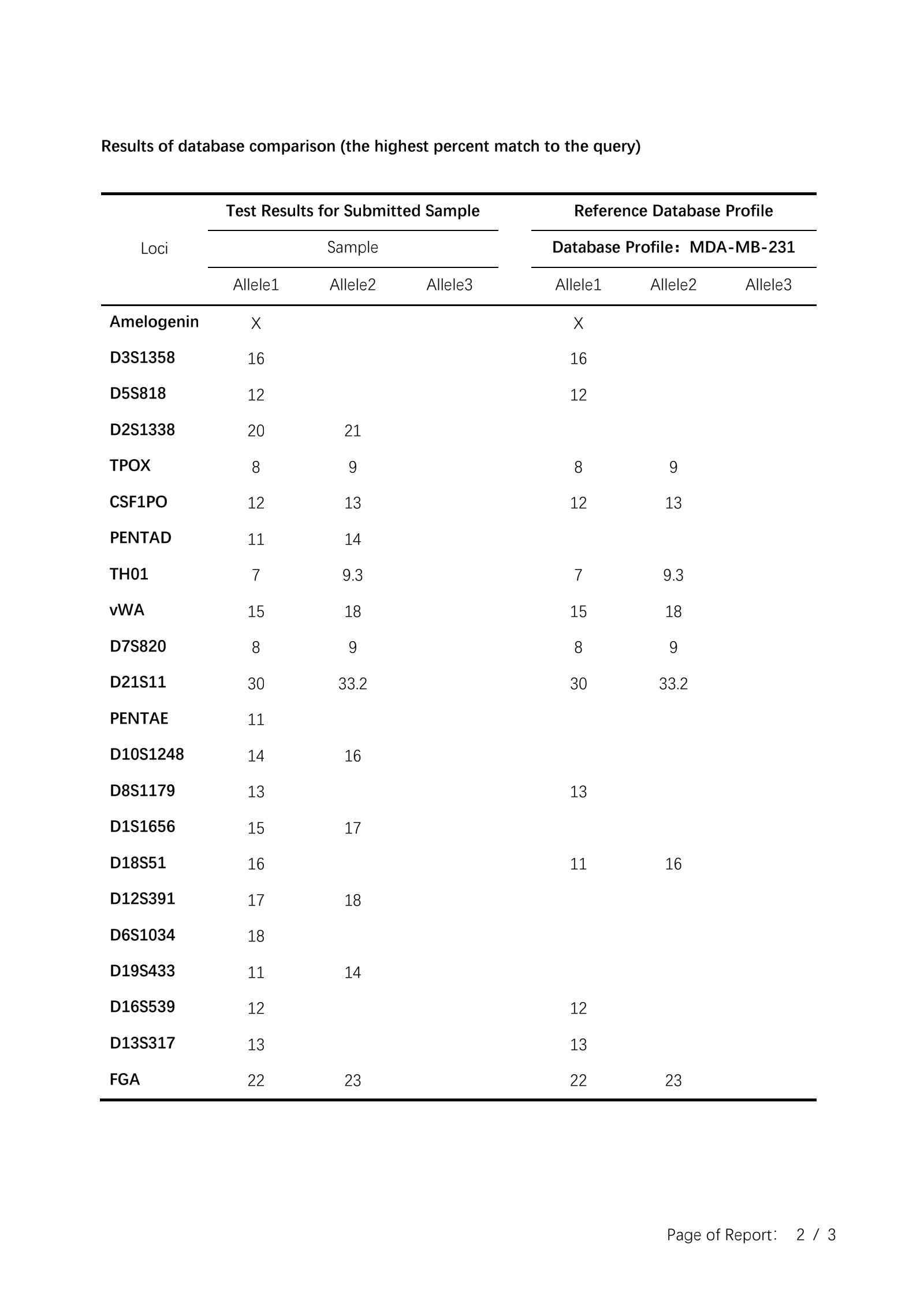


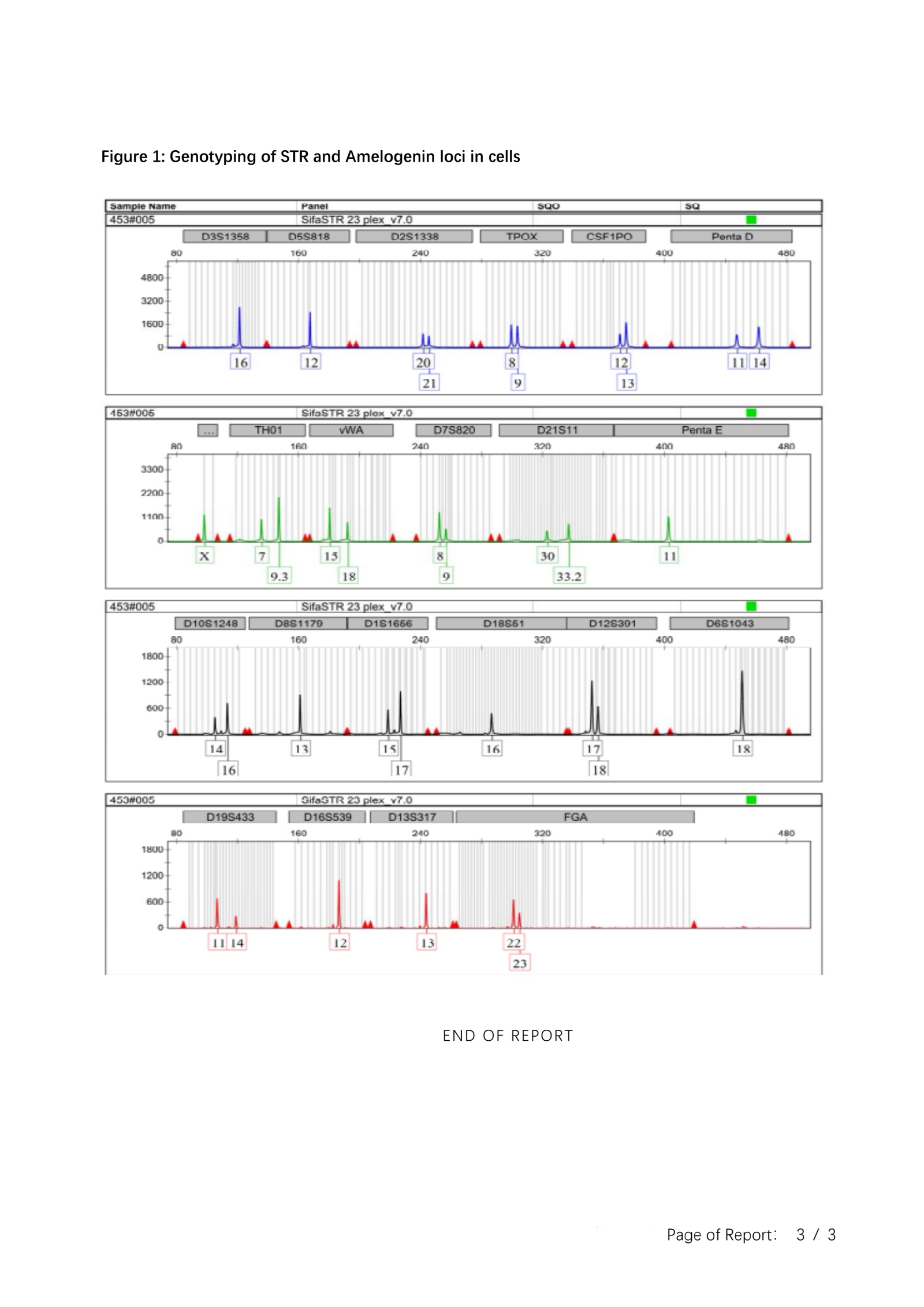


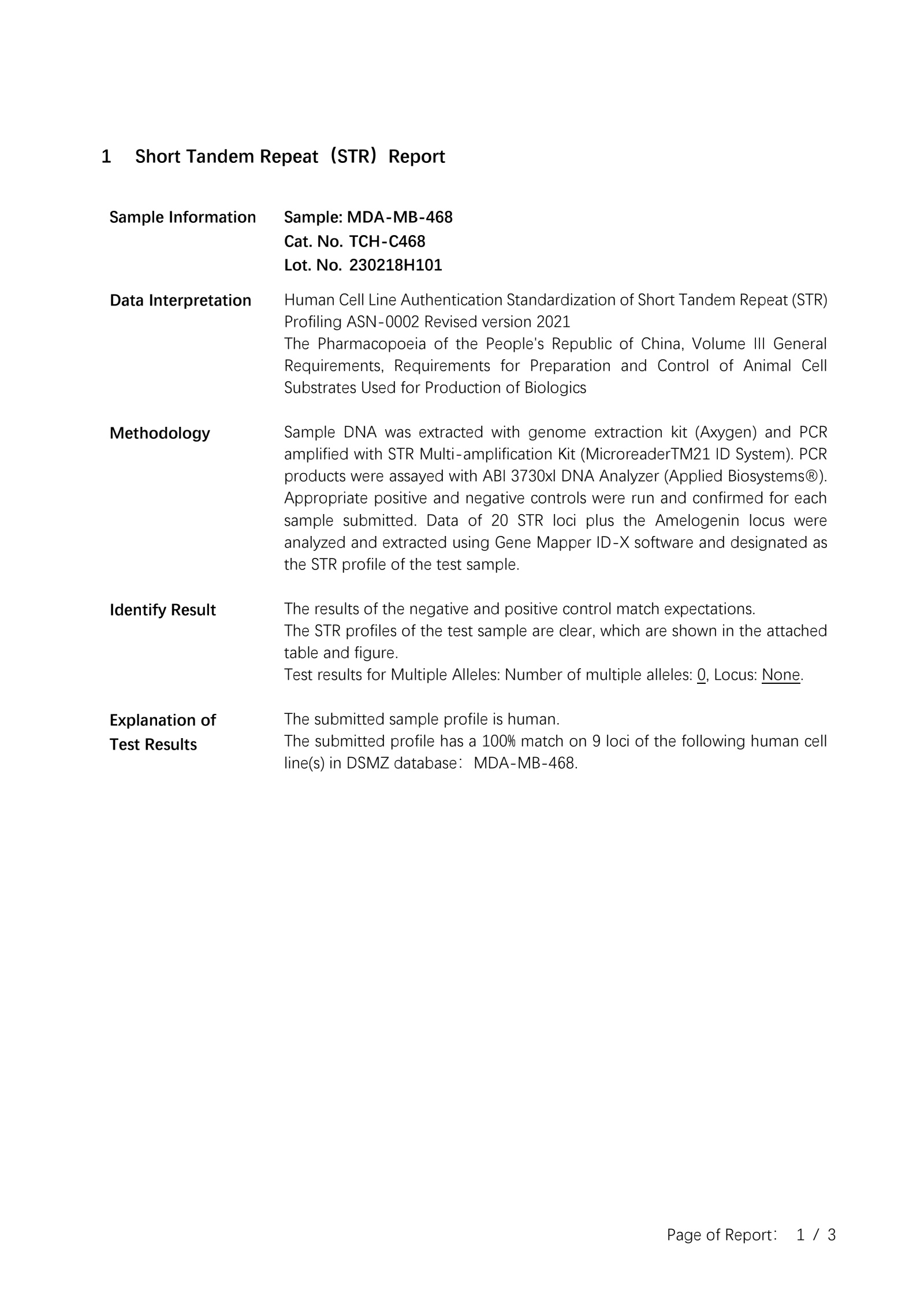


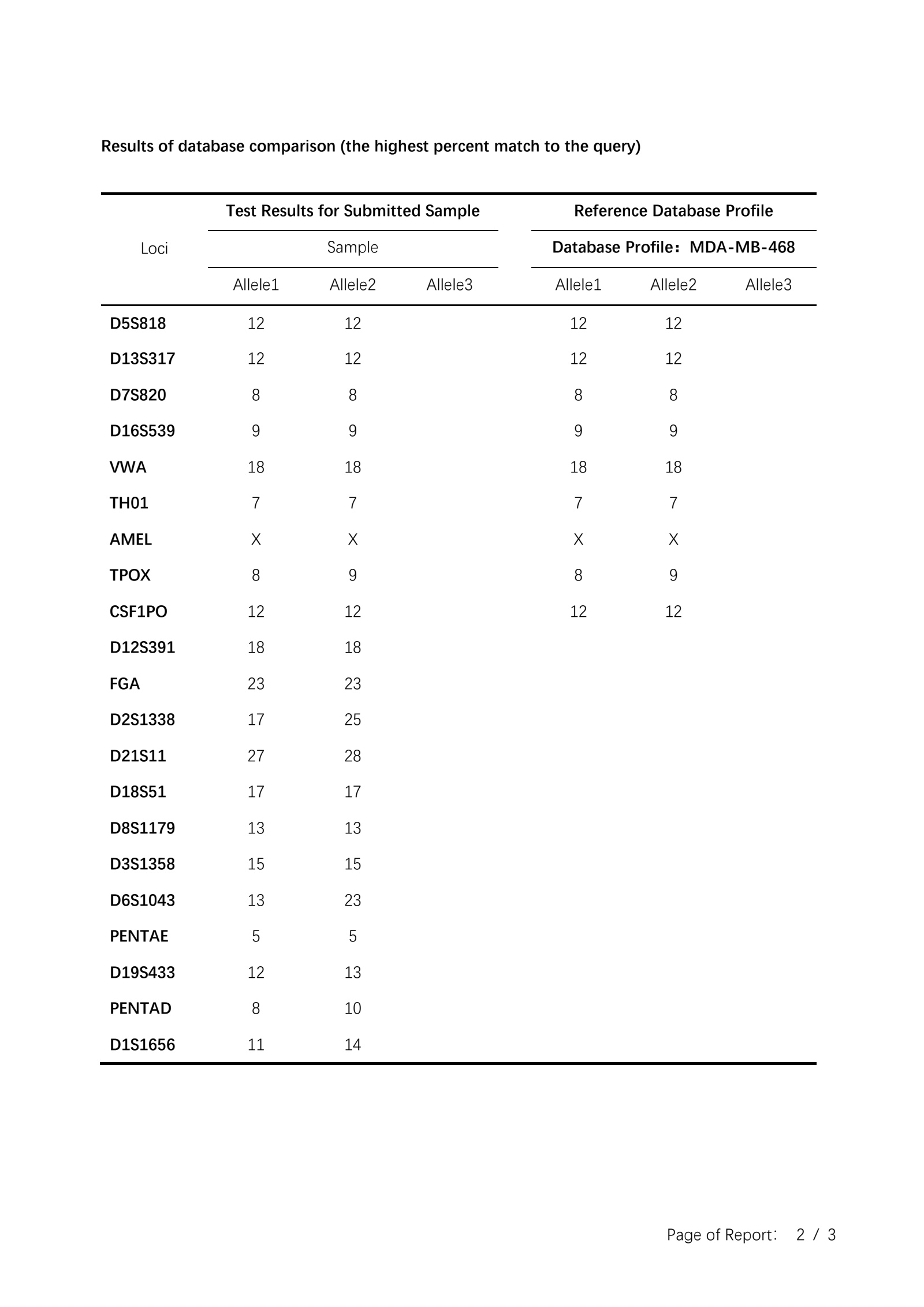


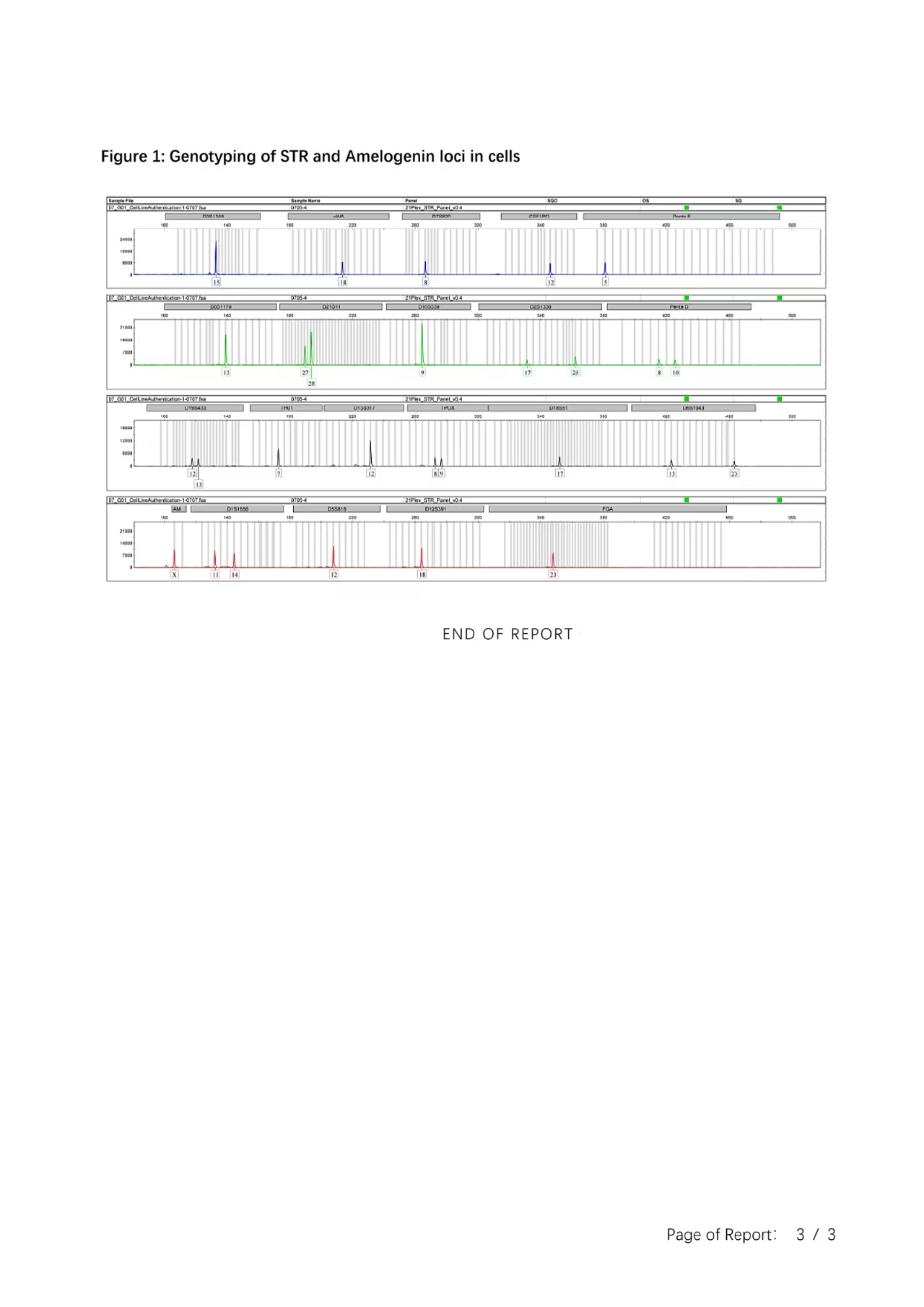


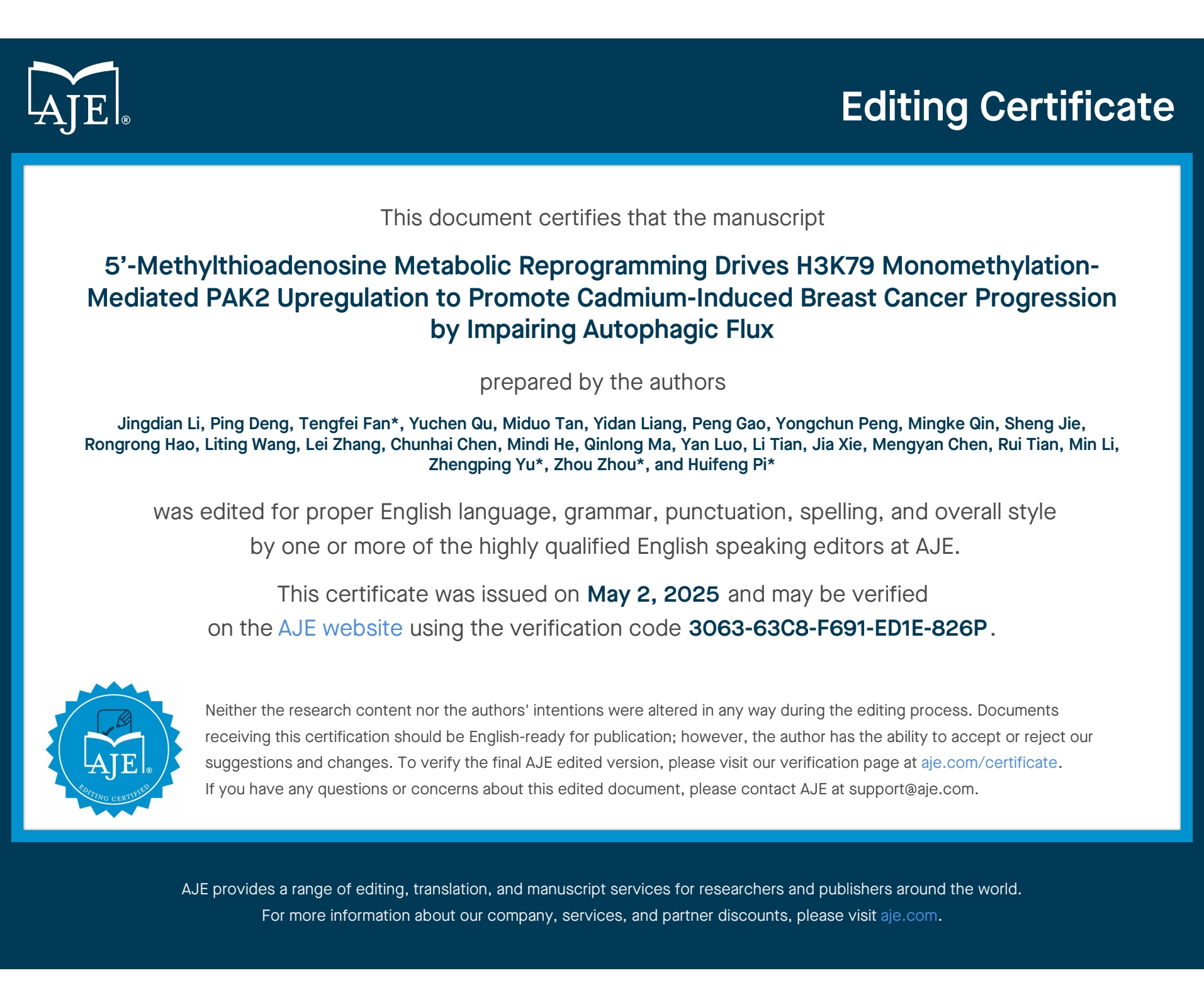

Supplement: Supplementary file 1 — Supporting Information [file ADVS-12-e00941-s001.docx]
